# Effects of Consumer-Wearable Activity Tracker-Based Programs on Objectively Measured Daily Physical Activity and Sedentary Behavior Among School-Aged Children: A Systematic Review and Meta-analysis

**DOI:** 10.1186/s40798-021-00407-6

**Published:** 2022-01-31

**Authors:** Carolina Casado-Robles, Jesús Viciana, Santiago Guijarro-Romero, Daniel Mayorga-Vega

**Affiliations:** 1 Department of Physical Education and Sport, University of Granada, Granada, Spain; 2 Department of Didactic of Musical, Plastic and Corporal Expression, University of Jaen, Paraje de las Lagunillas, Campus de Las Lagunillas, Edificio Humanidades y Ciencias de la Educación I (D2-D136), 23071 Jaén, Spain

**Keywords:** Wearable activity tracker, Activity monitor, Fitness tracker, Behavior change, Self-monitoring, Intervention, Goal-setting, Children, Adolescents

## Abstract

**Background:**

The popularity of consumer-wearable activity trackers has led the scientific community to conduct an increasing number of intervention studies integrating them to promote physical activity (PA) and to reduce sedentary behavior (SB) levels among school-aged children. Therefore, the aim of the present study was to estimate the effects of consumer-wearable activity tracker-based programs on daily objectively measured PA and SB among apparently healthy school-aged children, as well as to compare the influence of participants’ and programs’ characteristics.

**Methods:**

Eligibility criteria were: (1) participants: apparently healthy school-aged children (< 18 years old); (2) intervention: aimed to promote PA and/or to reduce SB incorporating consumer-wearable activity trackers; (3) comparator: baseline measurements and/or a control/traditional group; (4) outcomes: objectively measured daily PA and/or SB levels; (5) study design: pre-experimental, quasi-experimental, and true-experimental trials. Relevant studies were searched from eight databases up to December 2020, as well as from four alternative modes of searching. Based on the Cochrane Risk-of-bias tool 2, the risk of bias was assessed following four domains: (1) randomization process; (2) missing outcome data; (3) measurement of the outcomes; and (4) selection of the reported results. Based on a comprehensive systematic review, meta-analyses of the Cohen’s standardized mean difference (*d*) and 95% confidence interval (CI) with a random-effects model were conducted to estimate the overall effects, as well as the within- and between-study subgroups analyses effects, of the programs on daily total steps, moderate-to-vigorous PA (MVPA), total PA and SB.

**Results:**

Forty-four publications (i.e., 45 studies) were included in the systematic review (5,620 unique participants; mean age = 12.85 ± 2.84 years) and 40 publications (i.e., 41 studies) in the meta-analysis. Programs had a mean length of 11.78 ± 13.17 weeks and most used a waist-worn consumer-wearable activity tracker (77.78% waist-worn; 22.22% wrist-worn). Programs characteristics were: goal-setting strategies (64.06%); participants’ logbooks (56.25%); counseling sessions (62.50%); reminders (28.13%); motivational strategies (42.19%); and exercise routine (17.19%). Results showed a statistically significant moderate favorable effect on daily total steps (*d* = 0.612, 95% CI 0.477–0.746), small favorable effect on daily MVPA (*d* = 0.220, 95% CI 0.134–0.307), trivial favorable effect on daily total PA (*d* = 0.151, 95% CI 0.038–0.264) and trivial unfavorable effect on daily SB (*d* = 0.172, 95% CI 0.039–0.305). Subgroups analyses showed a higher effect for daily total steps and daily MVPA levels in females and the physically inactive for daily total steps (*p* = 0.003–0.044). Programs with educational counseling and/or goal-setting strategies, as well as a greater number of strategies, were more effective for improving children’s daily total steps, and wrist-worn activity trackers were more effective than waist-worn trackers for improving their daily MVPA levels (*p* = 0.001–0.021).

**Conclusions:**

Consumer-wearable activity tracker-based programs seem to be effective in promoting school-aged children’s daily total steps and MVPA levels, especially for females and those that are physically inactive. These programs should include specific goal-setting, educational counseling, and wrist-worn trackers as especially effective strategies. However, due to the certainty of evidence being from “low” to “moderate”, future well-designed primary research studies about the topic are needed.

**PROSPERO:** CRD42020222363.

**Supplementary Information:**

The online version contains supplementary material available at 10.1186/s40798-021-00407-6.

## Key Points

- Consumer-wearable activity tracker-based programs improve school-aged children’s daily total steps and moderate-to-vigorous physical activity levels, especially for females and the physically inactive.
- Goal-setting, educational counseling, and wrist-worn trackers have been highlighted as particularly useful strategies.
- Further research is needed to determine the effectiveness of consumer-wearable activity tracker-based programs using robust designs with low risk of bias and also to compare the effect of intervention characteristics in the same study.

## Background

Childhood and adolescence are considered to be sensitive periods of life as they are the stages in which healthy lifestyle behaviors can be formed and become established [1]. Moreover, these behaviors could influence future adult health status and behavior [2]. Two important lifestyle behaviors during the school-aged children’s awake time are physical activity (PA) (i.e., any waking behavior consisting of bodily movement that requires energy expenditure, which can be categorized into a *continuum* from light to vigorous intensity) [3, 4], and sedentary behavior (SB) (i.e., any waking behavior characterized by a low energy expenditure) [4, 5]. Both PA and SB are independent key indicators of health and quality of life among school-aged children [4]. Specifically, regular moderate-to-vigorous PA (MVPA) is positively associated with several health-related markers in young people [4]. Moreover, school-aged children’s daily total PA levels (i.e., light-to-vigorous PA) and daily total steps have also been demonstrated to be potential health indicators [6]. Independently of daily PA levels, long periods of SB is considered a risky behavior associated with health problems in school-aged children [7]. The World Health Organization (WHO) [4] recommends that school-aged children achieve, on average, at least 60 min daily of MVPA across the week, and they also should limit the amount of time spent being sedentary. Unfortunately, nowadays worldwide most school-aged children (approximately 81%) are physically inactive (i.e., do not meet the PA recommendations) [8], and they spend most of their time in SB [9]. These levels are worrying because physical inactivity is one of the main risk factors for non-communicable disease and death worldwide, which makes the promotion of health-enhancing PA and SB levels (i.e., high levels of PA and low levels of SB) among young people a paramount priority public health challenge [10].

As a result of the high importance of this topic in public health, a large number of intervention programs have been carried out to promote school-aged children’s health-enhancing PA and SB levels, which include a wide variety of behavior change techniques [11, 12]. Among others, self-monitoring behavior is an essential technique for PA practice promotion [13], and consumer-wearable activity trackers (e.g., smartwatches, activity wristbands, or pedometers) could be ideal devices to track school-aged children’s PA and SB levels, providing them this important real-time feedback [14]. These consumer-wearable activity trackers are electronic devices worn on the body as an accessory used for monitoring and recording daily PA and fitness-related metrics and providing users with real-time behavioral feedback via the monitor display [14, 15]. They usually integrate an accelerometer or pedometer to track physical movements automatically. Their outputs are generally based on step counts, the amount and intensity of PA, energy expenditure, periods of inactivity, or heart rate [16, 17]. Furthermore, these consumer-wearable activity trackers have become increasingly popular over recent years being reflected in a great increase in sales and strong demand from society worldwide every year [18]. Moreover, these devices are considered the most plausible activity monitors to be used in public health [19]. The popularity of these devices has led the scientific community to carry out effective studies that integrate consumer-wearable activity trackers promoting health-enhancing school-aged children’s PA and SB levels [20, 21].

However, each of those effectiveness studies alone only constitutes a specific part of the total evidence about this topic. By contrast, systematic reviews and meta-analyses allow for an objective analysis of all the available evidence about a specific topic, making sense of the often conflicting results found and providing estimation with greater power and precision than each individual primary study [22, 23]. Specifically, the number of reviews focused on the use of wearable activity trackers to increase PA and reduce SB levels has grown exponentially in recent years. However, most of them have been carried out in adults [24–26] or clinical populations [16, 27], leaving little evidence on the effectiveness of wearable activity trackers in apparently healthy school-aged children.

To our knowledge, only Ridgers et al. [28], Böhm et al. [29], Cajita et al. [30], and Creaser et al. [31] reviews included intervention studies carried out with both apparently healthy school-aged children and those with diagnosed diseases (e.g., cancer or diabetes) for promoting PA with consumer-wearable activity trackers. However, those reviews have considerable limitations. Firstly, regarding inclusion criteria, the four systematic reviews included restrictions on the language of publication (only in English) and they also exclude some grey literature sources (e.g., conference abstracts, dissertations, or pilot studies) which could bias the results due to interventions with significantly larger effects being more likely to be published in English and journal papers [32–34]. Furthermore, the Böhm et al. [29] and Cajita et al. [30] reviews included date restrictions in the search (only from 2012 and 2009, respectively), and the Cajita et al. [30] review also presented a limitation regarding electronic databases selected, since they did not include main bibliographic databases (e.g., Web of Science, Scopus, or SPORTDiscus) [22, 34]. These limitations imply that the systematic reviews were not carried out considering all the scientific evidence, and therefore, the estimation of the effect is not completely good.

Moreover, these four systematic reviews included studies with self-reported measured PA levels (i.e., questionnaires). However, previous evidence has demonstrated that self-reported measures of children and adolescents are poorly correlated with objectively measured PA and SB levels [35, 36]. Furthermore, the four systematic reviews also included studies measuring only part-time days (e.g., physical education lessons or school recess), which does not adequately reflect the effect on daily school-aged children’s PA and SB levels. Lastly, both the Ridgers et al. [28] and Böhm et al. [29] systematic reviews did not include pedometer-based studies as they did not consider them as consumer-wearable activity trackers. However, following common consumer-wearable activity trackers’ scientific definitions, pedometers should be included [15, 16, 37]. All the above-mentioned reasons could explain the limited evidence found (i.e., low number of publications) in their reviews, due to the inclusion of only five [28, 29] or two studies in their analyses [30]. Regarding the most recent Creaser et al.'s [31] review, it included more publications (i.e., 24 studies), but if only effectiveness studies with apparently healthy school-aged children and objectively measured daily PA and SB levels were considered, this would decrease to 11 studies. Moreover, it should be noted that Cajita et al. [30] conducted only a scoping review, whose objective was only to provide an overview of the available research evidence without producing a summary answer to a discrete research question [38]. Finally, as far as we know, there is no previous meta-analysis examining the effects of consumer-wearable activity tracker-based programs on daily objectively assessed PA and SB among school-aged children. Conducting a meta-analysis could summarize the effectiveness of those interventions in an overall statistical synthesis (effect size) rather than taking the results of each primary study separately. Therefore, it allows for improving the precision of the results by the estimation of the effect size and direction, and clarifying whether or not the effect size is consistent across studies. Thus, also based on study design and risk of bias assessment, the meta-analysis allows for assessing the strength of the evidence [34, 39].

Consequently, the main purpose of the present systematic review and meta-analysis was to estimate the effects of consumer-wearable activity tracker-based programs on daily objectively measured PA and SB among apparently healthy school-aged children. A secondary purpose was to compare the influence of school-aged children’s characteristics (i.e., sex, age group, and PA status) and the intervention programs characteristics (i.e., duration, worn-type activity tracker, goal-setting, diary, counseling, reminders, motivational strategies, and exercise) on the effects of the consumer-wearable activity tracker-based programs on daily objectively measured PA and SB.

## Methods

### Protocol and Registration

The review protocol was registered with the International Prospective Register for Systematic Reviews (PROSPERO, CRD42020222363, https://www.crd.york.ac.uk/prospero/display_record.php?RecordID=222363. The present systematic review and meta-analysis was based on the methodology described in previous reference literature such as the PRISMA guidelines [40] (see Additional File 1 for the PRISMA checklist) and Cochrane Handbook for Systematic Reviews of Interventions [34], among other important references [22, 39]. Firstly, a reproducible, transparent, and comprehensive systematic review was performed to identify, select, and synthesize all the relevant studies. Then, a meta-analysis was performed to provide more precise estimates of the effects than those derived from the primary studies.

### Eligibility Criteria

The eligibility criteria for including the retrieved studies in the systematic review were the following: (1) participants: apparently healthy children and adolescents (i.e., populations with diagnosed diseases/conditions were excluded) aged from 5 to 18 years (although the study was also included if the participants were up to 24-years-old but the average age was under 18, as they are considered “young people” by the WHO [41]); (2) Intervention: studies that examined the effect of programs to promote PA and/or to reduce SB incorporating consumer-wearable activity trackers (i.e., pedometers, smartwatches, fitness wristbands, or similar; smartphones applications were not included) alone or combined with other strategies (i.e., goal-setting, diaries, counseling, reminders, motivational strategies, or exercise routine) with a duration of at least three weeks were included [42]; (3) Comparator: The design must include at least one measure to compare intervention effects (i.e., baseline measurements in single-group designs and/or a control group with no intervention or with usual treatment without consumer-wearable activity trackers). Thus, interventions with only one experimental group and only post-intervention measures were not included; (4) outcomes: studies that assessed the effect of the programs on the objectively measured daily PA and/or SB levels were included (i.e., objective measures such as the same consumer-wearable activity trackers used in the intervention or research-grade activity trackers such as accelerometers, excluding self-reported measures). Furthermore, only whole day (awake) time-based timeframes such as a whole week, weekdays, and/or weekend days were included (i.e., part-time days such as physical education lessons, school recess, or leisure time were not included); (5) study design: Any kind of experimental designs including, but not limited to, pre-experimental trials (e.g., non-controlled trials with one-group pre–post-intervention design); quasi-experimental trials (e.g., cluster-randomized controlled trials or non-randomized controlled trials), and true-experimental trials (e.g., randomized controlled trials) were included.

### Data Sources and Search Strategy

The databases search following the search strategies and the download and collection in the reference manager was completed in December 2020. This search included the following eight electronic bibliographic databases: Web of Science™ (all databases), Scopus, PubMed, SPORTDiscus with Full Text, CINAHL, Cochrane Library, ProQuest Social Sciences Premium Collection, and ProQuest Dissertations & Theses Global™. The searches were carried out in the search field type “title, abstract, and keywords” or equivalent. The search terms used were based on three concepts: (1) consumer-wearable activity tracker; (2) intervention program, and (3) PA/SB. The terms of the same concept were combined with the Boolean operator “OR” and then the three concepts were combined using the Boolean operator “AND.” The keywords with more than one word were enclosed in quotes. No publication status, language, or date restrictions were imposed [22]. All search strategies are available in Additional File 2.

Then, additional studies were identified as follows (i.e., “snowballing”): (1) searching the reference lists of original studies, as well as some related study reviews and study protocols; (2) examining the reference citations and the researchers’ publications (first authors) in the Web of Science™ and Scopus databases; (3) contacting with the corresponding authors by email, and (4) screening the researchers’ personal lists in ResearchGate and Google Scholar (first authors). Any time a new study was found, all of these modes of searching were repeated until no new study appeared.

### Study Selection

After eliminating duplicates, the first author (CCR) undertook the potentially eligible records selection based on the screening of titles and abstracts to identify relevant studies. After that, two independent reviewers assessed the full texts for inclusion following the above-mentioned eligibility criteria (CCR/SGJ). Any disagreements regarding the inclusion of studies were resolved by consensus with a third reviewer (DMV). The inter-rater agreement between coders was substantial to almost perfect (proportion of agreement = 0.89; Cohen’s Kappa = 0.78).

### Data Extraction

From each selected study, data were coded using an ad hoc coding form developed by the research group and previously tested with a pilot sample of studies at the beginning of the review. This form included data about: (1) study characteristics (i.e., reference, publication date, date of the data collection, study design, sequence generation, suspicion of selective outcomes, and initial and final sample size); (2) participant characteristics (i.e., sex and age); (3) outcome measures pertaining to PA and/or SB (i.e., measurement moment, measurement time, kind of measurement instrument, and measurement score and units); (4) intervention characteristics (intervention length, kind of consumer-wearable activity tracker, kind of goal-setting, diary, counseling, reminders, motivational strategies, and exercise routine); and (5) results of the intervention for each group (i.e., initial and final group size, pre- and post-intervention standard deviation, and pre- and post-intervention means score or pre–post-intervention mean difference score). Complete coding form is available in Additional File 3.

If a study consisted of two or more study arms of which one of the intervention arms did not meet the inclusion criteria, data were only extracted from the study arms that met the inclusion criteria. In the event that the studies did not report some study feature, corresponding authors were contacted to retrieve it. If means and standard deviation were not retrieved, the scores were estimated and converted by the standard error, confidence intervals, *F, t* or *p* values [34]. Since median and interquartile range are often used when the data are asymmetrical, these values were not converted [34]. If any other study feature was not retrieved, the information was omitted. The sample size of each group, the mean scores of the pre- and/or post-intervention or mean difference scores of each group, and the measurement score of the dependent variable/s were considered to be critical for including the selected studies from the systematic review in the meta-analysis. In order to avoid removing studies from the meta-analysis, numerical data were extracted from their figures using the WebPlotDigitizer software [34], as was done with three studies [43–45]. Coding studies were carried out independently by two researchers (CCR/DMV). When doubt or disagreement occurred, a consensus was always achieved through discussion.

### Risk of Bias and Certainty of the Evidence

Based on the Cochrane risk-of-bias tool version 2 [34], the following methodological domains were assessed: (1) risk of bias arising from the randomization process; (2) risk of bias due to missing outcome data; (3) risk of bias in measurement of the outcomes, and (4) risk of bias in the selection of the reported results. Due to the nature of the selected studies (i.e., self-monitoring interventions to promote healthy habits with objective measurements), some risk of bias criteria were not considered. Firstly, blinding of participants and personnel was not included due to participants being always aware of their assigned intervention (i.e., they were using a consumer-wearable activity tracker), and researchers delivering the interventions were aware of the assigned intervention because they had to implement behavior change techniques in experimental groups. Therefore, the assessment of allocation concealment was also meaningless in this type of study since both participants and researchers would know which group they were assigned to during the intervention phase. Finally, blinding the outcomes assessment was not applicable because an objective method (i.e., research-grade accelerometers or consumer-wearable activity trackers) was used instead of subjective assessors or self-reported measures. Additional File 4 shows the algorithms followed for assessing methodological risk of bias in each domain.

Domains were judged and classified as “low risk,” “some concerns” or “high risk” of bias. Finally, overall risk of bias judgment was obtained as follows: “low risk” if low risk of bias was obtained for all domains; “some concerns” if at least one domain was judged as having some concerns, but not to be at high risk of bias for any domain; and “high risk” if at least one domain was judged as high risk or if two or more domains were judged as having some concerns [34].

Additionally, the overall certainty of the evidence was rated as “high,” “moderate,” “low,” or “very low” using the Grading of Recommendations Assessment, Development and Evaluation (GRADE) approach [46]. This assessment was based on the following five domains: risk of bias, inconsistency, imprecision, indirectness, and publication bias. A domain was classified as “no limitation” if no reason for downgrading the evidence was found, but the domain was classified as “serious” if a reason was found for downgrading the evidence (thus, downgrading the certainty rating by one level). The reasons for considering the domains as “serious” were: (a) risk of bias: Most of the studies (i.e., > 50%) were classified as “high” risk of bias; (b) inconsistency: high level of heterogeneity (i.e., *I*^2^ > 75%) was found; (c) imprecision: The confidence interval was wide including both the possibility of trivial effect (i.e., *d* < 0.20) and large effect (i.e., *d* ≥ 0.80); (d) indirectness: Most studies (i.e., > 50%) addressed a restricted version of the main review question in terms of population, intervention, comparator or outcomes; and (e) publication bias: Egger’s test was statistically significant and the impact of publication bias was large (i.e., the number of additional studies with no effect that would be needed to increase the *p* value over a statistically insignificant effect (i.e., *p* > 0.05) was low following the fail-safe *N* analyses; or Trim and Fill method trimmed many studies with an adjusted value for effect size different to the observed values).

### Data Analyses

The meta-analyses were performed using the software Comprehensive Meta-Analysis version 3.3.070 for Windows (Biostat, Englewood, USA). This software allows multiple data entry formats according to each study design. Therefore, considering the data provided by each study, the input method that best suited each study was chosen. Specifically, the option “one group with pre- and post-data” was used for pre-experimental trials, while the options “unmatched groups with pre- and post-data in each group,” “unmatched groups, mean change in each group,” or “unmatched groups, post-data only in each group” were used for controlled trials. The significance level was set at *p* < 0.05.

If a single study reported data for the whole sample and separately by different subsamples (e.g., children and adolescents), only the whole sample was used. Moreover, when in the same study there were different options for the same outcome (e.g., data from consumer-wearable activity trackers and research-grade activity trackers, or different measurement moments during the program) only the best option was selected (e.g., research-grade activity tracker or the last measurement as post-intervention). When a study had more than one activity tracker-based intervention group, each group was included in the analysis individually. The studies carried out with a small sample (defined as less than 10 participants per group) were not included in the meta-analysis [47].

#### Effects Sizes Computation

A detailed description of the data analyses carried out in the present meta-analysis can be found elsewhere [39]. Meta-analyses of the Cohen’s *d* standardized mean difference and 95% confidence interval with a random-effects model were conducted to obtain the intervention program effects on: (a) daily total steps; (b) MVPA; (c) total PA; and (d) SB. Based on Cohen’s [48] benchmarks, effect size values of *d* < 0.20 were considered as trivial, 0.20–0.49 as small, 0.50–0.79 as moderate, and ≥ 0.80 as large. Moreover, since daily total steps and MVPA in minutes work with meaningful scales, the Mean Difference (*D*) with a random-effects model was also conducted [34, 39]. Regarding total PA and SB, *D* analysis could not be conducted due to the low number of studies using the same scales within each variable. Positive *d* and *D* values mean that the program favorably increased participants’ daily PA, but it also means an unfavorable increase in their SB levels.

#### Publication Bias

Firstly, an exhaustive systematic review was carried out to avoid availability bias. Afterward, a deep examination of the selected studies was carried out to avoid any potential duplication of the information retrieved. Similarities between publications of the same authors, with the same values and/or the same sample size were examined. When the selected publications had full or partial duplicated information, these particular values were not analyzed. Then, to visually identify the impact of any potential publication bias, the funnel plots and the Egger’s test [49] were carried out for daily total steps and MVPA. Moreover, for assessing the impact of any potential publication bias, the Orwin’s fail-safe *N* analyses [50] (criterion for a “trivial” *d* = 0.10; mean *d* in missing studies, *d* = 0.00) [48], the Duval and Tweedie’s Trim and Fill method [51] (assuming missing studies in the left of the mean), and a cumulative meta-analysis sorted by larger study size were computed [39]. Due to the limited number of studies found for total PA and SB (*k* = 8), the publication bias analyses could not be carried out for these variables [47].

#### Heterogeneity and Subgroups Analyses

The presence of statistical heterogeneity in the estimation of the effect sizes of the program was examined with the *I*^2^ statistic. The thresholds for its interpretation were: Values up to 40% were considered not important, up to 75% moderate, and more than 75% high heterogeneity [34].

Based on a priori hypothesized moderators, subgroups analyses were also carried out to test the effect of the intervention regarding: (a) individuals’ characteristics (i.e., sex, age, and PA status); and (b) intervention program characteristics (i.e., duration, activity tracker type, goal-setting, kind of goal-setting, diary, counseling, reminders, motivational strategies, exercise routine, and number of behavior change strategies). Due to the limited number of studies found for total PA (*k* = 8) and SB (*k* = 8), the above-mentioned subgroups analyses were only carried out for the daily steps and MVPA variables following a partial hierarchical analysis approach. All subgroups analyses were carried out for between-study meta-analysis, while for within-study meta-analysis, only those with at least two units of analysis to compare with were performed. Finally, the influence of continuous covariates (i.e., age, PA status, duration, and number of behavior change strategies) on the intervention effect was also evaluated using meta-regression analyses.

#### Sensitivity Analysis

Finally, in order to evaluate the robustness of the main results, the following sensitivity analyses were performed: Cohen’s *d* with a fixed-effect model, Hedges’ *g* with a random-effects model, and Cohen’s *d* with a random-effects model separately for randomized controlled trial design or not. However, sensitivity analyses separately for studies classified by the overall risk of bias could not be carried out due to the low number of studies classified as “Low risk” (*k* = 1 for daily total steps; and *k* = 2 for MVPA, total PA, and SB).

## Results

### Study Selection

**Fig. 1:**
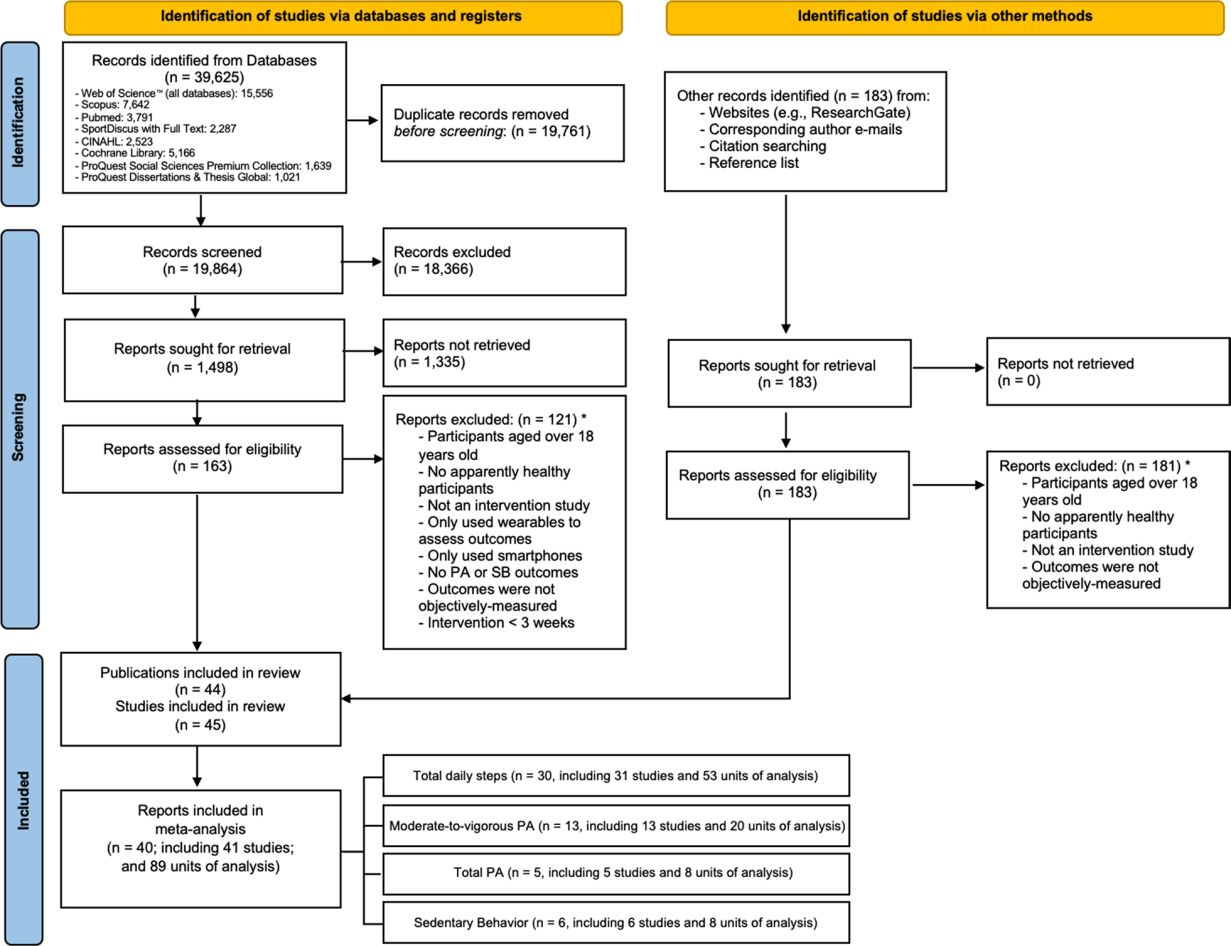
Flow diagram of the study selection process. PA = physical activity. *Note* The sum of publications included in the meta-analysis for each variable is greater than 40 because some studies reported outcomes for different variables. *The number of reports excluded for each reason has not been included because a report could have been removed for only one reason, but at the same time could meet many other reasons for exclusion, and therefore including them would not really give accurate information on the reports removed

Figure 1 shows the flow diagram of the study selection process. The search strategy identified 39,625 potentially relevant studies (19,864 studies after removing duplicates). Afterward, as a result of the studies of the Boolean-based database search, 183 additional records were identified through other sources. From the 1,498 records retrieved for a more detailed evaluation, 47 publications were a priori selected for meeting the selection criteria. However, only 44 unique publications (i.e., 45 studies) were finally included in the systematic review to avoid duplicated information. Finally, due to carrying out the study with a small sample and/or not reporting the critical values, only 40 publications (i.e., 41 studies) were included in the meta-analysis.

### Study Characteristics

Table 1 presents a summary of study characteristics included in the systematic review. Across the 44 included publications (i.e., 45 studies), in total 5,620 unique participants were included in all studies (3,914 = intervention; 1,706 = control). Samples were composed of a median of 84.50 initial participants, ranging from 20 [52] to 496 [53]. The participants’ mean age was 12.85 ± 2.84 years (range: 5–18 years). Regarding sex, 32 studies were conducted with males and females, while eight studies included only females and five studies included only males.

**Table 1:** Characteristics of included studies in the systematic review

Study	Participants characteristics			Study design	Intervention characteristics									Outcome parameter (instrument)^e^
	Number of participants^a^	Age^b^	Sex^c^		Tracker	Duration^d^	Group	Goal-setting	Diary	Counseling	Reminder	Motivation	Exercise	
Aksornsri [63]^f^	EG1: 40 (39)	11.4 (8–13)	Both	Non-CT	Wrist	4	EG1	Static	No	Yes	Yes	No	Yes	Steps (wearable)
Baldursdóttir [64]^f^	CG: 57 (54); EG1: 61 (57); EG2: 58 (57)	16.0 (15–16)	Both	CRCT	Hip	3	EG1	Adaptive	Yes	Yes	No	No	No	Steps (wearable)
							EG2	Adaptive	No	Yes	No	No	No	
Baldursdottir et al. [54]^f^	CG: 46 (27); EG1: 38 (26)	16.0 (16–16)	Both	CRCT	Hip	3	EG1	Adaptive	Yes	Yes	Yes	Yes	No	Steps (wearable)
Bronikowski et al. [56]^f^	EG1: 100 (100); EG2: 93 (93)	14.8 (10–17)	Both	Non-CT	Wrist	8	EG1	Static	No	No	No	No	No	Steps (wearable)
							EG2	No	No	No	No	No	No	
Corepal et al. [65]^f^	CG: 82 (64); EG1: 142 (126)	13.0 (12–14)	Both	CRCT	Hip	22	EG1	Adaptive	Yes	Yes	No	Yes	No	MVPA; SB; Steps; and Total PA (accelerometer)
Corr et al. [66]^f^	EG1: 31 (17)	14.0 (12–16)	Females	Non-CT	Hip	6	EG1	Adaptive	Yes	Yes	Yes	Yes	No	Steps (wearable)
Corr and Murtagh [67]^f^	EG1: 31 (10)	16.0 (15–17)	Females	Non-CT	Hip	6	EG1	Adaptive	Yes	Yes	No	No	Yes	Steps (wearable)
Duck et al. [68]^f^	CG: 18 (14); EG1: 17 (13)	9.2 (9–10)	Both	CRCT	Wrist	10	EG1	No	No	No	No	Yes	No	MVPA (accelerometer)
Duncan et al. [69]^f^	EG1: 59 (59)	10.7 (10–11)	Both	Non-CT	Hip	4	EG1	Static	Yes	Yes	No	Yes	No	Steps (wearable)
Ermetici et al. [55]^f^	EG1: 181 (167)	12.5 (11–15)	Both	Non-CT	Hip	84	EG1	No	No	Yes	Yes	No	No	Steps (wearable)
Evans et al. [70]^f^—Study 1	EG1: 32 (32)	10.0 (10–11)	Both	Non-CT	Hip	4	EG1	Static	Yes	Yes	No	Yes	No	Steps (wearable)
Evans et al. [70]^f^—Study 2	CG: 10 (10); EG1: 19 (19); EG2: 13 (13)	12.3 (11–12)	Both	Non-RCT	Wrist	6	EG1	Static	Yes	Yes	No	Yes	No	Steps and MVPA (accelerometer)
							EG2	No	No	No	No	No	No	
Eyre et al. [60]^f^	CG: 40 (30); EG1: 94 (55)	10.0 (9–12)	Both	CRCT	Hip	6	EG1	Static	Yes	Yes	No	Yes	Yes	Steps (wearable)
Finkelstein et al. [71]^f^	CG: 138 (89); EG1: 147 (145)	8.2 (6–12)	Both	CRCT	Hip	36	EG1	Static	No	Yes	No	Yes	No	Steps (wearable)
Galy et al. [72]^f^	EG1: 24 (15)	11.9 (12–14)	Both	Non-CT	Wrist	4	EG1	No	No	Yes	No	Yes	Yes	Steps (wearable)
Gaudet et al. [43]^f^	CG: 23 (16); EG1: 23 (16)	13.0 (13–14)	Both	CRCT	Wrist	7	EG1	No	No	No	No	No	No	MVPA and SB (accelerometer); Steps and MVPA (wearable)
Grao-Cruces et al. [73]^f^	EG1: 142 (66)	11.4 (11–12)	Both	Non-CT	Hip	6	EG1	Static	Yes	No	No	Yes	No	Steps (wearable)
Groffik et al. [74]^f^	EG1: 71 (64)	17.9 (17–18)	Both	Non-CT	Hip	3	EG1	No	Yes	No	No	No	No	Steps (wearable)
Guagliano et al. [21]^f^	CG: 25 (23); EG1: 24 (18); EG2: 24 (15)	9.3 (7–11)	Both	CRCT	Hip	8	EG1	No	No	Yes	No	Yes	No	MVPA and SB (accelerometer)
							EG2	Adaptive	Yes	Yes	Yes	Yes	No	
Hardman et al. [75]	CG: 18 (15); EG1: 14 (14)	10.6 (10–11)	Females	CRCT	Hip	12	EG1	Adaptive	Yes	No	No	Yes	No	Steps (wearable)
Hardman et al. [76]^f^	EG1: 81 (51); EG2: 119 (67); EG3: 186 (118)	9.0 (7–11)	Both	Non-CT	Hip	17	EG1	No	No	No	No	No	No	Steps (wearable)
							EG2	Adaptive	Yes	Yes	Yes	Yes	No	
							EG3	Adaptive	Yes	Yes	Yes	Yes	No	
Horne et al. [44]^f^	CG: 53 (51); EG1: 47 (38)	10.0 (9–11)	Both	CRCT	Hip	16	EG1	Adaptive	Yes	Yes	Yes	Yes	No	Steps (wearable)
Jago et al. [62]^f^—Phase 1	CG: 55 (51); EG1: 65 (64)	13.0 (10–14)	Males	CRCT	Hip	9	EG1	Static	No	Yes	No	No	Yes	SB; MVPA; and Total PA (accelerometer)
Jago et al. [62]^f^—Phase 2	CG: 130 (88); EG1: 103 (86)													
Jauho et al. [77]	CG: 139 (38); EG1: 137 (25)	17.9 (17–18)	Males	RCT	Wrist	12	EG1	No	No	No	No	No	No	MVPA and SB (wearable)
Kantanista et al. [78]^f^	EG1: 26 (26); EG2: 56 (56)	17.2 (16–18)	Females	Non-CT	Hip	7	EG1	No	No	No	No	No	No	Steps (wearable)
							EG2	Adaptive	No	No	No	No	No	
Kerner et al. [79]^f^	EG1: 62 (28)	14.5 (14–15)	Both	Non-CT	Wrist	5	EG1	No	No	No	No	No	No	MVPA (accelerometer)
Larsen et al. [80]^f^	EG1: 21 (19)	14.7 (12–18)	Females	Non-CT	Hip	12	EG1	No	Yes	Yes	Yes	Yes	No	MVPA (accelerometer)
Leinonen et al. [53]^f^	CG: 246 (80); EG1: 250 (87)	17.8 (17–18)	Males	RCT	Wrist	26	EG1	Static	No	Yes	No	Yes	No	MVPA and SB (wearable)
Linck [81]^f^	CG: 22 (17); EG1: 22 (18)	16.6 (14–18)	Females	RCT	Hip	12	EG1	Adaptive	Yes	No	No	No	No	Steps (wearable)
Lubans and Morgan [82]^f^	CG: 66 (52); EG1: 50 (45)	14.2 (14–15)	Both	CRCT	Hip	8	EG1	Adaptive	Yes	Yes	No	Yes	Yes	Steps (wearable)
Lubans et al. [83]^f^	CG: 66 (52); EG1: 58 (50)	14.1 (13–18)	Both	CRCT	Hip	24	EG1	Adaptive	Yes	Yes	Yes	Yes	Yes	Steps (wearable)
Lubans et al. [61]^f^	CG: 50 (50); EG1: 50 (50)	14.3 (14–15)	Males	CRCT	Hip	24	EG1	Adaptive	Yes	Yes	No	No	Yes	Steps (wearable)
Macias-Cervantes et al. [84]	CG: 38 (30); EG1: 38 (32)	7.8 (6–9)	Both	RCT	Hip	12	EG1	Adaptive	No	Yes	No	No	Yes	Steps (wearable)
Morris et al. [85]^f^	CG: 72 (31); EG1: 82 (52)	9.9 (9–10)	Both	CRCT	Hip	6	EG1	Adaptive	No	No	No	No	No	MVPA; SB; and Total PA (accelerometer)
Newton et al. [86]^f^	EG1: 14 (14); EG2: 13 (13)	8.7 (6–10)	Both	Non-CT	Hip	12	EG1	Adaptive	No	No	No	Yes	No	Steps (wearable)
							EG2	Adaptive	No	Yes	Yes	Yes	No	
Remmert et al. [52]	EG1: 10 (6); EG2: 10 (9)	12 (12–12)	Both	Non-CT	Wrist	12	EG1	No	No	No	No	No	No	MVPA (accelerometer); Steps and MVPA (wearable)
							EG2	Adaptive	No	Yes	No	No	Yes	
Routen et al. [45]^f^	EG1: 17 (14); EG2: 26 (20); EG3: 25 (16)	11.2 (11–12)	Both	Non-CT	Hip	3	EG1	No	No	No	No	No	No	MVPA and Total PA (accelerometer)
							EG2	Adaptive	Yes	No	No	No	No	
							EG3	Adaptive	Yes	No	No	No	No	
Schofield et al. [87]^f^	CG: 30 (24); EG1: 27 (23); EG2: 28 (21)	15.8 (15–18)	Females	CRCT	Hip	12	EG1	Adaptive	Yes	Yes	Yes	No	No	Steps (wearable)
							EG2	Adaptive	Yes	Yes	Yes	No	No	
Shapiro et al. [88]^f^	EG1: 18 (13); EG2: 18 (7)	8.7 (5–13)	Both	Non-CT	Hip	8	EG1	Static	Yes	Yes	Yes	Yes	No	Steps (wearable)
							EG2	Static	Yes	Yes	No	Yes	No	
Shimon and Petlichkoff [89]^f^	CG: 62 (36); EG1: 72 (43); EG2: 60 (34)	13.1 (12–14)	Both	CRCT	Hip	4	EG1	Adaptive	Yes	Yes	No	No	No	Steps (wearable)
							EG2	No	Yes	No	No	No	No	
Shore et al. [90]^f^	EG1: 57 (46); EG2: 56 (46)	11.9 (11–12)	Both	Non-CT	Hip	6	EG1	Static	Yes	Yes	Yes	Yes	No	Steps (wearable)
							EG2	No	Yes	No	No	No	No	
Smith et al. [91]^f^	CG: 180 (154); EG1: 181 (139)	12.7 (12–14)	Males	CRCT	Hip	20	EG1	Adaptive	Yes	Yes	Yes	Yes	Yes	Total PA and MVPA (accelerometer)
Thompson et al. [92]^f^	CG: 40 (34); EG1: 40 (36); EG2: 40 (31); EG3: 40 (37)	15.2 (14–17)	Both	RCT	Hip	12	EG1	No	No	Yes	No	No	No	MVPA and Steps (accelerometer)
							EG2	No	No	Yes	Yes	No	No	
							EG3	No	No	Yes	Yes	No	No	
Wang [93]^f^	EG1: 34 (24); EG2: 32 (22)	13.5 (13–14)	Females	Non-CT	Hip	6	EG1	Adaptive	Yes	Yes	No	No	No	Steps (wearable)
							EG2	No	Yes	No	No	No	No	
Zizzi et al. [94]^f^	EG1: 84 (68); EG2: 81 (60)	16 (14–18)	Both	Non-CT	Hip	3	EG1	No	Yes	Yes	No	No	No	Steps (wearable)
							EG2	No	Yes	No	No	No	No	

CG = control group; EG = experimental group; CT = controlled trial; CRCT = cluster randomized controlled trial; RCT = randomized controlled trial; PA = physical Activity; MVPA = moderate-to-vigorous physical activity; SB = sedentary behavior.

^a^Number of participants are reported as “group: initial number (final number)”

^b^Age is reported as “mean (minimum–maximum)” in years

^c^Both means that includes males and females

^d^Duration is reported in number of weeks

^e^Wearable correspond to the same wrist-worn or hip-worn consumer-wearable activity trackers used in the intervention. Accelerometer correspond to research-grade activity trackers

^f^Studies included in the meta-analysis

Regarding the design of the studies, five were true-experimental trials (i.e., a randomized controlled trial design), 19 were quasi-experimental trials (i.e., a cluster-randomized controlled design or non-randomized controlled design), and 21 were pre-experimental trials (i.e., non-controlled design). Regarding the measurement instrument, 27 used a hip-worn consumer-wearable activity tracker, five a wrist-worn consumer-wearable activity tracker, and 11 a research-grade activity tracker, while two studies reported both wrist-worn consumer-wearable activity tracker and research-grade activity tracker measures.

The consumer-wearable activity tracker-based programs included had a mean length of 11.78 ± 13.17 weeks, but varied considerably from three [54] to 84 weeks [55]. Specifically, 44.44% of the included studies lasted more than eight weeks. Most of them used a waist-worn consumer-wearable activity tracker as a motivational tool in the program (*n* = 35 waist-worn; and *n* = 10 wrist-worn). Regarding the intervention characteristics, from the 64 experimental groups formed (along with the 45 studies), they included: A goal-setting strategy [20.31% (i.e., 13) and 43.75% (i.e., 28) of experimental groups used a static goal and an adaptive goal, respectively]; participants’ logbooks such as any diary in which school-aged children filled in their daily steps or minutes involved in PA, or any mobile app in which they synchronized their daily PA data and checked their progress [56.25% (i.e., 36) of experimental groups]; educational counseling sessions about PA-related knowledge, like benefits of regular PA practice, international guidelines, or how to overcome barriers to PA practice [62.50% (i.e., 40) of experimental groups]; reminders to persuade participants to move [28.13% (i.e., 18) of experimental groups]; motivational strategies such as the support of social networks like Facebook or the inclusion of challenges and competitions between groups [42.19% (i.e., 27) of experimental groups]; or exercise routines [17.19% (i.e., 11) of experimental groups].

### Risk of Bias and Certainty of the Evidence

Additional File 5 shows the risk of bias assessment for each included unit of analysis. For total daily steps, 60.38% (*n* = 32) of units of analysis were assessed as overall “high risk,” 37.74% (*n* = 20) as “some concerns,” and only 1.88% (*n* = 1) as overall “low risk.” Regarding MVPA, 80.00% (*n* = 16) was assessed as overall “high risk,” 10.00% (*n* = 2) as “some concerns,” and 10.00% (*n* = 2) as overall “low risk.” Concerning total PA, 75.00% (*n* = 6) was assessed as overall “high risk” and 25.00% (*n* = 2) as “some concerns.” Finally, for SB, 62.50% (*n* = 5) was assessed as overall “high risk,” 12.50% (*n* = 1) as “some concerns” and 25.00% (*n* = 2) as overall “low risk.”

Particularly, the risk of bias arising from the randomization process was the most problematic domain, with 21.35% of units of analysis being classified as “high risk” for not being a random design and 44.94% classified as “some concerns” due to being a non-controlled design or presenting imbalances regarding sample size or PA status between control and experimental groups. Moreover, the bias in measurement of the outcome domain was also problematic with half of the unit of analysis classified as “high risk” (13.48%) or “some concerns” (37.08%) due to inappropriate method of measuring (e.g., studies using consumer-wearables as measurement instrument instead of research-grade).

Moreover, based on the GRADE assessment (Additional File 6), the effect size of daily total steps was classified as “low” certainty of evidence due to both the risk of bias and inconsistency domains presenting serious limitations (i.e., most studies were classified as “high risk” of bias, and a substantial level of heterogeneity was found, respectively). Regarding MVPA, total PA and SB, the effect size was classified as “moderate” certainty of evidence due to serious limitations in the risk of bias domain (most studies were classified as “high risk” of bias).

### Publication Bias

#### Avoiding Duplicated Information

Although three publications met the selection criteria [56–58], they were not included in the systematic review and meta-analysis because the same data had been reported in other publications.

#### Identifying Publication Bias

**Fig. 2:**
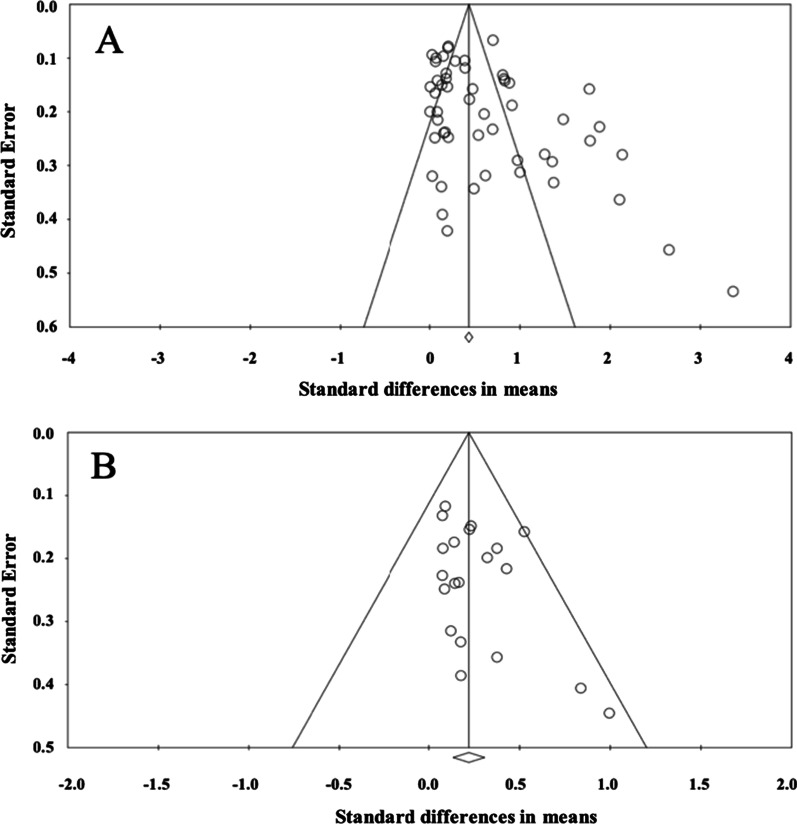
Funnel plots of standard error by standard differences in means (*d*) comparing consumer-wearable activity tracker-based programs effects on **A** daily total steps, and **B** moderate-to-vigorous physical activity

The visual assessment of funnel plots suggested that there was publication bias for daily total steps and MVPA (Fig. 2). In this sense, the results of the Egger’s test were statistically significant for daily total steps (*p* = 0.001) and MVPA (*p* = 0.045).

#### Assessing the Impact of Publication Bias

The results of Orwin’s fail-safe *N* analyses showed that the number of missing studies needed to bring the mean *d* values under a trivial value was unlikely, especially if the percentage of unlocated/located studies was considered, being 177 (333.96%) for daily total steps and 25 (125.00%) for daily MVPA. Furthermore, the results of Duval and Tweedie’s Trim and Fill method did not trim any study for daily steps and only two studies for MVPA in the standardized mean difference *d* analyses. Regarding mean difference *D* analyses, no study for daily steps and only one study for daily MVPA were trimmed. Moreover, the adjusted value for MVPA was similar to the observed values (*d* = 0.220 vs. 0.213; *D* = 5.583 vs. 4.824). Finally, regarding the cumulative meta-analysis plots sorted by larger study (Additional File 7), after some fluctuations in the first studies which may be due to chance [59], a fairly constant estimate of the effect over sample size was observed for total steps and MVPA. Although a large effect in the first primary study was found, the summary value was decreased after the 14th-to-15th study and the 2nd-to-3rd study for daily total steps and MVPA, respectively. Furthermore, neither a transient loss of formal significance nor a complete reversal of the initial association was found. Finally, it is worth mentioning that the addition of new primary studies did not materially change the estimates, so the final effect size values for these intervention programs seem to be quite robust.

### Effects Sizes

**Fig. 3:**
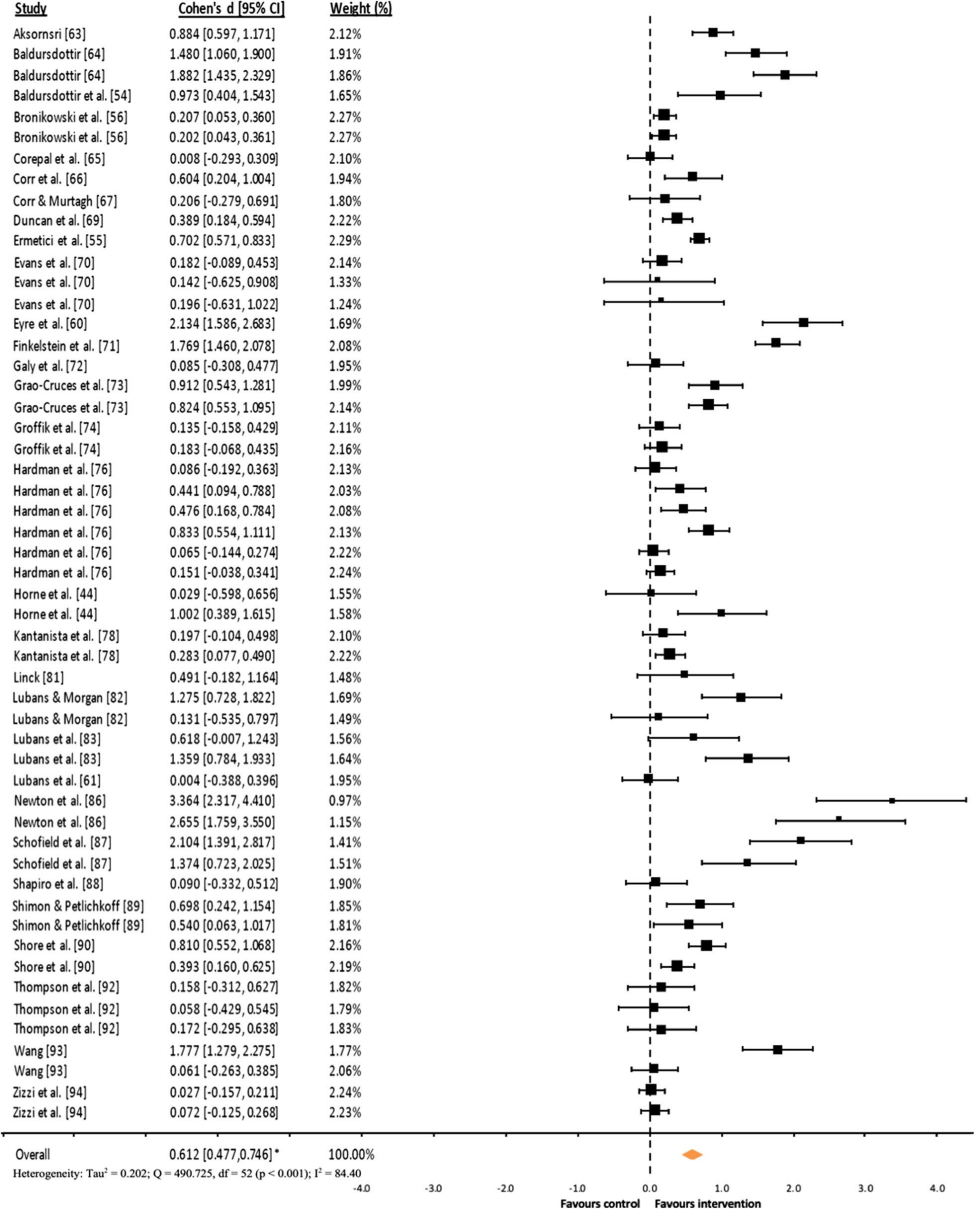
Forest plot of standardized mean differences (*d*) comparing consumer-wearable activity tracker-based programs effects on daily total steps. *Note* A positive *d* value means that the program favorably increased participants’ daily total steps; * *p* < 0.001

**Fig. 4:**
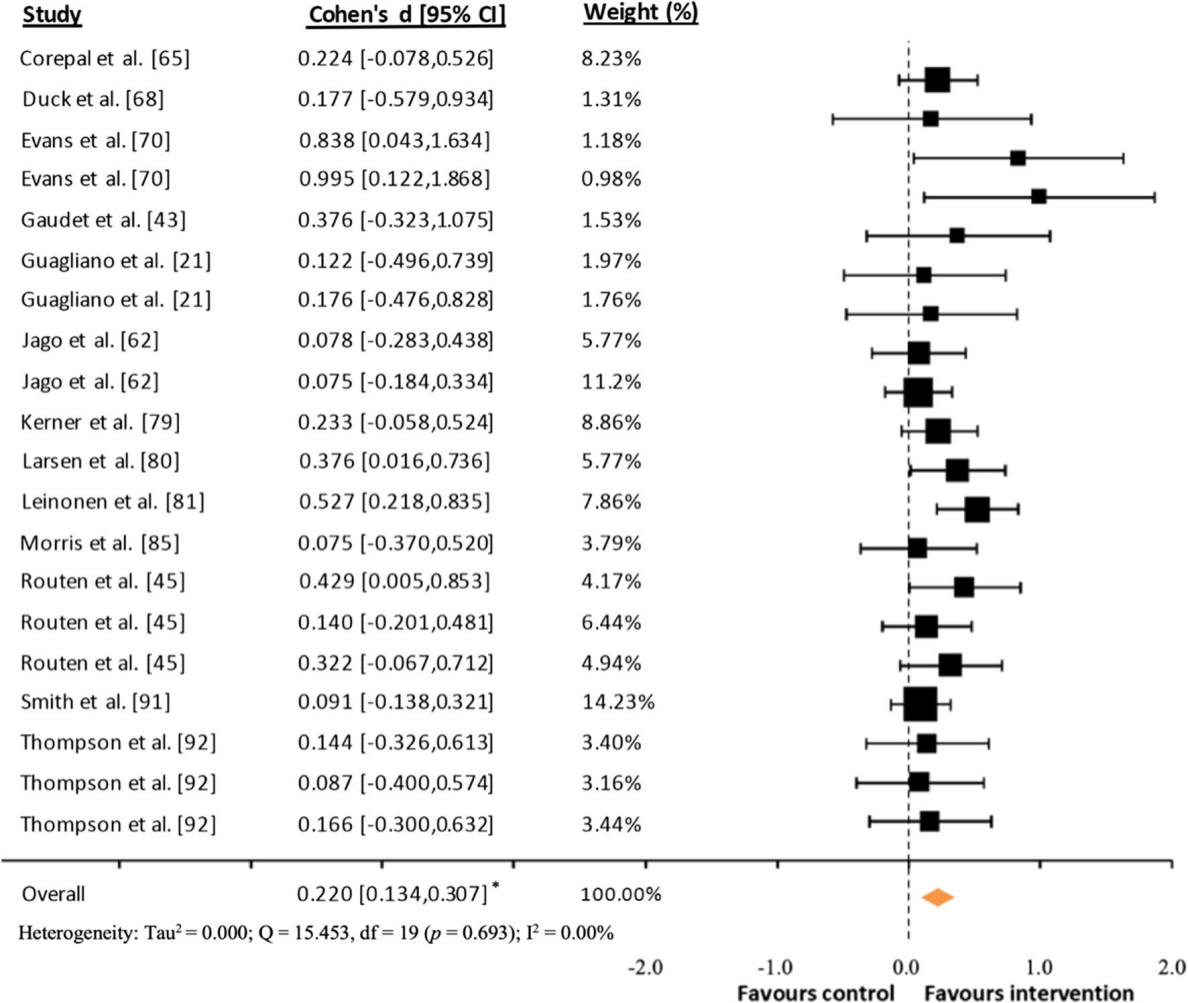
Forest plot of standardized mean differences (*d*) comparing consumer-wearable activity tracker-based programs effects on daily moderate-to-vigorous physical activity. *Note* A positive *d* value means that the program favorably increased participants’ daily moderate-to-vigorous physical activity; * *p* < 0.001

**Fig. 5:**
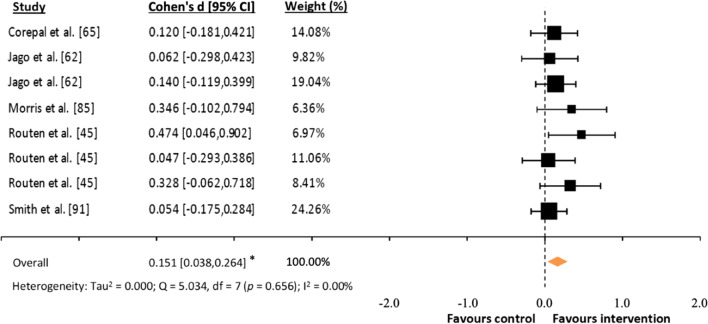
Forest plot of standardized mean differences (*d*) comparing consumer-wearable activity tracker-based programs effects on daily total physical activity. *Note* A positive *d* value means that the program favorably increased participants’ daily total physical activity; * *p* = 0.009)

**Fig. 6:**
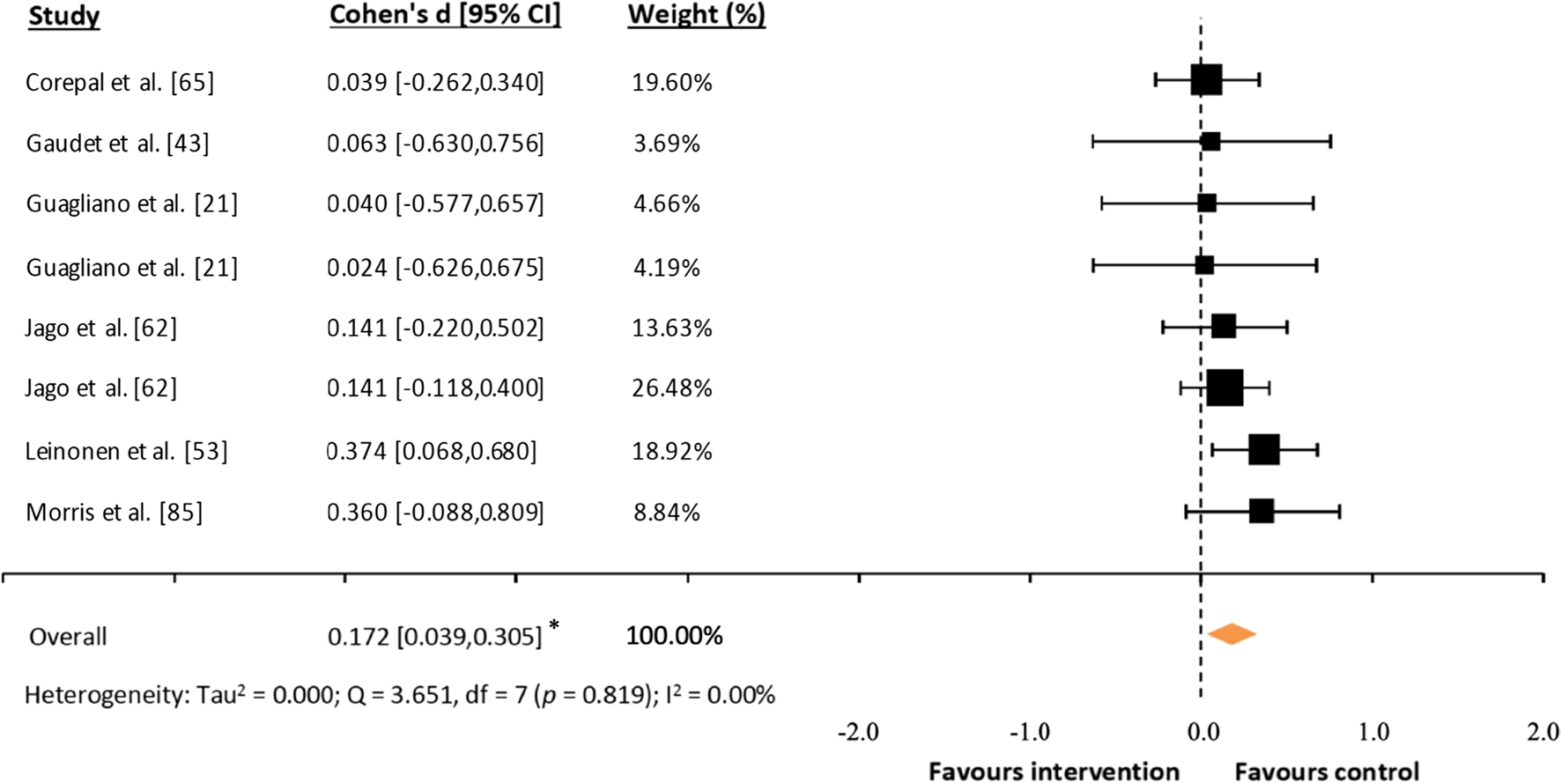
Forest plot of standardized mean differences (*d*) comparing consumer-wearable activity tracker-based programs effects on daily sedentary behavior. *Note* A positive *d* value means that the program unfavorably increased participants’ daily sedentary behavior; * *p* = 0.011

The results of the effects sizes showed that consumer-wearable activity tracker-based programs had a statistically significant moderate favorable effect on daily total steps (*d* = 0.612, 95% CI 0.477–0.746, *p* < 0.001; Fig. 3), small favorable effect on daily levels of MVPA (*d* = 0.220, 95% CI 0.134–0.307, *p* < 0.001; Fig. 4), and trivial favorable effect on daily levels of total PA (*d* = 0.151, 95% CI 0.038–0.264, *p* = 0.009; Fig. 5). Moreover, the results of effect sizes carried out with mean difference showed significant favorable effects on daily total steps (*D* = 1,692.792, 95% CI 1,322.522–2,063.061, *p* < 0.001) and MVPA (*D* = 5.583, 95% CI 2.527–8.640, *p* < 0.001). However, the programs had a statistically significant trivial unfavorable effect on daily SB (*d* = 0.172, 95% CI 0.039–0.305, *p* = 0.011; Fig. 6).

Furthermore, regarding the heterogeneity of the results, for daily MVPA (only for *d* analysis), total PA and SB heterogeneity was not found (*I*^2^ = 0.00%). However, high heterogeneity was found for daily total steps (*I*^2^ = 89.40% and 93.36% for *d* and *D* analyses, respectively), and moderate heterogeneity for daily MVPA (*I*^2^ = 54.23%, only for *D* analysis). Therefore, together with the fact that the number of studies with daily total PA and SB was low (*k* = 8), follow-up subgroups analyses were conducted only for daily total steps and MVPA.

### Subgroups Analyses

#### Subgroups Analyses for Daily Total Steps

Additional File 8 shows the results of the within-study subgroups analyses for the effect of the consumer-wearable activity tracker-based programs on the daily total steps among school-aged children. The initial values of school-aged children’s accomplishment with PA recommendations was the only individuals’ characteristic that influenced the effect of the programs, being more effective in those physically inactive than in those physically active (*d* = 1.206 vs. 0.107; *p* < 0.001). Moreover, heterogeneity analyses showed that the effect of the program separately for accomplishment with PA recommendations was homogeneous (*I*^2^ = 0.00%).

Furthermore, Additional File 9 shows the results of the between-study subgroups analyses. Regarding the influence of the individuals’ characteristics, subgroups analysis showed that the program had statistically significantly more effect in females than in males (*d* = 0.636 vs. 0.266; *p* = 0.044) and in physically inactive (i.e., not meeting the daily PA recommendations) than in those that were physically active (*d* = 0.795 vs. 0.397; *p* = 0.003) for increasing daily total steps. As regards the influence of the intervention characteristics, programs with some kind of counseling were more effective than those without counseling (*d* = 0.711 vs. 0.407; *p* = 0.003); likewise, programs that included goal-setting were more effective than those that did not (*d* = 0.770 vs. 0.243; *p* < 0.001) for increasing school-aged children’s daily total steps. However, for the rest of the between-study subgroups comparisons no statistically significant differences were found (*p* > 0.05). According to heterogeneity analyses, the effect of these programs separately for sex, PA status, goal-setting and counseling was still moderate-to-high heterogeneous (*I*^2^ = 66.08–92.54%).

**Fig. 7:**
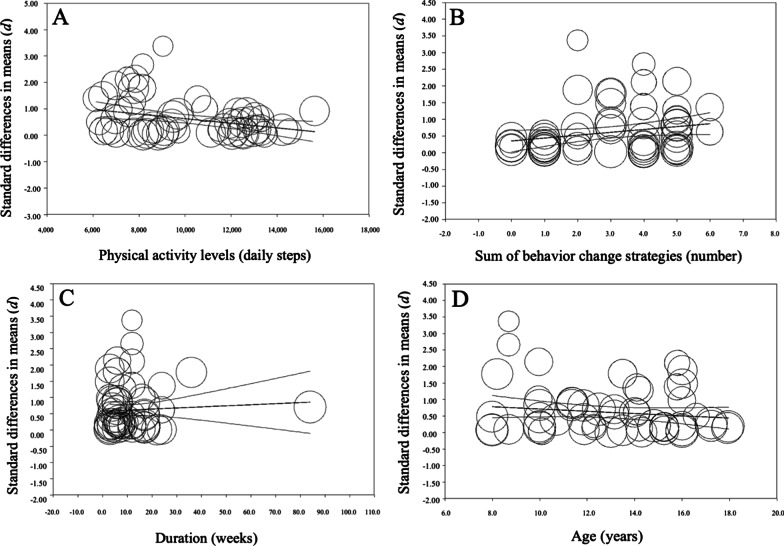
Meta-regression scatterplots of standard differences in means (*d*) comparing consumer-wearable activity tracker-based programs effects on daily total steps regarding **A** school-aged children’s physical activity levels, **B** strategies included in the program, **C** duration, and **D** school-aged children’s age

Moreover, meta-regression analysis showed that the effect of the intervention program was statistically significantly associated with PA status and number of strategies, with the effect being higher for daily total steps in less active school-aged children (*Q* = 10.83; *p* = 0.001; *R*^2^ = 0.07; *I*^2^ = 88.58%), and when a greater number of strategies were included in programs (*Q* = 5.19; *p* = 0.023; *R*^2^ = 0.02; *I*^2^ = 89.02%) (Fig. 7). However, no statistically significant associations were found between intervention duration (*Q* = 0.38; *p* = 0.536; *R*^2^ = 0.00; *I*^2^ = 89.04%) or age (*Q* = 2.17; *p* = 0.141; *R*^2^ = 0.01; *I*^2^ = 89.17%) and school-aged children’s daily total steps. Furthermore, meta-regression analysis of a more complex model including both significant explanatory variables together (i.e., PA status and number of strategies included), showed a statistically significant association with intervention effect on daily total steps, explaining a higher percentage of the variance than both explanatory variables alone (*Q* = 16.55, *p* < 0.001; *R*^2^ = 0.09; *I*^2^ = 89.40%).

#### Subgroups Analyses for Daily Moderate-to-Vigorous Physical Activity

Additional File 10 shows the results of the within-study subgroups analyses for the effect of the consumer-wearable activity tracker-based programs on the daily MVPA levels among school-aged children. Results showed no statistically significant differences for any subgroups comparisons (i.e., sex, goal-setting, diary, and reminders).

Furthermore, Additional File 11 shows the results of the between-study subgroups analyses. Regarding the influence of the school-aged children’s characteristics, subgroup analyses showed that the program had significantly more effect in physically inactive participants than in those physically active (*d* = 0.404 vs. 0.170; *p* = 0.046) for increasing daily MVPA levels. As regards the influence of the intervention programs’ characteristics, programs carried out with a wrist-worn activity tracker were more effective than those with a waist-worn activity tracker (*d* = 0.413 vs. 0.167; *p* = 0.021); moreover, programs that did not include any exercise routine were more effective than those that did (*d* = 0.283 vs. 0.083; *p* = 0.036). However, for the rest of the between-subgroups comparisons no statistically significant differences were found (*p* > 0.05). According to heterogeneity analyses, results by subgroups showed homogeneous results (*I*^2^ = 0.00–29.06%).

**Fig. 8:**
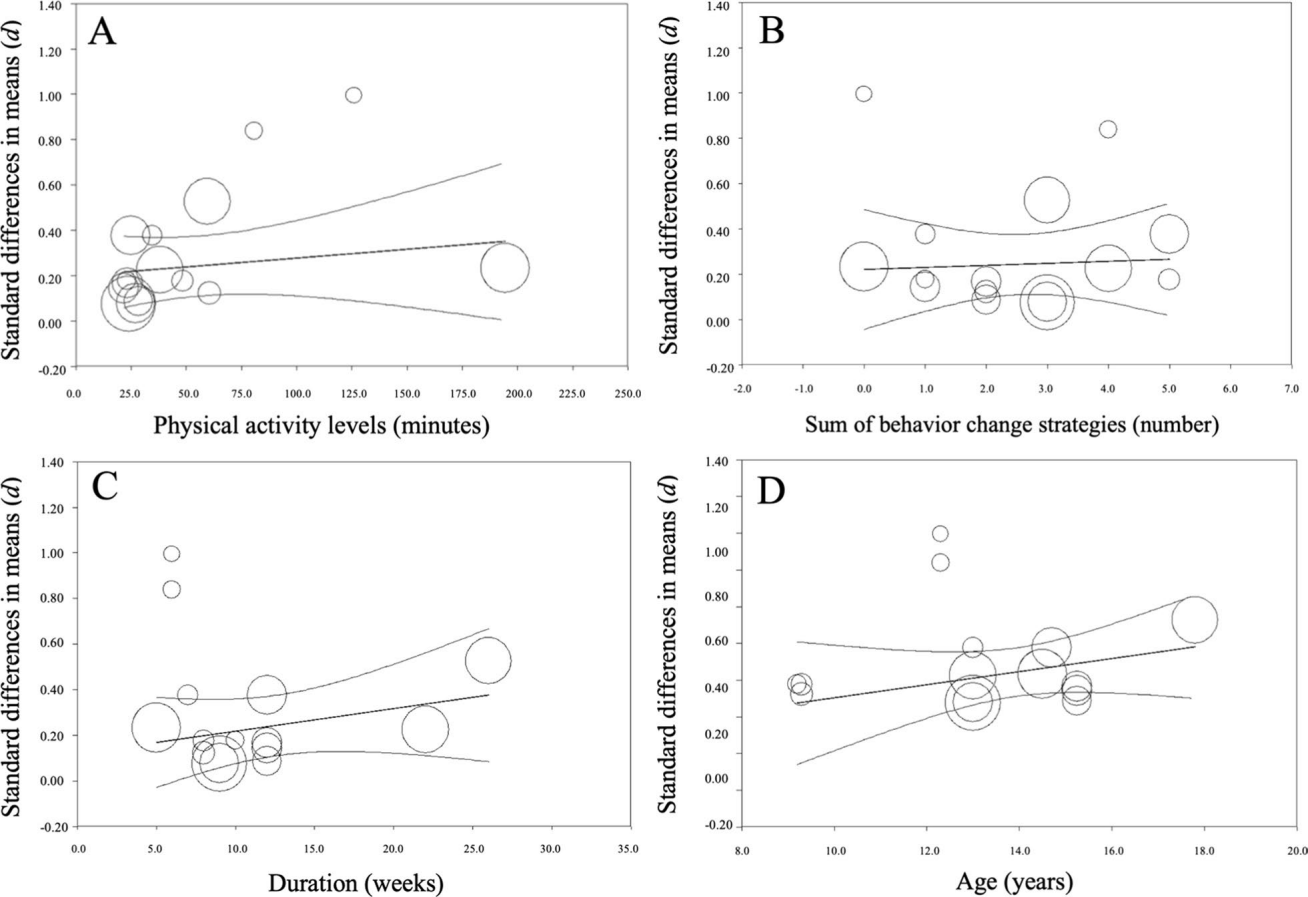
Meta-regression scatterplots of standard differences in means (*d*) comparing consumer-wearable activity tracker-based programs effects on daily moderate-to-vigorous physical activity regarding **A** school-aged children’s physical activity levels, **B** strategies included in the program, **C** duration, and **D** school-aged children’s age

Moreover, meta-regression analyses did not show statistically significant associations between PA status (*Q* = 0.65; *p* = 0.420; *R*^*2*^ = 0.00; *I*^*2*^ = 0.00%), number of strategies (*Q* = 0.06; *p* = 0.805; *R*^*2*^ = 0.00; *I*^*2*^ = 0.00%), intervention duration (*Q* = 1.55; *p* = 0.213; *R*^*2*^ = 0.00; *I*^*2*^ = 0.00%), or participants’ age (*Q* = 1.84; *p* = 0.175; *R*^*2*^ = 0.00; *I*^*2*^ = 0.00%) and school-aged children’s daily MVPA levels (Fig. 8).

### Sensitivity Analyses

The results of the sensitivity analyses for the overall effects sizes carried out with Hedges’ *g* random-effects model for daily total steps (*g* = 0.600, 95% CI 0.467–0.732, *p* < 0.001), MVPA (*g* = 0.218, 95% CI 0.133–0.302, *p* < 0.001), total PA (*g* = 0.150, 95% CI 0.039–0.261, *p* = 0.008) and SB (*g* = 0.171, 95% CI 0.038–0.303, *p* = 0.011) showed the same results as the main analysis carried out with Cohen’s *d* with a random-effects model. Similarly, overall effects sizes carried out with Cohen’s *d* with a fixed-effect model for daily total steps (*d* = 0.434, 95% CI 0.392–0.475, *p* < 0.001), MVPA (*d* = 0.220, 95% CI 0.134–0.307, *p* < 0.001), total PA (*d* = 0.151, 95% CI 0.038–0.264, *p* = 0.009) and SB (*d* = 0.172, 95% CI 0.039–0.305, *p* = 0.011) also showed the same results as the main analysis with the random-effects model.

Finally, results of the Cohen’s *d* with a random-effects model separately for randomized controlled trial design compared with non-randomized controlled trial design showed greater effects in randomized controlled trials for daily total steps (*d* = 0.859 vs. 0.465; *p* = 0.004). However, no statistically significant differences were found for MVPA (*p* = 0.104) or total PA (*p* = 0.328). Regarding SB, all the included studies were randomized controlled trials and the comparison could not be performed.

## Discussion

### Overall Effects of Consumer-Wearable Activity Tracker-Based Programs

The present systematic review and meta-analysis synthesizes the evidence to date about the effectiveness of consumer-wearable activity tracker-based programs on daily objectively measured PA and SB among apparently healthy school-aged children [20, 21, 43–45, 52–55, 60–94]. The overall results showed that the consumer-wearable activity tracker-based programs brought about significant moderate improvements in school-aged children’s daily total steps, and small but significant improvements in daily MVPA levels after the intervention program. However, regarding daily levels of total PA the effect was trivial. Therefore, the use of a consumer-wearable activity tracker as a motivation tool for young people is strongly recommended to reduce the high levels of physical inactivity among school-aged children [8]. However, the intervention programs seem not to reduce the school-aged children’s SB.

These results agree with similar previous meta-analyses carried out in adults [24–26]. Firstly, regarding daily total steps, all previous reviews found improvements, although they seem greater in the present systematic review (*d* = 0.612 vs. 0.240–0.449; *D* = 1,692.79 vs. 950.54). Regarding changes in actual units, adding 1,000 steps per day, which is equivalent to a 10% increase within the recommended 10,000 steps per day [19, 95], has been shown to be a significant and clinically meaningful change related to a substantial reduction of the risk of all-cause mortality in the adult population [96, 97]. Therefore, although there is no such evidence in studies carried out with school-aged children, the increase of 1,692.79 daily steps obtained in the present meta-analysis might be considered an important and meaningful change considering the existing evidence with adults. Moreover, with reference to MVPA, similar improvements in daily levels of MVPA were obtained (*d* = 0.220 vs. 0.270; *D* = 5.583 vs. 6.160). With reference to changes in actual units, to our knowledge, there is no clinical evidence of a link between changes in minutes involved in MVPA and health outcomes in any population. However, extrapolating the results obtained as a percentage of the international recommendations (i.e., 60 min of MVPA per day [4]), the obtained results (i.e., 5.6 min) represent almost a 10% increase. Therefore, considering the same reasoning explained above with daily total steps, these changes in daily levels of MVPA may also be considered clinically significant.

The greater improvement in school-aged children’s daily total steps than in their MVPA levels may be due to the kind of goal established in the program. Most of the studies included in the systematic review with a goal-setting strategy set only a step-based goal (30 of 37 studies), while only five studies established both step-based and minutes of total PA-based goals [62, 87] or SB-based goals [88, 91]. Therefore, all the motivational strategies included, such as reminders [44, 54, 66], counseling sessions [70], or rewards [65, 73] were carried out around this goal of increasing the number of steps. Moreover, the reason for relying mainly on the number of steps as the reference output for goal-setting may be due to steps having the advantage of being easier to understand and interpret by school-aged children compared to MVPA minutes [19]. In addition, in many studies, the consumer-wearable activity-tracker used were pedometers, which only show feedback about the number of steps. Therefore, similar to the evidence found with adults [24–26], consumer-wearable activity tracker-based programs seem to be effective for improving objectively measured daily PA, especially for daily total steps, although the effects seem to be greater for school-aged children. This greater effect may be due to the fact that school-aged children have a higher affinity with new technologies playing an important role in their daily life, and therefore these kinds of technology-based interventions may be more interesting for them [98, 99]. In addition, during the period of childhood and early adolescence, school-aged children are still forming their daily habits and they are more sensitive to changing their PA behavior which could explain these greater intervention effects, while in adulthood the stability of PA patterns is high and more difficult to be changed [100]

Regarding total PA, results showed a trivial favorable effect (*d* = 0.151, 95% CI 0.038–0.264). These results are curious because daily total steps is an indicator of total PA [101], and therefore, results were expected to be similar. However, it may be because very slow steps were included in daily total steps, but they do not reach the threshold to be considered light PA, or because fewer studies are assessing total PA compared to studies assessing daily total steps. Nevertheless, in the present meta-analysis, only the study by Corepal et al. [65] measured both variables in the same study, without any clear relationship between both outputs. Therefore, future studies including the evaluation of both variables (i.e., daily total steps and total PA) would be very interesting to establish a real cause–effect relationship between them.

Besides, regarding SB, similar to the trivial unfavorable effect obtained in the present systematic review (*d* = 0.172; 95% CI 0.039–0.305), no real differences were found in any previous review [24, 26]. Firstly, this may be due to the fact that most of the programs that evaluate SB only used strategies to encourage and support PA behavior change (i.e., goals, tips and challenges, behavioral incentives or reinforcement messages based only on PA practice) and were not specifically designed to reduce SB [21, 43, 62, 65, 77, 85]. Only Leinonen et al. [53] specifically included some SB-based strategies, such as feedback showing a thumb either up or down if the day included over two hours of sedentary (sitting) periods or not, and rewards regarding decrement in weekly school-aged children’s sedentary time. The lack of specific focus on reducing SB may have contributed to this trivial unfavorable effect. Moreover, these results may also present significant measurement bias due to all SB-studies (except Morris et al. [85]) analyzed raw time involved in SB per day instead of valid wear time-based standardized scores (e.g., percentage of time of each day engaged in SB of the total valid wear time; standardized mean SB in minutes) [102, 103]. In addition, it must be considered that for accelerometer-based measures only a valid minimum time per day is established (normally 600 min), but that standardized values are not taken into account [104]. This is even more accentuated for consumer-based wearables for which valid wear time cannot be known and, therefore, it cannot be controlled if school-aged children wore the wearable for a long enough time, or if they were more motivated to wear it for more time in the baseline or post-intervention measure. In this sense, it is easier for school-aged children with more valid time to have higher registered time involved in SB, so SB time outcomes could be especially affected by potential systematic valid wear-time variation between measurement moments (i.e., pre–post-intervention measures) or groups [102]. This could also affect daily total steps and daily MVPA levels although to a much lesser extent than for SB [102]. For instance, Gaudet et al. [43] showed that school-aged children in the experimental group had approximately 48 min more wear time per day in comparison with the control group, which may directly affect their differences regarding time involved in SB. However, most studies [21, 62, 65, 77] only reported the minimum time per day needed to be included in the study but did not report the actual mean valid wear time per day, and it is important that this be reported in order to compare compliance between groups. Finally, it should be noted that there are very few studies measuring school-aged children’s SB in comparison with studies measuring steps and MVPA, which implies a wider confidence interval and consequently greater uncertainty about the real value.

### Influence of Participants’ Characteristics

According to the results of the present meta-analysis, a significant relationship has been observed for PA status from the within-study subgroups analysis, the intervention being much more effective for improving daily total steps in school-aged children who were physically inactive in the baseline measure than in those who were physically active. Furthermore, this positive influence was also found for improving objectively measured daily MVPA levels, although only from the between-study analysis (i.e., an observational relationship). A potential reason for this influence could be that school-aged children who already are physically active before the intervention are motivated enough for PA practice without the need for extra motivation with the proposed intervention [105]. Furthermore, it is difficult to further increase school-aged children’s PA levels when the baseline levels are high, just as has occurred with the improvement of physical fitness levels [106, 107]. Therefore, consumer-wearable activity tracker-based programs may be an especially appropriate strategy for less active school-aged children [82, 85]. In line with the recommendation by Love et al.'s [108] systematic review, future studies should analyze their results distinguishing by school-aged children’s baseline PA profiles to correctly identify the intervention impact because all participants do not react in the same way to the intervention. Regarding school-aged children who already are physically active before the intervention, it may be necessary to study which specific strategies should be implemented in these interventions to help them maintain their PA levels, or even to continue increasing them, therefore obtaining greater health-related benefits [4]. Finally, regarding school-aged children’s age, it does not seem to affect the intervention program effect.

Furthermore, programs seem to be more effective in females than in males for improving daily total steps, which could be related to PA status since females tend to be more physically inactive than males throughout childhood and adolescence [109, 110]. Therefore, sex-specific interventions could be considered in future research like Böhm et al. [29] suggested, although the conclusions in relation to the effectiveness of sex-specific interventions should be taken with caution because the success of those programs was not obtained by the within-study subgroups analysis (i.e., cause–effect relationships), but rather by the between-study analysis, which only establishes observational relationships [20, 44, 69, 73, 74, 83]. Furthermore, the between-study subgroups analysis results still showed moderate-to-high heterogeneity in each subgroup (i.e., males and females), so it is likely that there were differences in other strategies of the intervention or participants’ characteristics that could influence its effect, even more than sex itself. Additionally, these differences are even more accentuated because the analysis includes twice as many publications carried out with females than with males. However, the number of studies including this within-study sex comparison and which allow establishing cause–effect relationships is very limited (*k* = 7), and for this reason, the observational relationships of the between-study comparisons have been considered in the present meta-analysis.

### Influence of Intervention Programs’ Characteristics

Firstly, overall meta-regression analysis results showed a higher effect for daily total steps when a greater number of strategies were included in the programs. Therefore, multi-dimensional interventions that include most of these strategies seem to be preferable for mediating PA behavior. That agrees with some psychological theories which include most of those strategies as positive mediators influencing PA behavior. For instance, the Social Cognitive Theory [111], highlighted PA-related knowledge included in counseling sessions, positive reinforcement such as reminders with encouraging messages about PA practice (e.g., “You’re in charge! Make the choice to meet your step goal today!”), and the importance of setting achievable goals as determining factors in the design of PA promotion interventions that could lead to behavior change in school-aged children. In addition, the Self-Determination Theory [112], emphasizes the need for relatedness included in most motivational strategies (e.g., teamwork or the use of social networks), and the perceived competence reflected in the reinforcement reminders praising their efforts (e.g., “You can meet your step goal; just keep stepping!”), or the evolution that school-aged children could observe in the diary they filled in during the program, as necessary to increase their intrinsic motivation. Furthermore, recent systematic reviews highlight that using multi-strategy approaches as behavior change techniques show better PA outcomes than singular change approaches [113, 114]. For instance, the PA-related knowledge provided in the counseling sessions, the autonomy support environment by the consumer-wearable activity tracker feedback, the inclusion of additional motivational strategies like social networks, setting goals of moderate difficulty, or sending reminders with encouraging messages about PA practice have been shown to positively change school-aged children’s PA behavior [115, 116]. However, it should be noted that the explained variance was low (*R*^2^ = 0.02), as well as the results being highly heterogeneous (*I*^2^ = 89.02).

Furthermore, there was considerable heterogeneity in the strategies included in the reviewed studies. Most of the programs used a goal-setting strategy, participants’ logbooks, educational counseling sessions, and/or some kind of motivational strategy. Nevertheless, the inclusion of reminders to persuade participants to move or exercise more was a less frequently included strategy. The influence of these intervention program characteristics in school-aged children’s daily total steps and MVPA has been analyzed in the present meta-analysis. Firstly, interventions including some kind of counseling [54, 60, 71] and/or goal-setting techniques [73, 86, 90], in addition to consumer-wearable activity trackers, were highlighted as more effective than those without them for improving school-aged children’s daily total steps. These results are in accordance with previous studies’ recommendations about the inclusion of these explicit strategies (e.g., advice about PA benefits, strategies to reduce SB and increase PA, resolution of barriers to PA practice, or goal-setting strategies based on the international guidelines) which make students feel that they are making an informed decision about their health in any kind of program for PA promotion [11, 13] and specifically in wearable-based programs [27, 115]. Nevertheless, apparently contradictory results showed that consumer-wearable activity trackers-based programs which did not include any exercise routine seem to be more effective than those that included it for improving school-aged children’s daily MVPA levels. However, analyzing the kind of exercise routine included, had some limitations. Firstly, Jago et al. [62] and Smith et al. [91] included a low frequency of supervised PA sessions (one 20-min PA session per week, and only six 20-min lessons in 20 weeks, respectively) with which it is very difficult to positively affect the school-aged children’s daily PA levels. Secondly, most of the activities included by Jago et al. [62] did not have a direct relationship with increasing the school-aged children’s number of steps or minutes involved in MVPA (i.e., stretching, technical drills, or strengthening tasks), and also it should be noted that despite including this exercise routine, they did not include other strategies that may be even more important than this one (e.g., reminders, diary, or motivational strategies). Finally, it is also important to denote that the analysis included 17 units of analysis without an exercise routine vs. three with an exercise routine [62, 91], so given this marked difference in the sample, results should be interpreted with caution. Moreover, it must be considered that only observational relationships have been obtained from the between-study subgroups analyses due to the low number of studies compared in the within-study subgroups analyses. Furthermore, the results regarding school-aged children’s daily total steps separated by each subgroup still showed a high level of heterogeneity which implies differences in other intervention characteristics. Therefore, future studies should include different intervention groups that compared some intervention characteristics (e.g., one experimental group including counseling and another without counseling) to establish causal-effect relationships between intervention characteristics and the effect of the programs.

Regarding the kind of consumer-wearable activity tracker, waist-worn trackers such as pedometers are more common than wrist-worn trackers. This could mainly be due to the fact that waist-worn trackers such as traditional step counters with digital displays, have featured in scientific research since approximately 1996, being an accepted method for assessing PA and a tool for walking interventions [117]. On the contrary, wrist-worn trackers have burst onto the market in the last decade and, therefore, their scientific evidence base is still scarce [14, 18]. Moreover, the meta-analysis results showed that programs carried out with a wrist-worn activity tracker seem to be more effective than those carried out with waist-worn trackers for improving school-aged children’s daily MVPA levels. This may be due to wrist-worn trackers having several advantages compared to waist-worn trackers, such as reporting real-time feedback that can be easily checked on their wrist or touch screens [14]. Moreover, unlike more traditional waist-based trackers, which only monitor and display simple feedback about PA levels, wrist-based trackers are much more interactive since the user is able to set reminders, notifications, or congratulatory messages upon reaching the proposed goal [118]. Finally, wrist devices have shown greater wear time compliance which could mean that if they wear it for a longer time school-aged children could interact more with its features [119].

### Risk of Bias and Certainty of the Evidence

Firstly, based on the methodological risk of bias assessment, most studies were classified as “high risk” or “some concerns,” leaving only two studies classified as overall “low risk” [21, 81]. Therefore, this may have resulted in a biased assessment of the intervention effect, underestimating or overestimating the true intervention effect, which meant downgrading the GRADE certainty rating by one level for all outcomes (i.e., daily total steps, MVPA, total PA, and SB) regarding the risk of bias domain [34, 46]. In reference to the study designs, it is interesting to highlight that only 51.11% of the included studies are true or cluster-randomized controlled trials, which are markedly far stronger interventions to demonstrate effect significance [120]. However, sensitivity analysis showed no differences for school-aged children’s daily MVPA, total PA, and SB levels between study designs, but much greater effects were found in randomized controlled trials for school-aged children’s daily total steps than non-randomized trials.

Moreover, regarding daily total steps outcomes, a substantial level of heterogeneity was found, even in the follow-up subgroups analyses (except when separating by accomplishment of PA recommendations) and it meant downgrading the GRADE certainty rating by another level regarding inconsistency domain for daily total steps. This is most likely because it is the PA outcome that includes the largest number of studies and, consequently, the greatest variety in the types of intervention applied when compared with other outcome measures. Finally, regarding publication bias, although the funnel plots and Egger’s test suggested publication bias for daily total steps and daily MVPA levels, its impact seems to be very low given the unlikely number of “lost” studies suggested by the fail-safe *N* analyses. Furthermore, the Trim and Fill method did not trim any study for daily steps and only two studies for MVPA, resulting in an adjusted value similar to the observed values (*d* = 0.220 vs. 0.213).

For all the above-mentioned reasons, it is important to highlight the “low” certainty of evidence found for daily total steps, which means that the confidence in the effect estimate is limited and the true effect may be different from the estimated effect [121]. Regarding MVPA, total PA and SB outcomes, “moderate” certainty of evidence was found, so the true effect is likely close to the estimated effect, but there is a possibility that it is substantially different [121]. Therefore, the findings of the present meta-analysis should be considered with caution and firmer conclusions should await the accumulation of a larger high-quality number of primary studies.

### Strengths and Limitations

Regarding the strengths of the present systematic review, numerous measures to avoid, or at least to reduce, publication bias were followed (e.g., the inclusion of a great range of bibliographic databases from different disciplines and complementary search strategies, or not restricting the search by the language, type or date of publication). Then, several exploratory analyses were conducted to identify and assess the impact of any potential publication bias (e.g., funnel plots, or Orwin’s fail-safe N analyses), as well as sensitivity analyses (e.g., Hedges’ *g* with a random-effects model or Cohen’s *d* with a random-effects model separately for randomized controlled trial design or not) to verify the robustness of the results. Furthermore, the present review was focused only on objective measurements, which have shown high validity to measure PA and SB levels in comparisons with self-reported measures [35, 36]. Lastly, to our knowledge, to date this is the first systematic review and meta-analysis about the effects of consumer-based activity tracker-based programs on objectively measured PA and SB levels within apparently healthy school-aged children, including analyzing the influence of the intervention programs’ characteristics and school-aged children’s characteristics on the effects. This meta-analysis summarizes the effectiveness of those interventions in an overall statistical synthesis, improving the precision of the results by the estimation of the effect size and direction, and clarifying whether or not the effect size is consistent across studies.

However, the present systematic review and meta-analysis is not without limitations. First, although randomized controlled trials have higher methodological quality, the present systematic review includes several study designs. As expected, there were not many consumer-based activity tracker-based studies with a high level of quality design for improving the different PA-related behaviors. Therefore, a reason for including several designs is to provide evidence of the effects of interventions for which only a small number of randomized controlled trials are available, drawing on the “best available evidence” rather than the “highest tier” of evidence [34]. Nevertheless, sensitivity analyses were also performed comparing randomized controlled trials and non-randomized trials, showing no differences for school-aged children’s daily MVPA, total PA, and SB levels. Furthermore, even greater effects in school-aged children’s daily total steps were found in randomized controlled trials. Second, although the inclusion of a wide range of intervention types, populations, sample size, and study designs had some advantages regarding the generalizability of conclusions, it also means a high level of heterogeneity. For instance within intervention types, multiple behavior change strategies such as goal-setting (even including different kinds like static or adaptive goals) or extra motivational strategies (e.g., social networks or social support) were usually combined in the same study. Therefore, it makes the independent contribution of any intervention features and, therefore, making strong conclusions from the intervention difficult to establish. However, in addition to the overall effect size, subgroups analyses and meta-regression of the a priori hypothesized moderators were also performed. Therefore, not only general effect results are provided, but also results for each specific group based on the characteristics of the programs and school-aged children. Third, the present systematic review investigated effectiveness at the end of the program (i.e., short-term), but future studies should investigate long-term effectiveness to assess actual behavioral changes some months after the program. However, due to the very limited evidence, this was not performed in the present systematic review. Finally, coding some study outcomes was problematic due to authors not reporting them. Although authors were contacted, many of them did not reply and the particular study outcome had to be omitted. However, this is a common problem in most systematic reviews [34], and a great effort was made in contacting authors, recalculating data, or estimating values from figures. Finally, in some cases, consumer-based activity trackers were used both as a motivational instrument during the intervention and to objectively measure PA, which could affect results by increasing their actual PA levels in the control group or during baseline assessments.

## Conclusions

The present findings suggest that consumer-wearable activity tracker-based programs within school-aged children have a statistically significant moderate favorable effect on daily total steps and a small, but favorable effect on objectively measured daily levels of MVPA. However, the favorable effect of the programs on school-aged children’s objectively measured total PA levels and the unfavorable effect in SB was trivial, although statistically significant. The findings of this systematic review suggest that programs are more effective in females for increasing daily total steps, and in physically inactive subjects for increasing both daily total steps and daily MVPA levels. Moreover, the inclusion of a greater number of strategies in the programs had a higher effect on school-aged children’s daily total steps. It should be highlighted that prompting specific goal-setting and the inclusion of educational counseling sessions are particularly useful strategies to include in consumer-wearable activity tracker-based programs designed to promote school-aged children’s daily total steps. Furthermore, regarding the kind of consumer-wearable activity tracker, programs were more effective at increasing school-aged children’s daily MVPA levels if a wrist-worn activity tracker was used. However, since only observational relationships have been obtained from the between-study subgroups analyses due to the low number of studies, all the above-mentioned recommendations regarding intervention strategies should be taken with caution. However, due to the certainty of evidence being from “low” to “moderate,” further primary research is needed to determine the effectiveness of these programs using robust designs with low risk of bias, and which compare the effect of different intervention characteristics in the same study. Consumer-wearable activity tracker-based programs (particularly those including goal-setting, educational counseling, and wrist-worn trackers) seem to be effective for promoting school-aged children’s daily total steps and MVPA, especially for females and those who are physically inactive.

## Supplementary Information

**Additional file 1**. Prisma checklist.

**Additional file 2**. Search strategies.

**Additional file 3**. Coding form followed in the present systematic review.

**Additional file 4**. Algorithms followed for assessing methodological risk-of-bias in each domain.

**Additional file 5**. Risk of bias assessment: (a) Daily steps units of analysis; (b) Moderate-to-vigorous physical activity units of analysis; (c) Total physical activity unit of analysis; and (d) Sedentary behavior unit of analysis. Each row corresponds to a unit of analysis. Green symbols represent a “Low risk of bias”, yellow symbols represent “Some concerns”, and red symbols represent a “High risk of bias”.

**Additional file 6**. Certainty of the evidence assessment.

**Additional file 7**. Results of the cumulative meta-analyses by study size for: (a) Daily total steps; and (b) Moderate-to-vigorous physical activity.

**Additional file 8**. Results of the within-study subgroups analyses for the effect of the consumer-wearable activity tracker-based programs on the daily total steps among school-aged children.

**Additional file 9**. Results of the between-study subgroups analyses for the effect of the consumer-wearable activity tracker-based programs on the daily total steps among school-aged children.

**Additional file 10**. Results of the within-study subgroups analyses for the effect of the consumer-wearable activity tracker-based programs on the daily moderate-to-vigorous physical activity among school-aged children.

**Additional file 11**. Results of the between-study subgroups analyses for the effect of the consumer-wearable activity tracker-based programs on the daily moderate-to-vigorous physical activity among school-aged children.

## Data Availability

The dataset and code used for meta-analysis are available from the corresponding author [DMV] on request.

## Abbreviations

PA: Physical activity
SB: Sedentary behavior
MVPA: Moderate-to-vigorous physical activity
WHO: World Health Organization
