# Supplementary material for: Effects of Consumer-Wearable Activity Tracker-Based Programs on Objectively Measured Daily Physical Activity and Sedentary Behavior Among School-Aged Children: A Systematic Review and Meta-analysis
Source: Sports Med Open. 2022 Jan 31;8:18. doi: 10.1186/s40798-021-00407-6 (PMC8804065; doi:10.1186/s40798-021-00407-6)
Supplement: Supplementary file 2 — Additional file 2. Search strategies. [file 40798_2021_407_MOESM2_ESM.docx]

Supplementary File 2. Search strategies

1. **WEB OF SCIENCE (All Databases)**

TS=("self-tracker-based" OR "self-tracker-supported" OR "fitness self-trackers" OR "activity self-trackers" OR "wearable self-trackers" OR "fitness self-tracker" OR "activity self-tracker" OR "wearable self-tracker" OR "fitness self-tracking" OR "activity self-tracking" OR "wearable self-tracking" OR "step self-trackers" OR "step self-tracker" OR "step self-tracking" OR "steps self-trackers" OR "steps self-tracker" OR "steps self-tracking" OR "step-based self-trackers" OR "step-based self-tracker" OR "step-based self-tracking" OR "step count self-trackers" OR "step count self-tracker" OR "step count self-tracking" OR "step counts self-trackers" OR "step counts self-tracker" OR "step counts self-tracking" OR "step counter self-trackers" OR "step counter self-tracker" OR "step counter self-tracking" OR "step counting self-trackers" OR "step counting self-tracker" OR "step counting self-tracking" OR "steps count self-trackers" OR "steps count self-tracker" OR "steps count self-tracking" OR "steps counts self-trackers" OR "steps counts self-tracker" OR "steps counts self-tracking" OR "steps counter self-trackers" OR "steps counter self-tracker" OR "steps counter self-tracking" OR "steps counting self-trackers" OR "steps counting self-tracker" OR "steps counting self-tracking" OR "wrist-based self-trackers" OR "wrist-based self-tracker" OR "wrist-based self-tracking" OR "wrist-worn self-trackers" OR "wrist-worn self-tracker" OR "wrist-worn self-tracking" OR "arm-based self-trackers" OR "arm-based self-tracker" OR "arm-based self-tracking" OR "arm-worn self-trackers" OR "arm-worn self-tracker" OR "arm-worn self-tracking" OR "hip-based self-trackers" OR "hip-based self-tracker" OR "hip-based self-tracking" OR "hip-worn self-trackers" OR "hip-worn self-tracker" OR "hip-worn self-tracking" OR "wearable system" OR "wearable systems" OR "pedometer-based" OR "tracker-based" OR "wearable-based" OR "technology-based" OR "pedometer-supported" OR "tracker-supported" OR "wearable-supported" OR "technology-supported" OR "fitness sensors" OR "activity sensors" OR "wearable sensors" OR "fitness sensor" OR "activity sensor" OR "wearable sensor" OR "step sensors" OR "step sensor" OR "steps sensors" OR "steps sensor" OR "step-based sensors" OR "step-based sensor" OR "step count sensors" OR "step count sensor" OR "step counts sensors" OR "step counts sensor" OR "step counter sensors" OR "step counter sensor" OR "step counting sensors" OR "step counting sensor" OR "steps count sensors" OR "steps count sensor" OR "steps counts sensors" OR "steps counts sensor" OR "steps counter sensors" OR "steps counter sensor" OR "steps counting sensors" OR "steps counting sensor" OR "wrist-based sensors" OR "wrist-based sensor" OR "wrist-worn sensors" OR "wrist-worn sensor" OR "arm-based sensors" OR "arm-based sensor" OR "arm-worn sensors" OR "arm-worn sensor" OR "hip-based sensors" OR "hip-based sensor" OR "hip-worn sensors" OR "hip-worn sensor" OR "wearable technology" OR "fitness trackers" OR "activity trackers" OR "wearable trackers" OR "fitness tracker" OR "activity tracker" OR "wearable tracker" OR "fitness tracking" OR "activity tracking" OR "wearable tracking" OR "fitness wearables" OR "activity wearables" OR "fitness wearable" OR "activity wearable" OR "fitness devices" OR "activity devices" OR "wearable devices" OR "fitness device" OR "activity device" OR "wearable device" OR "fitness monitors" OR "activity monitors" OR "wearable monitors" OR "fitness monitor" OR "activity monitor" OR "wearable monitor" OR "fitness monitoring" OR "activity monitoring" OR "wearable monitoring" OR pedometers OR pedometer OR "step trackers" OR "step tracker" OR "step tracking" OR "step devices" OR "step device" OR "step monitors" OR "step monitor" OR "step monitoring" OR "steps trackers" OR "steps tracker" OR "steps tracking" OR "steps devices" OR "steps device" OR "steps monitors" OR "steps monitor" OR "steps monitoring" OR "step-based trackers" OR "step-based tracker" OR "step-based tracking" OR "step-based devices" OR "step-based device" OR "step-based monitors" OR "step-based monitor" OR "step-based monitoring" OR "step count trackers" OR "step count tracker" OR "step count tracking" OR "step count devices" OR "step count device" OR "step count monitors" OR "step count monitor" OR "step count monitoring" OR "step counts trackers" OR "step counts tracker" OR "step counts tracking" OR "step counts devices" OR "step counts device" OR "step counts monitors" OR "step counts monitor" OR "step counts monitoring" OR "step counter trackers" OR "step counter tracker" OR "step counter tracking" OR "step counter devices" OR "step counter device" OR "step counter monitors" OR "step counter monitor" OR "step counter monitoring" OR "step counting trackers" OR "step counting tracker" OR "step counting tracking" OR "step counting devices" OR "step counting device" OR "step counting monitors" OR "step counting monitor" OR "step counting monitoring" OR "steps count trackers" OR "steps count tracker" OR "steps count tracking" OR "steps count devices" OR "steps count device" OR "steps count monitors" OR "steps count monitor" OR "steps count monitoring" OR "steps counts trackers" OR "steps counts tracker" OR "steps counts tracking" OR "steps counts devices" OR "steps counts device" OR "steps counts monitors" OR "steps counts monitor" OR "steps counts monitoring" OR "steps counter trackers" OR "steps counter tracker" OR "steps counter tracking" OR "steps counter devices" OR "steps counter device" OR "steps counter monitors" OR "steps counter monitor" OR "steps counter monitoring" OR "steps counting trackers" OR "steps counting tracker" OR "steps counting tracking" OR "steps counting devices" OR "steps counting device" OR "steps counting monitors" OR "steps counting monitor" OR "steps counting monitoring" OR "fitness wristband" OR "activity wristband" OR "step wristband" OR "steps wristband" OR "step-based wristband" OR "step count wristband" OR "step counts wristband" OR "step counter wristband" OR "step counting wristband" OR "steps count wristband" OR "steps counts wristband" OR "steps counter wristband" OR "steps counting wristband" OR "fitness wristbands" OR "activity wristbands" OR "step wristbands" OR "steps wristbands" OR "step-based wristbands" OR "step count wristbands" OR "step counts wristbands" OR "step counter wristbands" OR "step counting wristbands" OR "steps count wristbands" OR "steps counts wristbands" OR "steps counter wristbands" OR "steps counting wristbands" OR "fitness armband" OR "activity armband" OR "step armband" OR "steps armband" OR "step-based armband" OR "step count armband" OR "step counts armband" OR "step counter armband" OR "step counting armband" OR "steps count armband" OR "steps counts armband" OR "steps counter armband" OR "steps counting armband" OR "fitness armbands" OR "activity armbands" OR "step armbands" OR "steps armbands" OR "step-based armbands" OR "step count armbands" OR "step counts armbands" OR "step counter armbands" OR "step counting armbands" OR "steps count armbands" OR "steps counts armbands" OR "steps counter armbands" OR "steps counting armbands" OR "fitness bracelet" OR "activity bracelet" OR "step bracelet" OR "steps bracelet" OR "step-based bracelet" OR "step count bracelet" OR "step counts bracelet" OR "step counter bracelet" OR "step counting bracelet" OR "steps count bracelet" OR "steps counts bracelet" OR "steps counter bracelet" OR "steps counting bracelet" OR "fitness bracelets" OR "activity bracelets" OR "step bracelets" OR "steps bracelets" OR "step-based bracelets" OR "step count bracelets" OR "step counts bracelets" OR "step counter bracelets" OR "step counting bracelets" OR "steps count bracelets" OR "steps counts bracelets" OR "steps counter bracelets" OR "steps counting bracelets" OR "fitness band" OR "activity band" OR "step band" OR "steps band" OR "step-based band" OR "step count band" OR "step counts band" OR "step counter band" OR "step counting band" OR "steps count band" OR "steps counts band" OR "steps counter band" OR "steps counting band" OR "fitness bands" OR "activity bands" OR "step bands" OR "steps bands" OR "step-based bands" OR "step count bands" OR "step counts bands" OR "step counter bands" OR "step counting bands" OR "steps count bands" OR "steps counts bands" OR "steps counter bands" OR "steps counting bands" OR "wrist-based trackers" OR "wrist-based tracker" OR "wrist-based tracking" OR "wrist-based wearables" OR "wrist-based wearable" OR "wrist-based devices" OR "wrist-based device" OR "wrist-based monitors" OR "wrist-based monitor" OR "wrist-based monitoring" OR "wrist-based fitness" OR "wrist-based activity" OR "wrist-worn trackers" OR "wrist-worn tracker" OR "wrist-worn tracking" OR "wrist-worn wearables" OR "wrist-worn wearable" OR "wrist-worn devices" OR "wrist-worn device" OR "wrist-worn monitors" OR "wrist-worn monitor" OR "wrist-worn monitoring" OR "wrist-worn fitness" OR "wrist-worn activity" OR "arm-based trackers" OR "arm-based tracker" OR "arm-based tracking" OR "arm-based wearables" OR "arm-based wearable" OR "arm-based devices" OR "arm-based device" OR "arm-based monitors" OR "arm-based monitor" OR "arm-based monitoring" OR "arm-based fitness" OR "arm-based activity" OR "arm-worn trackers" OR "arm-worn tracker" OR "arm-worn tracking" OR "arm-worn wearables" OR "arm-worn wearable" OR "arm-worn devices" OR "arm-worn device" OR "arm-worn monitors" OR "arm-worn monitor" OR "arm-worn monitoring" OR "arm-worn fitness" OR "arm-worn activity" OR "hip-based trackers" OR "hip-based tracker" OR "hip-based tracking" OR "hip-based wearables" OR "hip-based wearable" OR "hip-based devices" OR "hip-based device" OR "hip-based monitors" OR "hip-based monitor" OR "hip-based monitoring" OR "hip-based fitness" OR "hip-based activity" OR "hip-worn trackers" OR "hip-worn tracker" OR "hip-worn tracking" OR "hip-worn wearables" OR "hip-worn wearable" OR "hip-worn devices" OR "hip-worn device" OR "hip-worn monitors" OR "hip-worn monitor" OR "hip-worn monitoring" OR "hip-worn fitness" OR "hip-worn activity" OR smartwatches OR smartwatch OR Fitbit OR Jawbone OR Samsung OR Xiaomi OR Garmin OR "Polar Loop" OR "Polar A300" OR "Polar A360" OR "Polar A370" OR Sony OR Huawei OR Misfit OR "LG Lifeband" OR "LG watch" OR "LG Gwatch" OR "LG G watch" OR Nike OR "Apple watch" OR Suunto) AND TS=(program OR programs OR programme OR programmes OR training OR trainings OR intervention OR interventions OR treatment OR treatments OR "teaching unit" OR "teaching units" OR school OR schools OR "school-based" OR "physical education" OR PE OR "P.E." OR "physical education-based") AND TS=("physical activity" OR "physical activities" OR exercise OR sedentary OR sedentarism OR steps OR step OR energy OR kcals OR kcal OR calories OR calorie OR kilocalories OR kilocalorie OR MET OR METS OR "metabolic equivalent" OR MVPA OR "vertical axis" OR "vector magnitude" OR counts)

1. **SCOPUS**

TITLE-ABS-KEY("self-tracker-based" OR "self-tracker-supported" OR "fitness self-trackers" OR "activity self-trackers" OR "wearable self-trackers" OR "fitness self-tracker" OR "activity self-tracker" OR "wearable self-tracker" OR "fitness self-tracking" OR "activity self-tracking" OR "wearable self-tracking" OR "step self-trackers" OR "step self-tracker" OR "step self-tracking" OR "steps self-trackers" OR "steps self-tracker" OR "steps self-tracking" OR "step-based self-trackers" OR "step-based self-tracker" OR "step-based self-tracking" OR "step count self-trackers" OR "step count self-tracker" OR "step count self-tracking" OR "step counts self-trackers" OR "step counts self-tracker" OR "step counts self-tracking" OR "step counter self-trackers" OR "step counter self-tracker" OR "step counter self-tracking" OR "step counting self-trackers" OR "step counting self-tracker" OR "step counting self-tracking" OR "steps count self-trackers" OR "steps count self-tracker" OR "steps count self-tracking" OR "steps counts self-trackers" OR "steps counts self-tracker" OR "steps counts self-tracking" OR "steps counter self-trackers" OR "steps counter self-tracker" OR "steps counter self-tracking" OR "steps counting self-trackers" OR "steps counting self-tracker" OR "steps counting self-tracking" OR "wrist-based self-trackers" OR "wrist-based self-tracker" OR "wrist-based self-tracking" OR "wrist-worn self-trackers" OR "wrist-worn self-tracker" OR "wrist-worn self-tracking" OR "arm-based self-trackers" OR "arm-based self-tracker" OR "arm-based self-tracking" OR "arm-worn self-trackers" OR "arm-worn self-tracker" OR "arm-worn self-tracking" OR "hip-based self-trackers" OR "hip-based self-tracker" OR "hip-based self-tracking" OR "hip-worn self-trackers" OR "hip-worn self-tracker" OR "hip-worn self-tracking" OR "wearable system" OR "wearable systems" OR "pedometer-based" OR "tracker-based" OR "wearable-based" OR "technology-based" OR "pedometer-supported" OR "tracker-supported" OR "wearable-supported" OR "technology-supported" OR "fitness sensors" OR "activity sensors" OR "wearable sensors" OR "fitness sensor" OR "activity sensor" OR "wearable sensor" OR "step sensors" OR "step sensor" OR "steps sensors" OR "steps sensor" OR "step-based sensors" OR "step-based sensor" OR "step count sensors" OR "step count sensor" OR "step counts sensors" OR "step counts sensor" OR "step counter sensors" OR "step counter sensor" OR "step counting sensors" OR "step counting sensor" OR "steps count sensors" OR "steps count sensor" OR "steps counts sensors" OR "steps counts sensor" OR "steps counter sensors" OR "steps counter sensor" OR "steps counting sensors" OR "steps counting sensor" OR "wrist-based sensors" OR "wrist-based sensor" OR "wrist-worn sensors" OR "wrist-worn sensor" OR "arm-based sensors" OR "arm-based sensor" OR "arm-worn sensors" OR "arm-worn sensor" OR "hip-based sensors" OR "hip-based sensor" OR "hip-worn sensors" OR "hip-worn sensor" OR "wearable technology" OR "fitness trackers" OR "activity trackers" OR "wearable trackers" OR "fitness tracker" OR "activity tracker" OR "wearable tracker" OR "fitness tracking" OR "activity tracking" OR "wearable tracking" OR "fitness wearables" OR "activity wearables" OR "fitness wearable" OR "activity wearable" OR "fitness devices" OR "activity devices" OR "wearable devices" OR "fitness device" OR "activity device" OR "wearable device" OR "fitness monitors" OR "activity monitors" OR "wearable monitors" OR "fitness monitor" OR "activity monitor" OR "wearable monitor" OR "fitness monitoring" OR "activity monitoring" OR "wearable monitoring" OR pedometers OR pedometer OR "step trackers" OR "step tracker" OR "step tracking" OR "step devices" OR "step device" OR "step monitors" OR "step monitor" OR "step monitoring" OR "steps trackers" OR "steps tracker" OR "steps tracking" OR "steps devices" OR "steps device" OR "steps monitors" OR "steps monitor" OR "steps monitoring" OR "step-based trackers" OR "step-based tracker" OR "step-based tracking" OR "step-based devices" OR "step-based device" OR "step-based monitors" OR "step-based monitor" OR "step-based monitoring" OR "step count trackers" OR "step count tracker" OR "step count tracking" OR "step count devices" OR "step count device" OR "step count monitors" OR "step count monitor" OR "step count monitoring" OR "step counts trackers" OR "step counts tracker" OR "step counts tracking" OR "step counts devices" OR "step counts device" OR "step counts monitors" OR "step counts monitor" OR "step counts monitoring" OR "step counter trackers" OR "step counter tracker" OR "step counter tracking" OR "step counter devices" OR "step counter device" OR "step counter monitors" OR "step counter monitor" OR "step counter monitoring" OR "step counting trackers" OR "step counting tracker" OR "step counting tracking" OR "step counting devices" OR "step counting device" OR "step counting monitors" OR "step counting monitor" OR "step counting monitoring" OR "steps count trackers" OR "steps count tracker" OR "steps count tracking" OR "steps count devices" OR "steps count device" OR "steps count monitors" OR "steps count monitor" OR "steps count monitoring" OR "steps counts trackers" OR "steps counts tracker" OR "steps counts tracking" OR "steps counts devices" OR "steps counts device" OR "steps counts monitors" OR "steps counts monitor" OR "steps counts monitoring" OR "steps counter trackers" OR "steps counter tracker" OR "steps counter tracking" OR "steps counter devices" OR "steps counter device" OR "steps counter monitors" OR "steps counter monitor" OR "steps counter monitoring" OR "steps counting trackers" OR "steps counting tracker" OR "steps counting tracking" OR "steps counting devices" OR "steps counting device" OR "steps counting monitors" OR "steps counting monitor" OR "steps counting monitoring" OR "fitness wristband" OR "activity wristband" OR "step wristband" OR "steps wristband" OR "step-based wristband" OR "step count wristband" OR "step counts wristband" OR "step counter wristband" OR "step counting wristband" OR "steps count wristband" OR "steps counts wristband" OR "steps counter wristband" OR "steps counting wristband" OR "fitness wristbands" OR "activity wristbands" OR "step wristbands" OR "steps wristbands" OR "step-based wristbands" OR "step count wristbands" OR "step counts wristbands" OR "step counter wristbands" OR "step counting wristbands" OR "steps count wristbands" OR "steps counts wristbands" OR "steps counter wristbands" OR "steps counting wristbands" OR "fitness armband" OR "activity armband" OR "step armband" OR "steps armband" OR "step-based armband" OR "step count armband" OR "step counts armband" OR "step counter armband" OR "step counting armband" OR "steps count armband" OR "steps counts armband" OR "steps counter armband" OR "steps counting armband" OR "fitness armbands" OR "activity armbands" OR "step armbands" OR "steps armbands" OR "step-based armbands" OR "step count armbands" OR "step counts armbands" OR "step counter armbands" OR "step counting armbands" OR "steps count armbands" OR "steps counts armbands" OR "steps counter armbands" OR "steps counting armbands" OR "fitness bracelet" OR "activity bracelet" OR "step bracelet" OR "steps bracelet" OR "step-based bracelet" OR "step count bracelet" OR "step counts bracelet" OR "step counter bracelet" OR "step counting bracelet" OR "steps count bracelet" OR "steps counts bracelet" OR "steps counter bracelet" OR "steps counting bracelet" OR "fitness bracelets" OR "activity bracelets" OR "step bracelets" OR "steps bracelets" OR "step-based bracelets" OR "step count bracelets" OR "step counts bracelets" OR "step counter bracelets" OR "step counting bracelets" OR "steps count bracelets" OR "steps counts bracelets" OR "steps counter bracelets" OR "steps counting bracelets" OR "fitness band" OR "activity band" OR "step band" OR "steps band" OR "step-based band" OR "step count band" OR "step counts band" OR "step counter band" OR "step counting band" OR "steps count band" OR "steps counts band" OR "steps counter band" OR "steps counting band" OR "fitness bands" OR "activity bands" OR "step bands" OR "steps bands" OR "step-based bands" OR "step count bands" OR "step counts bands" OR "step counter bands" OR "step counting bands" OR "steps count bands" OR "steps counts bands" OR "steps counter bands" OR "steps counting bands" OR "wrist-based trackers" OR "wrist-based tracker" OR "wrist-based tracking" OR "wrist-based wearables" OR "wrist-based wearable" OR "wrist-based devices" OR "wrist-based device" OR "wrist-based monitors" OR "wrist-based monitor" OR "wrist-based monitoring" OR "wrist-based fitness" OR "wrist-based activity" OR "wrist-worn trackers" OR "wrist-worn tracker" OR "wrist-worn tracking" OR "wrist-worn wearables" OR "wrist-worn wearable" OR "wrist-worn devices" OR "wrist-worn device" OR "wrist-worn monitors" OR "wrist-worn monitor" OR "wrist-worn monitoring" OR "wrist-worn fitness" OR "wrist-worn activity" OR "arm-based trackers" OR "arm-based tracker" OR "arm-based tracking" OR "arm-based wearables" OR "arm-based wearable" OR "arm-based devices" OR "arm-based device" OR "arm-based monitors" OR "arm-based monitor" OR "arm-based monitoring" OR "arm-based fitness" OR "arm-based activity" OR "arm-worn trackers" OR "arm-worn tracker" OR "arm-worn tracking" OR "arm-worn wearables" OR "arm-worn wearable" OR "arm-worn devices" OR "arm-worn device" OR "arm-worn monitors" OR "arm-worn monitor" OR "arm-worn monitoring" OR "arm-worn fitness" OR "arm-worn activity" OR "hip-based trackers" OR "hip-based tracker" OR "hip-based tracking" OR "hip-based wearables" OR "hip-based wearable" OR "hip-based devices" OR "hip-based device" OR "hip-based monitors" OR "hip-based monitor" OR "hip-based monitoring" OR "hip-based fitness" OR "hip-based activity" OR "hip-worn trackers" OR "hip-worn tracker" OR "hip-worn tracking" OR "hip-worn wearables" OR "hip-worn wearable" OR "hip-worn devices" OR "hip-worn device" OR "hip-worn monitors" OR "hip-worn monitor" OR "hip-worn monitoring" OR "hip-worn fitness" OR "hip-worn activity" OR smartwatches OR smartwatch OR Fitbit OR Jawbone OR Samsung OR Xiaomi OR Garmin OR "Polar Loop" OR "Polar A300" OR "Polar A360" OR "Polar A370" OR Sony OR Huawei OR Misfit OR "LG Lifeband" OR "LG watch" OR "LG Gwatch" OR "LG G watch" OR Nike OR "Apple watch" OR Suunto) AND TITLE-ABS-KEY(program OR programs OR programme OR programmes OR training OR trainings OR intervention OR interventions OR treatment OR treatments OR "teaching unit" OR "teaching units" OR school OR schools OR "school-based" OR "physical education" OR PE OR "P.E." OR "physical education-based") AND TITLE-ABS-KEY("physical activity" OR "physical activities" OR exercise OR sedentary OR sedentarism OR steps OR step OR energy OR kcals OR kcal OR calories OR calorie OR kilocalories OR kilocalorie OR MET OR METS OR "metabolic equivalent" OR MVPA OR "vertical axis" OR "vector magnitude" OR counts)

1. **PUBMED**

(("self-tracker-based"[Title/Abstract] OR "self-tracker-supported"[Title/Abstract] OR "fitness self-trackers"[Title/Abstract] OR "activity self-trackers"[Title/Abstract] OR "wearable self-trackers"[Title/Abstract] OR "fitness self-tracker"[Title/Abstract] OR "activity self-tracker"[Title/Abstract] OR "wearable self-tracker"[Title/Abstract] OR "fitness self-tracking"[Title/Abstract] OR "activity self-tracking"[Title/Abstract] OR "wearable self-tracking"[Title/Abstract] OR "step self-trackers"[Title/Abstract] OR "step self-tracker"[Title/Abstract] OR "step self-tracking"[Title/Abstract] OR "steps self-trackers"[Title/Abstract] OR "steps self-tracker"[Title/Abstract] OR "steps self-tracking"[Title/Abstract] OR "step-based self-trackers"[Title/Abstract] OR "step-based self-tracker"[Title/Abstract] OR "step-based self-tracking"[Title/Abstract] OR "step count self-trackers"[Title/Abstract] OR "step count self-tracker"[Title/Abstract] OR "step count self-tracking"[Title/Abstract] OR "step counts self-trackers"[Title/Abstract] OR "step counts self-tracker"[Title/Abstract] OR "step counts self-tracking"[Title/Abstract] OR "step counter self-trackers"[Title/Abstract] OR "step counter self-tracker"[Title/Abstract] OR "step counter self-tracking"[Title/Abstract] OR "step counting self-trackers"[Title/Abstract] OR "step counting self-tracker"[Title/Abstract] OR "step counting self-tracking"[Title/Abstract] OR "steps count self-trackers"[Title/Abstract] OR "steps count self-tracker"[Title/Abstract] OR "steps count self-tracking"[Title/Abstract] OR "steps counts self-trackers"[Title/Abstract] OR "steps counts self-tracker"[Title/Abstract] OR "steps counts self-tracking"[Title/Abstract] OR "steps counter self-trackers"[Title/Abstract] OR "steps counter self-tracker"[Title/Abstract] OR "steps counter self-tracking"[Title/Abstract] OR "steps counting self-trackers"[Title/Abstract] OR "steps counting self-tracker"[Title/Abstract] OR "steps counting self-tracking"[Title/Abstract] OR "wrist-based self-trackers"[Title/Abstract] OR "wrist-based self-tracker"[Title/Abstract] OR "wrist-based self-tracking"[Title/Abstract] OR "wrist-worn self-trackers"[Title/Abstract] OR "wrist-worn self-tracker"[Title/Abstract] OR "wrist-worn self-tracking"[Title/Abstract] OR "arm-based self-trackers"[Title/Abstract] OR "arm-based self-tracker"[Title/Abstract] OR "arm-based self-tracking"[Title/Abstract] OR "arm-worn self-trackers"[Title/Abstract] OR "arm-worn self-tracker"[Title/Abstract] OR "arm-worn self-tracking"[Title/Abstract] OR "hip-based self-trackers"[Title/Abstract] OR "hip-based self-tracker"[Title/Abstract] OR "hip-based self-tracking"[Title/Abstract] OR "hip-worn self-trackers"[Title/Abstract] OR "hip-worn self-tracker"[Title/Abstract] OR "hip-worn self-tracking"[Title/Abstract] OR "wearable system"[Title/Abstract] OR "wearable systems"[Title/Abstract] OR "pedometer-based"[Title/Abstract] OR "tracker-based"[Title/Abstract] OR "wearable-based"[Title/Abstract] OR "technology-based"[Title/Abstract] OR "pedometer-supported"[Title/Abstract] OR "tracker-supported"[Title/Abstract] OR "wearable-supported"[Title/Abstract] OR "technology-supported"[Title/Abstract] OR "fitness sensors"[Title/Abstract] OR "activity sensors"[Title/Abstract] OR "wearable sensors"[Title/Abstract] OR "fitness sensor"[Title/Abstract] OR "activity sensor"[Title/Abstract] OR "wearable sensor"[Title/Abstract] OR "step sensors"[Title/Abstract] OR "step sensor"[Title/Abstract] OR "steps sensors"[Title/Abstract] OR "steps sensor"[Title/Abstract] OR "step-based sensors"[Title/Abstract] OR "step-based sensor"[Title/Abstract] OR "step count sensors"[Title/Abstract] OR "step count sensor"[Title/Abstract] OR "step counts sensors"[Title/Abstract] OR "step counts sensor"[Title/Abstract] OR "step counter sensors"[Title/Abstract] OR "step counter sensor"[Title/Abstract] OR "step counting sensors"[Title/Abstract] OR "step counting sensor"[Title/Abstract] OR "steps count sensors"[Title/Abstract] OR "steps count sensor"[Title/Abstract] OR "steps counts sensors"[Title/Abstract] OR "steps counts sensor"[Title/Abstract] OR "steps counter sensors"[Title/Abstract] OR "steps counter sensor"[Title/Abstract] OR "steps counting sensors"[Title/Abstract] OR "steps counting sensor"[Title/Abstract] OR "wrist-based sensors"[Title/Abstract] OR "wrist-based sensor"[Title/Abstract] OR "wrist-worn sensors"[Title/Abstract] OR "wrist-worn sensor"[Title/Abstract] OR "arm-based sensors"[Title/Abstract] OR "arm-based sensor"[Title/Abstract] OR "arm-worn sensors"[Title/Abstract] OR "arm-worn sensor"[Title/Abstract] OR "hip-based sensors"[Title/Abstract] OR "hip-based sensor"[Title/Abstract] OR "hip-worn sensors"[Title/Abstract] OR "hip-worn sensor"[Title/Abstract] OR "wearable technology"[Title/Abstract] OR "fitness trackers"[Title/Abstract] OR "activity trackers"[Title/Abstract] OR "wearable trackers"[Title/Abstract] OR "fitness tracker"[Title/Abstract] OR "activity tracker"[Title/Abstract] OR "wearable tracker"[Title/Abstract] OR "fitness tracking"[Title/Abstract] OR "activity tracking"[Title/Abstract] OR "wearable tracking"[Title/Abstract] OR "fitness wearables"[Title/Abstract] OR "activity wearables"[Title/Abstract] OR "fitness wearable"[Title/Abstract] OR "activity wearable"[Title/Abstract] OR "fitness devices"[Title/Abstract] OR "activity devices"[Title/Abstract] OR "wearable devices"[Title/Abstract] OR "fitness device"[Title/Abstract] OR "activity device"[Title/Abstract] OR "wearable device"[Title/Abstract] OR "fitness monitors"[Title/Abstract] OR "activity monitors"[Title/Abstract] OR "wearable monitors"[Title/Abstract] OR "fitness monitor"[Title/Abstract] OR "activity monitor"[Title/Abstract] OR "wearable monitor"[Title/Abstract] OR "fitness monitoring"[Title/Abstract] OR "activity monitoring"[Title/Abstract] OR "wearable monitoring"[Title/Abstract] OR pedometers[Title/Abstract] OR pedometer[Title/Abstract] OR "step trackers"[Title/Abstract] OR "step tracker"[Title/Abstract] OR "step tracking"[Title/Abstract] OR "step devices"[Title/Abstract] OR "step device"[Title/Abstract] OR "step monitors"[Title/Abstract] OR "step monitor"[Title/Abstract] OR "step monitoring"[Title/Abstract] OR "steps trackers"[Title/Abstract] OR "steps tracker"[Title/Abstract] OR "steps tracking"[Title/Abstract] OR "steps devices"[Title/Abstract] OR "steps device"[Title/Abstract] OR "steps monitors"[Title/Abstract] OR "steps monitor"[Title/Abstract] OR "steps monitoring"[Title/Abstract] OR "step-based trackers"[Title/Abstract] OR "step-based tracker"[Title/Abstract] OR "step-based tracking"[Title/Abstract] OR "step-based devices"[Title/Abstract] OR "step-based device"[Title/Abstract] OR "step-based monitors"[Title/Abstract] OR "step-based monitor"[Title/Abstract] OR "step-based monitoring"[Title/Abstract] OR "step count trackers"[Title/Abstract] OR "step count tracker"[Title/Abstract] OR "step count tracking"[Title/Abstract] OR "step count devices"[Title/Abstract] OR "step count device"[Title/Abstract] OR "step count monitors"[Title/Abstract] OR "step count monitor"[Title/Abstract] OR "step count monitoring"[Title/Abstract] OR "step counts trackers"[Title/Abstract] OR "step counts tracker"[Title/Abstract] OR "step counts tracking"[Title/Abstract] OR "step counts devices"[Title/Abstract] OR "step counts device"[Title/Abstract] OR "step counts monitors"[Title/Abstract] OR "step counts monitor"[Title/Abstract] OR "step counts monitoring"[Title/Abstract] OR "step counter trackers"[Title/Abstract] OR "step counter tracker"[Title/Abstract] OR "step counter tracking"[Title/Abstract] OR "step counter devices"[Title/Abstract] OR "step counter device"[Title/Abstract] OR "step counter monitors"[Title/Abstract] OR "step counter monitor"[Title/Abstract] OR "step counter monitoring"[Title/Abstract] OR "step counting trackers"[Title/Abstract] OR "step counting tracker"[Title/Abstract] OR "step counting tracking"[Title/Abstract] OR "step counting devices"[Title/Abstract] OR "step counting device"[Title/Abstract] OR "step counting monitors"[Title/Abstract] OR "step counting monitor"[Title/Abstract] OR "step counting monitoring"[Title/Abstract] OR "steps count trackers"[Title/Abstract] OR "steps count tracker"[Title/Abstract] OR "steps count tracking"[Title/Abstract] OR "steps count devices"[Title/Abstract] OR "steps count device"[Title/Abstract] OR "steps count monitors"[Title/Abstract] OR "steps count monitor"[Title/Abstract] OR "steps count monitoring"[Title/Abstract] OR "steps counts trackers"[Title/Abstract] OR "steps counts tracker"[Title/Abstract] OR "steps counts tracking"[Title/Abstract] OR "steps counts devices"[Title/Abstract] OR "steps counts device"[Title/Abstract] OR "steps counts monitors"[Title/Abstract] OR "steps counts monitor"[Title/Abstract] OR "steps counts monitoring"[Title/Abstract] OR "steps counter trackers"[Title/Abstract] OR "steps counter tracker"[Title/Abstract] OR "steps counter tracking"[Title/Abstract] OR "steps counter devices"[Title/Abstract] OR "steps counter device"[Title/Abstract] OR "steps counter monitors"[Title/Abstract] OR "steps counter monitor"[Title/Abstract] OR "steps counter monitoring"[Title/Abstract] OR "steps counting trackers"[Title/Abstract] OR "steps counting tracker"[Title/Abstract] OR "steps counting tracking"[Title/Abstract] OR "steps counting devices"[Title/Abstract] OR "steps counting device"[Title/Abstract] OR "steps counting monitors"[Title/Abstract] OR "steps counting monitor"[Title/Abstract] OR "steps counting monitoring"[Title/Abstract] OR "fitness wristband"[Title/Abstract] OR "activity wristband"[Title/Abstract] OR "step wristband"[Title/Abstract] OR "steps wristband"[Title/Abstract] OR "step-based wristband"[Title/Abstract] OR "step count wristband"[Title/Abstract] OR "step counts wristband"[Title/Abstract] OR "step counter wristband"[Title/Abstract] OR "step counting wristband"[Title/Abstract] OR "steps count wristband"[Title/Abstract] OR "steps counts wristband"[Title/Abstract] OR "steps counter wristband"[Title/Abstract] OR "steps counting wristband"[Title/Abstract] OR "fitness wristbands"[Title/Abstract] OR "activity wristbands"[Title/Abstract] OR "step wristbands"[Title/Abstract] OR "steps wristbands"[Title/Abstract] OR "step-based wristbands"[Title/Abstract] OR "step count wristbands"[Title/Abstract] OR "step counts wristbands"[Title/Abstract] OR "step counter wristbands"[Title/Abstract] OR "step counting wristbands"[Title/Abstract] OR "steps count wristbands"[Title/Abstract] OR "steps counts wristbands"[Title/Abstract] OR "steps counter wristbands"[Title/Abstract] OR "steps counting wristbands"[Title/Abstract] OR "fitness armband"[Title/Abstract] OR "activity armband"[Title/Abstract] OR "step armband"[Title/Abstract] OR "steps armband"[Title/Abstract] OR "step-based armband"[Title/Abstract] OR "step count armband"[Title/Abstract] OR "step counts armband"[Title/Abstract] OR "step counter armband"[Title/Abstract] OR "step counting armband"[Title/Abstract] OR "steps count armband"[Title/Abstract] OR "steps counts armband"[Title/Abstract] OR "steps counter armband"[Title/Abstract] OR "steps counting armband"[Title/Abstract] OR "fitness armbands"[Title/Abstract] OR "activity armbands"[Title/Abstract] OR "step armbands"[Title/Abstract] OR "steps armbands"[Title/Abstract] OR "step-based armbands"[Title/Abstract] OR "step count armbands"[Title/Abstract] OR "step counts armbands"[Title/Abstract] OR "step counter armbands"[Title/Abstract] OR "step counting armbands"[Title/Abstract] OR "steps count armbands"[Title/Abstract] OR "steps counts armbands"[Title/Abstract] OR "steps counter armbands"[Title/Abstract] OR "steps counting armbands"[Title/Abstract] OR "fitness bracelet"[Title/Abstract] OR "activity bracelet"[Title/Abstract] OR "step bracelet"[Title/Abstract] OR "steps bracelet"[Title/Abstract] OR "step-based bracelet"[Title/Abstract] OR "step count bracelet"[Title/Abstract] OR "step counts bracelet"[Title/Abstract] OR "step counter bracelet"[Title/Abstract] OR "step counting bracelet"[Title/Abstract] OR "steps count bracelet"[Title/Abstract] OR "steps counts bracelet"[Title/Abstract] OR "steps counter bracelet"[Title/Abstract] OR "steps counting bracelet"[Title/Abstract] OR "fitness bracelets"[Title/Abstract] OR "activity bracelets"[Title/Abstract] OR "step bracelets"[Title/Abstract] OR "steps bracelets"[Title/Abstract] OR "step-based bracelets"[Title/Abstract] OR "step count bracelets"[Title/Abstract] OR "step counts bracelets"[Title/Abstract] OR "step counter bracelets"[Title/Abstract] OR "step counting bracelets"[Title/Abstract] OR "steps count bracelets"[Title/Abstract] OR "steps counts bracelets"[Title/Abstract] OR "steps counter bracelets"[Title/Abstract] OR "steps counting bracelets"[Title/Abstract] OR "fitness band"[Title/Abstract] OR "activity band"[Title/Abstract] OR "step band"[Title/Abstract] OR "steps band"[Title/Abstract] OR "step-based band"[Title/Abstract] OR "step count band"[Title/Abstract] OR "step counts band"[Title/Abstract] OR "step counter band"[Title/Abstract] OR "step counting band"[Title/Abstract] OR "steps count band"[Title/Abstract] OR "steps counts band"[Title/Abstract] OR "steps counter band"[Title/Abstract] OR "steps counting band"[Title/Abstract] OR "fitness bands"[Title/Abstract] OR "activity bands"[Title/Abstract] OR "step bands"[Title/Abstract] OR "steps bands"[Title/Abstract] OR "step-based bands"[Title/Abstract] OR "step count bands"[Title/Abstract] OR "step counts bands"[Title/Abstract] OR "step counter bands"[Title/Abstract] OR "step counting bands"[Title/Abstract] OR "steps count bands"[Title/Abstract] OR "steps counts bands"[Title/Abstract] OR "steps counter bands"[Title/Abstract] OR "steps counting bands"[Title/Abstract] OR "wrist-based trackers"[Title/Abstract] OR "wrist-based tracker"[Title/Abstract] OR "wrist-based tracking"[Title/Abstract] OR "wrist-based wearables"[Title/Abstract] OR "wrist-based wearable"[Title/Abstract] OR "wrist-based devices"[Title/Abstract] OR "wrist-based device"[Title/Abstract] OR "wrist-based monitors"[Title/Abstract] OR "wrist-based monitor"[Title/Abstract] OR "wrist-based monitoring"[Title/Abstract] OR "wrist-based fitness"[Title/Abstract] OR "wrist-based activity"[Title/Abstract] OR "wrist-worn trackers"[Title/Abstract] OR "wrist-worn tracker"[Title/Abstract] OR "wrist-worn tracking"[Title/Abstract] OR "wrist-worn wearables"[Title/Abstract] OR "wrist-worn wearable"[Title/Abstract] OR "wrist-worn devices"[Title/Abstract] OR "wrist-worn device"[Title/Abstract] OR "wrist-worn monitors"[Title/Abstract] OR "wrist-worn monitor"[Title/Abstract] OR "wrist-worn monitoring"[Title/Abstract] OR "wrist-worn fitness"[Title/Abstract] OR "wrist-worn activity"[Title/Abstract] OR "arm-based trackers"[Title/Abstract] OR "arm-based tracker"[Title/Abstract] OR "arm-based tracking"[Title/Abstract] OR "arm-based wearables"[Title/Abstract] OR "arm-based wearable"[Title/Abstract] OR "arm-based devices"[Title/Abstract] OR "arm-based device"[Title/Abstract] OR "arm-based monitors"[Title/Abstract] OR "arm-based monitor"[Title/Abstract] OR "arm-based monitoring"[Title/Abstract] OR "arm-based fitness"[Title/Abstract] OR "arm-based activity"[Title/Abstract] OR "arm-worn trackers"[Title/Abstract] OR "arm-worn tracker"[Title/Abstract] OR "arm-worn tracking"[Title/Abstract] OR "arm-worn wearables"[Title/Abstract] OR "arm-worn wearable"[Title/Abstract] OR "arm-worn devices"[Title/Abstract] OR "arm-worn device"[Title/Abstract] OR "arm-worn monitors"[Title/Abstract] OR "arm-worn monitor"[Title/Abstract] OR "arm-worn monitoring"[Title/Abstract] OR "arm-worn fitness"[Title/Abstract] OR "arm-worn activity"[Title/Abstract] OR "hip-based trackers"[Title/Abstract] OR "hip-based tracker"[Title/Abstract] OR "hip-based tracking"[Title/Abstract] OR "hip-based wearables"[Title/Abstract] OR "hip-based wearable"[Title/Abstract] OR "hip-based devices"[Title/Abstract] OR "hip-based device"[Title/Abstract] OR "hip-based monitors"[Title/Abstract] OR "hip-based monitor"[Title/Abstract] OR "hip-based monitoring"[Title/Abstract] OR "hip-based fitness"[Title/Abstract] OR "hip-based activity"[Title/Abstract] OR "hip-worn trackers"[Title/Abstract] OR "hip-worn tracker"[Title/Abstract] OR "hip-worn tracking"[Title/Abstract] OR "hip-worn wearables"[Title/Abstract] OR "hip-worn wearable"[Title/Abstract] OR "hip-worn devices"[Title/Abstract] OR "hip-worn device"[Title/Abstract] OR "hip-worn monitors"[Title/Abstract] OR "hip-worn monitor"[Title/Abstract] OR "hip-worn monitoring"[Title/Abstract] OR "hip-worn fitness"[Title/Abstract] OR "hip-worn activity"[Title/Abstract] OR smartwatches[Title/Abstract] OR smartwatch[Title/Abstract] OR Fitbit[Title/Abstract] OR Jawbone[Title/Abstract] OR Samsung[Title/Abstract] OR Xiaomi[Title/Abstract] OR Garmin[Title/Abstract] OR "Polar Loop"[Title/Abstract] OR "Polar A300"[Title/Abstract] OR "Polar A360"[Title/Abstract] OR "Polar A370"[Title/Abstract] OR Sony[Title/Abstract] OR Huawei[Title/Abstract] OR Misfit[Title/Abstract] OR "LG Lifeband"[Title/Abstract] OR "LG watch"[Title/Abstract] OR "LG Gwatch"[Title/Abstract] OR "LG G watch"[Title/Abstract] OR Nike[Title/Abstract] OR "Apple watch"[Title/Abstract] OR Suunto[Title/Abstract]) AND (program[Title/Abstract] OR programs[Title/Abstract] OR programme[Title/Abstract] OR programmes[Title/Abstract] OR training[Title/Abstract] OR trainings[Title/Abstract] OR intervention[Title/Abstract] OR interventions[Title/Abstract] OR treatment[Title/Abstract] OR treatments[Title/Abstract] OR "teaching unit"[Title/Abstract] OR "teaching units"[Title/Abstract] OR school[Title/Abstract] OR schools[Title/Abstract] OR "school-based"[Title/Abstract] OR "physical education"[Title/Abstract] OR PE[Title/Abstract] OR "P.E."[Title/Abstract] OR "physical education-based"[Title/Abstract]) AND ("physical activity"[Title/Abstract] OR "physical activities"[Title/Abstract] OR exercise[Title/Abstract] OR sedentary[Title/Abstract] OR sedentarism[Title/Abstract] OR steps[Title/Abstract] OR step[Title/Abstract] OR energy[Title/Abstract] OR kcals[Title/Abstract] OR kcal[Title/Abstract] OR calories[Title/Abstract] OR calorie[Title/Abstract] OR kilocalories[Title/Abstract] OR kilocalorie[Title/Abstract] OR MET[Title/Abstract] OR METS[Title/Abstract] OR "metabolic equivalent"[Title/Abstract] OR MVPA[Title/Abstract] OR "vertical axis"[Title/Abstract] OR "vector magnitude"[Title/Abstract] OR counts[Title/Abstract]))

1. **SPORTDISCUS WITH FULL TEXT**

("self-tracker-based" OR "self-tracker-supported" OR "fitness self-trackers" OR "activity self-trackers" OR "wearable self-trackers" OR "fitness self-tracker" OR "activity self-tracker" OR "wearable self-tracker" OR "fitness self-tracking" OR "activity self-tracking" OR "wearable self-tracking" OR "step self-trackers" OR "step self-tracker" OR "step self-tracking" OR "steps self-trackers" OR "steps self-tracker" OR "steps self-tracking" OR "step-based self-trackers" OR "step-based self-tracker" OR "step-based self-tracking" OR "step count self-trackers" OR "step count self-tracker" OR "step count self-tracking" OR "step counts self-trackers" OR "step counts self-tracker" OR "step counts self-tracking" OR "step counter self-trackers" OR "step counter self-tracker" OR "step counter self-tracking" OR "step counting self-trackers" OR "step counting self-tracker" OR "step counting self-tracking" OR "steps count self-trackers" OR "steps count self-tracker" OR "steps count self-tracking" OR "steps counts self-trackers" OR "steps counts self-tracker" OR "steps counts self-tracking" OR "steps counter self-trackers" OR "steps counter self-tracker" OR "steps counter self-tracking" OR "steps counting self-trackers" OR "steps counting self-tracker" OR "steps counting self-tracking" OR "wrist-based self-trackers" OR "wrist-based self-tracker" OR "wrist-based self-tracking" OR "wrist-worn self-trackers" OR "wrist-worn self-tracker" OR "wrist-worn self-tracking" OR "arm-based self-trackers" OR "arm-based self-tracker" OR "arm-based self-tracking" OR "arm-worn self-trackers" OR "arm-worn self-tracker" OR "arm-worn self-tracking" OR "hip-based self-trackers" OR "hip-based self-tracker" OR "hip-based self-tracking" OR "hip-worn self-trackers" OR "hip-worn self-tracker" OR "hip-worn self-tracking" OR "wearable system" OR "wearable systems" OR "pedometer-based" OR "tracker-based" OR "wearable-based" OR "technology-based" OR "pedometer-supported" OR "tracker-supported" OR "wearable-supported" OR "technology-supported" OR "fitness sensors" OR "activity sensors" OR "wearable sensors" OR "fitness sensor" OR "activity sensor" OR "wearable sensor" OR "step sensors" OR "step sensor" OR "steps sensors" OR "steps sensor" OR "step-based sensors" OR "step-based sensor" OR "step count sensors" OR "step count sensor" OR "step counts sensors" OR "step counts sensor" OR "step counter sensors" OR "step counter sensor" OR "step counting sensors" OR "step counting sensor" OR "steps count sensors" OR "steps count sensor" OR "steps counts sensors" OR "steps counts sensor" OR "steps counter sensors" OR "steps counter sensor" OR "steps counting sensors" OR "steps counting sensor" OR "wrist-based sensors" OR "wrist-based sensor" OR "wrist-worn sensors" OR "wrist-worn sensor" OR "arm-based sensors" OR "arm-based sensor" OR "arm-worn sensors" OR "arm-worn sensor" OR "hip-based sensors" OR "hip-based sensor" OR "hip-worn sensors" OR "hip-worn sensor" OR "wearable technology" OR "fitness trackers" OR "activity trackers" OR "wearable trackers" OR "fitness tracker" OR "activity tracker" OR "wearable tracker" OR "fitness tracking" OR "activity tracking" OR "wearable tracking" OR "fitness wearables" OR "activity wearables" OR "fitness wearable" OR "activity wearable" OR "fitness devices" OR "activity devices" OR "wearable devices" OR "fitness device" OR "activity device" OR "wearable device" OR "fitness monitors" OR "activity monitors" OR "wearable monitors" OR "fitness monitor" OR "activity monitor" OR "wearable monitor" OR "fitness monitoring" OR "activity monitoring" OR "wearable monitoring" OR pedometers OR pedometer OR "step trackers" OR "step tracker" OR "step tracking" OR "step devices" OR "step device" OR "step monitors" OR "step monitor" OR "step monitoring" OR "steps trackers" OR "steps tracker" OR "steps tracking" OR "steps devices" OR "steps device" OR "steps monitors" OR "steps monitor" OR "steps monitoring" OR "step-based trackers" OR "step-based tracker" OR "step-based tracking" OR "step-based devices" OR "step-based device" OR "step-based monitors" OR "step-based monitor" OR "step-based monitoring" OR "step count trackers" OR "step count tracker" OR "step count tracking" OR "step count devices" OR "step count device" OR "step count monitors" OR "step count monitor" OR "step count monitoring" OR "step counts trackers" OR "step counts tracker" OR "step counts tracking" OR "step counts devices" OR "step counts device" OR "step counts monitors" OR "step counts monitor" OR "step counts monitoring" OR "step counter trackers" OR "step counter tracker" OR "step counter tracking" OR "step counter devices" OR "step counter device" OR "step counter monitors" OR "step counter monitor" OR "step counter monitoring" OR "step counting trackers" OR "step counting tracker" OR "step counting tracking" OR "step counting devices" OR "step counting device" OR "step counting monitors" OR "step counting monitor" OR "step counting monitoring" OR "steps count trackers" OR "steps count tracker" OR "steps count tracking" OR "steps count devices" OR "steps count device" OR "steps count monitors" OR "steps count monitor" OR "steps count monitoring" OR "steps counts trackers" OR "steps counts tracker" OR "steps counts tracking" OR "steps counts devices" OR "steps counts device" OR "steps counts monitors" OR "steps counts monitor" OR "steps counts monitoring" OR "steps counter trackers" OR "steps counter tracker" OR "steps counter tracking" OR "steps counter devices" OR "steps counter device" OR "steps counter monitors" OR "steps counter monitor" OR "steps counter monitoring" OR "steps counting trackers" OR "steps counting tracker" OR "steps counting tracking" OR "steps counting devices" OR "steps counting device" OR "steps counting monitors" OR "steps counting monitor" OR "steps counting monitoring" OR "fitness wristband" OR "activity wristband" OR "step wristband" OR "steps wristband" OR "step-based wristband" OR "step count wristband" OR "step counts wristband" OR "step counter wristband" OR "step counting wristband" OR "steps count wristband" OR "steps counts wristband" OR "steps counter wristband" OR "steps counting wristband" OR "fitness wristbands" OR "activity wristbands" OR "step wristbands" OR "steps wristbands" OR "step-based wristbands" OR "step count wristbands" OR "step counts wristbands" OR "step counter wristbands" OR "step counting wristbands" OR "steps count wristbands" OR "steps counts wristbands" OR "steps counter wristbands" OR "steps counting wristbands" OR "fitness armband" OR "activity armband" OR "step armband" OR "steps armband" OR "step-based armband" OR "step count armband" OR "step counts armband" OR "step counter armband" OR "step counting armband" OR "steps count armband" OR "steps counts armband" OR "steps counter armband" OR "steps counting armband" OR "fitness armbands" OR "activity armbands" OR "step armbands" OR "steps armbands" OR "step-based armbands" OR "step count armbands" OR "step counts armbands" OR "step counter armbands" OR "step counting armbands" OR "steps count armbands" OR "steps counts armbands" OR "steps counter armbands" OR "steps counting armbands" OR "fitness bracelet" OR "activity bracelet" OR "step bracelet" OR "steps bracelet" OR "step-based bracelet" OR "step count bracelet" OR "step counts bracelet" OR "step counter bracelet" OR "step counting bracelet" OR "steps count bracelet" OR "steps counts bracelet" OR "steps counter bracelet" OR "steps counting bracelet" OR "fitness bracelets" OR "activity bracelets" OR "step bracelets" OR "steps bracelets" OR "step-based bracelets" OR "step count bracelets" OR "step counts bracelets" OR "step counter bracelets" OR "step counting bracelets" OR "steps count bracelets" OR "steps counts bracelets" OR "steps counter bracelets" OR "steps counting bracelets" OR "fitness band" OR "activity band" OR "step band" OR "steps band" OR "step-based band" OR "step count band" OR "step counts band" OR "step counter band" OR "step counting band" OR "steps count band" OR "steps counts band" OR "steps counter band" OR "steps counting band" OR "fitness bands" OR "activity bands" OR "step bands" OR "steps bands" OR "step-based bands" OR "step count bands" OR "step counts bands" OR "step counter bands" OR "step counting bands" OR "steps count bands" OR "steps counts bands" OR "steps counter bands" OR "steps counting bands" OR "wrist-based trackers" OR "wrist-based tracker" OR "wrist-based tracking" OR "wrist-based wearables" OR "wrist-based wearable" OR "wrist-based devices" OR "wrist-based device" OR "wrist-based monitors" OR "wrist-based monitor" OR "wrist-based monitoring" OR "wrist-based fitness" OR "wrist-based activity" OR "wrist-worn trackers" OR "wrist-worn tracker" OR "wrist-worn tracking" OR "wrist-worn wearables" OR "wrist-worn wearable" OR "wrist-worn devices" OR "wrist-worn device" OR "wrist-worn monitors" OR "wrist-worn monitor" OR "wrist-worn monitoring" OR "wrist-worn fitness" OR "wrist-worn activity" OR "arm-based trackers" OR "arm-based tracker" OR "arm-based tracking" OR "arm-based wearables" OR "arm-based wearable" OR "arm-based devices" OR "arm-based device" OR "arm-based monitors" OR "arm-based monitor" OR "arm-based monitoring" OR "arm-based fitness" OR "arm-based activity" OR "arm-worn trackers" OR "arm-worn tracker" OR "arm-worn tracking" OR "arm-worn wearables" OR "arm-worn wearable" OR "arm-worn devices" OR "arm-worn device" OR "arm-worn monitors" OR "arm-worn monitor" OR "arm-worn monitoring" OR "arm-worn fitness" OR "arm-worn activity" OR "hip-based trackers" OR "hip-based tracker" OR "hip-based tracking" OR "hip-based wearables" OR "hip-based wearable" OR "hip-based devices" OR "hip-based device" OR "hip-based monitors" OR "hip-based monitor" OR "hip-based monitoring" OR "hip-based fitness" OR "hip-based activity" OR "hip-worn trackers" OR "hip-worn tracker" OR "hip-worn tracking" OR "hip-worn wearables" OR "hip-worn wearable" OR "hip-worn devices" OR "hip-worn device" OR "hip-worn monitors" OR "hip-worn monitor" OR "hip-worn monitoring" OR "hip-worn fitness" OR "hip-worn activity" OR smartwatches OR smartwatch OR Fitbit OR Jawbone OR Samsung OR Xiaomi OR Garmin OR "Polar Loop" OR "Polar A300" OR "Polar A360" OR "Polar A370" OR Sony OR Huawei OR Misfit OR "LG Lifeband" OR "LG watch" OR "LG Gwatch" OR "LG G watch" OR Nike OR "Apple watch" OR Suunto) AND ("P.E" OR "physical education-based" OR program OR programs OR programme OR programmes OR training OR trainings OR intervention OR interventions OR treatment OR treatments OR "teaching unit" OR "teaching units" OR school OR schools OR "school-based" OR "physical education" OR PE) AND ("physical activity" OR "physical activities" OR exercise OR sedentary OR sedentarism OR steps OR step OR energy OR kcals OR kcal OR calories OR calorie OR kilocalories OR kilocalorie OR MET OR METS OR "metabolic equivalent" OR MVPA OR "vertical axis" OR "vector magnitude" OR counts)

1. **CINAHL**

("self-tracker-based" OR "self-tracker-supported" OR "fitness self-trackers" OR "activity self-trackers" OR "wearable self-trackers" OR "fitness self-tracker" OR "activity self-tracker" OR "wearable self-tracker" OR "fitness self-tracking" OR "activity self-tracking" OR "wearable self-tracking" OR "step self-trackers" OR "step self-tracker" OR "step self-tracking" OR "steps self-trackers" OR "steps self-tracker" OR "steps self-tracking" OR "step-based self-trackers" OR "step-based self-tracker" OR "step-based self-tracking" OR "step count self-trackers" OR "step count self-tracker" OR "step count self-tracking" OR "step counts self-trackers" OR "step counts self-tracker" OR "step counts self-tracking" OR "step counter self-trackers" OR "step counter self-tracker" OR "step counter self-tracking" OR "step counting self-trackers" OR "step counting self-tracker" OR "step counting self-tracking" OR "steps count self-trackers" OR "steps count self-tracker" OR "steps count self-tracking" OR "steps counts self-trackers" OR "steps counts self-tracker" OR "steps counts self-tracking" OR "steps counter self-trackers" OR "steps counter self-tracker" OR "steps counter self-tracking" OR "steps counting self-trackers" OR "steps counting self-tracker" OR "steps counting self-tracking" OR "wrist-based self-trackers" OR "wrist-based self-tracker" OR "wrist-based self-tracking" OR "wrist-worn self-trackers" OR "wrist-worn self-tracker" OR "wrist-worn self-tracking" OR "arm-based self-trackers" OR "arm-based self-tracker" OR "arm-based self-tracking" OR "arm-worn self-trackers" OR "arm-worn self-tracker" OR "arm-worn self-tracking" OR "hip-based self-trackers" OR "hip-based self-tracker" OR "hip-based self-tracking" OR "hip-worn self-trackers" OR "hip-worn self-tracker" OR "hip-worn self-tracking" OR "wearable system" OR "wearable systems" OR "pedometer-based" OR "tracker-based" OR "wearable-based" OR "technology-based" OR "pedometer-supported" OR "tracker-supported" OR "wearable-supported" OR "technology-supported" OR "fitness sensors" OR "activity sensors" OR "wearable sensors" OR "fitness sensor" OR "activity sensor" OR "wearable sensor" OR "step sensors" OR "step sensor" OR "steps sensors" OR "steps sensor" OR "step-based sensors" OR "step-based sensor" OR "step count sensors" OR "step count sensor" OR "step counts sensors" OR "step counts sensor" OR "step counter sensors" OR "step counter sensor" OR "step counting sensors" OR "step counting sensor" OR "steps count sensors" OR "steps count sensor" OR "steps counts sensors" OR "steps counts sensor" OR "steps counter sensors" OR "steps counter sensor" OR "steps counting sensors" OR "steps counting sensor" OR "wrist-based sensors" OR "wrist-based sensor" OR "wrist-worn sensors" OR "wrist-worn sensor" OR "arm-based sensors" OR "arm-based sensor" OR "arm-worn sensors" OR "arm-worn sensor" OR "hip-based sensors" OR "hip-based sensor" OR "hip-worn sensors" OR "hip-worn sensor" OR "wearable technology" OR "fitness trackers" OR "activity trackers" OR "wearable trackers" OR "fitness tracker" OR "activity tracker" OR "wearable tracker" OR "fitness tracking" OR "activity tracking" OR "wearable tracking" OR "fitness wearables" OR "activity wearables" OR "fitness wearable" OR "activity wearable" OR "fitness devices" OR "activity devices" OR "wearable devices" OR "fitness device" OR "activity device" OR "wearable device" OR "fitness monitors" OR "activity monitors" OR "wearable monitors" OR "fitness monitor" OR "activity monitor" OR "wearable monitor" OR "fitness monitoring" OR "activity monitoring" OR "wearable monitoring" OR pedometers OR pedometer OR "step trackers" OR "step tracker" OR "step tracking" OR "step devices" OR "step device" OR "step monitors" OR "step monitor" OR "step monitoring" OR "steps trackers" OR "steps tracker" OR "steps tracking" OR "steps devices" OR "steps device" OR "steps monitors" OR "steps monitor" OR "steps monitoring" OR "step-based trackers" OR "step-based tracker" OR "step-based tracking" OR "step-based devices" OR "step-based device" OR "step-based monitors" OR "step-based monitor" OR "step-based monitoring" OR "step count trackers" OR "step count tracker" OR "step count tracking" OR "step count devices" OR "step count device" OR "step count monitors" OR "step count monitor" OR "step count monitoring" OR "step counts trackers" OR "step counts tracker" OR "step counts tracking" OR "step counts devices" OR "step counts device" OR "step counts monitors" OR "step counts monitor" OR "step counts monitoring" OR "step counter trackers" OR "step counter tracker" OR "step counter tracking" OR "step counter devices" OR "step counter device" OR "step counter monitors" OR "step counter monitor" OR "step counter monitoring" OR "step counting trackers" OR "step counting tracker" OR "step counting tracking" OR "step counting devices" OR "step counting device" OR "step counting monitors" OR "step counting monitor" OR "step counting monitoring" OR "steps count trackers" OR "steps count tracker" OR "steps count tracking" OR "steps count devices" OR "steps count device" OR "steps count monitors" OR "steps count monitor" OR "steps count monitoring" OR "steps counts trackers" OR "steps counts tracker" OR "steps counts tracking" OR "steps counts devices" OR "steps counts device" OR "steps counts monitors" OR "steps counts monitor" OR "steps counts monitoring" OR "steps counter trackers" OR "steps counter tracker" OR "steps counter tracking" OR "steps counter devices" OR "steps counter device" OR "steps counter monitors" OR "steps counter monitor" OR "steps counter monitoring" OR "steps counting trackers" OR "steps counting tracker" OR "steps counting tracking" OR "steps counting devices" OR "steps counting device" OR "steps counting monitors" OR "steps counting monitor" OR "steps counting monitoring" OR "fitness wristband" OR "activity wristband" OR "step wristband" OR "steps wristband" OR "step-based wristband" OR "step count wristband" OR "step counts wristband" OR "step counter wristband" OR "step counting wristband" OR "steps count wristband" OR "steps counts wristband" OR "steps counter wristband" OR "steps counting wristband" OR "fitness wristbands" OR "activity wristbands" OR "step wristbands" OR "steps wristbands" OR "step-based wristbands" OR "step count wristbands" OR "step counts wristbands" OR "step counter wristbands" OR "step counting wristbands" OR "steps count wristbands" OR "steps counts wristbands" OR "steps counter wristbands" OR "steps counting wristbands" OR "fitness armband" OR "activity armband" OR "step armband" OR "steps armband" OR "step-based armband" OR "step count armband" OR "step counts armband" OR "step counter armband" OR "step counting armband" OR "steps count armband" OR "steps counts armband" OR "steps counter armband" OR "steps counting armband" OR "fitness armbands" OR "activity armbands" OR "step armbands" OR "steps armbands" OR "step-based armbands" OR "step count armbands" OR "step counts armbands" OR "step counter armbands" OR "step counting armbands" OR "steps count armbands" OR "steps counts armbands" OR "steps counter armbands" OR "steps counting armbands" OR "fitness bracelet" OR "activity bracelet" OR "step bracelet" OR "steps bracelet" OR "step-based bracelet" OR "step count bracelet" OR "step counts bracelet" OR "step counter bracelet" OR "step counting bracelet" OR "steps count bracelet" OR "steps counts bracelet" OR "steps counter bracelet" OR "steps counting bracelet" OR "fitness bracelets" OR "activity bracelets" OR "step bracelets" OR "steps bracelets" OR "step-based bracelets" OR "step count bracelets" OR "step counts bracelets" OR "step counter bracelets" OR "step counting bracelets" OR "steps count bracelets" OR "steps counts bracelets" OR "steps counter bracelets" OR "steps counting bracelets" OR "fitness band" OR "activity band" OR "step band" OR "steps band" OR "step-based band" OR "step count band" OR "step counts band" OR "step counter band" OR "step counting band" OR "steps count band" OR "steps counts band" OR "steps counter band" OR "steps counting band" OR "fitness bands" OR "activity bands" OR "step bands" OR "steps bands" OR "step-based bands" OR "step count bands" OR "step counts bands" OR "step counter bands" OR "step counting bands" OR "steps count bands" OR "steps counts bands" OR "steps counter bands" OR "steps counting bands" OR "wrist-based trackers" OR "wrist-based tracker" OR "wrist-based tracking" OR "wrist-based wearables" OR "wrist-based wearable" OR "wrist-based devices" OR "wrist-based device" OR "wrist-based monitors" OR "wrist-based monitor" OR "wrist-based monitoring" OR "wrist-based fitness" OR "wrist-based activity" OR "wrist-worn trackers" OR "wrist-worn tracker" OR "wrist-worn tracking" OR "wrist-worn wearables" OR "wrist-worn wearable" OR "wrist-worn devices" OR "wrist-worn device" OR "wrist-worn monitors" OR "wrist-worn monitor" OR "wrist-worn monitoring" OR "wrist-worn fitness" OR "wrist-worn activity" OR "arm-based trackers" OR "arm-based tracker" OR "arm-based tracking" OR "arm-based wearables" OR "arm-based wearable" OR "arm-based devices" OR "arm-based device" OR "arm-based monitors" OR "arm-based monitor" OR "arm-based monitoring" OR "arm-based fitness" OR "arm-based activity" OR "arm-worn trackers" OR "arm-worn tracker" OR "arm-worn tracking" OR "arm-worn wearables" OR "arm-worn wearable" OR "arm-worn devices" OR "arm-worn device" OR "arm-worn monitors" OR "arm-worn monitor" OR "arm-worn monitoring" OR "arm-worn fitness" OR "arm-worn activity" OR "hip-based trackers" OR "hip-based tracker" OR "hip-based tracking" OR "hip-based wearables" OR "hip-based wearable" OR "hip-based devices" OR "hip-based device" OR "hip-based monitors" OR "hip-based monitor" OR "hip-based monitoring" OR "hip-based fitness" OR "hip-based activity" OR "hip-worn trackers" OR "hip-worn tracker" OR "hip-worn tracking" OR "hip-worn wearables" OR "hip-worn wearable" OR "hip-worn devices" OR "hip-worn device" OR "hip-worn monitors" OR "hip-worn monitor" OR "hip-worn monitoring" OR "hip-worn fitness" OR "hip-worn activity" OR smartwatches OR smartwatch OR Fitbit OR Jawbone OR Samsung OR Xiaomi OR Garmin OR "Polar Loop" OR "Polar A300" OR "Polar A360" OR "Polar A370" OR Sony OR Huawei OR Misfit OR "LG Lifeband" OR "LG watch" OR "LG Gwatch" OR "LG G watch" OR Nike OR "Apple watch" OR Suunto) AND (program OR programs OR programme OR programmes OR training OR trainings OR intervention OR interventions OR treatment OR treatments OR "teaching unit" OR "teaching units" OR school OR schools OR "school-based" OR "physical education" OR PE OR "P.E." OR "physical education-based") AND ("physical activity" OR "physical activities" OR exercise OR sedentary OR sedentarism OR steps OR step OR energy OR kcals OR kcal OR calories OR calorie OR kilocalories OR kilocalorie OR MET OR METS OR "metabolic equivalent" OR MVPA OR "vertical axis" OR "vector magnitude" OR counts)

1. **COCHRANE LIBRARY**

("self-tracker-based" OR "self-tracker-supported" OR "fitness self-trackers" OR "activity self-trackers" OR "wearable self-trackers" OR "fitness self-tracker" OR "activity self-tracker" OR "wearable self-tracker" OR "fitness self-tracking" OR "activity self-tracking" OR "wearable self-tracking" OR "step self-trackers" OR "step self-tracker" OR "step self-tracking" OR "steps self-trackers" OR "steps self-tracker" OR "steps self-tracking" OR "step-based self-trackers" OR "step-based self-tracker" OR "step-based self-tracking" OR "step count self-trackers" OR "step count self-tracker" OR "step count self-tracking" OR "step counts self-trackers" OR "step counts self-tracker" OR "step counts self-tracking" OR "step counter self-trackers" OR "step counter self-tracker" OR "step counter self-tracking" OR "step counting self-trackers" OR "step counting self-tracker" OR "step counting self-tracking" OR "steps count self-trackers" OR "steps count self-tracker" OR "steps count self-tracking" OR "steps counts self-trackers" OR "steps counts self-tracker" OR "steps counts self-tracking" OR "steps counter self-trackers" OR "steps counter self-tracker" OR "steps counter self-tracking" OR "steps counting self-trackers" OR "steps counting self-tracker" OR "steps counting self-tracking" OR "wrist-based self-trackers" OR "wrist-based self-tracker" OR "wrist-based self-tracking" OR "wrist-worn self-trackers" OR "wrist-worn self-tracker" OR "wrist-worn self-tracking" OR "arm-based self-trackers" OR "arm-based self-tracker" OR "arm-based self-tracking" OR "arm-worn self-trackers" OR "arm-worn self-tracker" OR "arm-worn self-tracking" OR "hip-based self-trackers" OR "hip-based self-tracker" OR "hip-based self-tracking" OR "hip-worn self-trackers" OR "hip-worn self-tracker" OR "hip-worn self-tracking" OR "wearable system" OR "wearable systems" OR "pedometer-based" OR "tracker-based" OR "wearable-based" OR "technology-based" OR "pedometer-supported" OR "tracker-supported" OR "wearable-supported" OR "technology-supported" OR "fitness sensors" OR "activity sensors" OR "wearable sensors" OR "fitness sensor" OR "activity sensor" OR "wearable sensor" OR "step sensors" OR "step sensor" OR "steps sensors" OR "steps sensor" OR "step-based sensors" OR "step-based sensor" OR "step count sensors" OR "step count sensor" OR "step counts sensors" OR "step counts sensor" OR "step counter sensors" OR "step counter sensor" OR "step counting sensors" OR "step counting sensor" OR "steps count sensors" OR "steps count sensor" OR "steps counts sensors" OR "steps counts sensor" OR "steps counter sensors" OR "steps counter sensor" OR "steps counting sensors" OR "steps counting sensor" OR "wrist-based sensors" OR "wrist-based sensor" OR "wrist-worn sensors" OR "wrist-worn sensor" OR "arm-based sensors" OR "arm-based sensor" OR "arm-worn sensors" OR "arm-worn sensor" OR "hip-based sensors" OR "hip-based sensor" OR "hip-worn sensors" OR "hip-worn sensor" OR "wearable technology" OR "fitness trackers" OR "activity trackers" OR "wearable trackers" OR "fitness tracker" OR "activity tracker" OR "wearable tracker" OR "fitness tracking" OR "activity tracking" OR "wearable tracking" OR "fitness wearables" OR "activity wearables" OR "fitness wearable" OR "activity wearable" OR "fitness devices" OR "activity devices" OR "wearable devices" OR "fitness device" OR "activity device" OR "wearable device" OR "fitness monitors" OR "activity monitors" OR "wearable monitors" OR "fitness monitor" OR "activity monitor" OR "wearable monitor" OR "fitness monitoring" OR "activity monitoring" OR "wearable monitoring" OR pedometers OR pedometer OR "step trackers" OR "step tracker" OR "step tracking" OR "step devices" OR "step device" OR "step monitors" OR "step monitor" OR "step monitoring" OR "steps trackers" OR "steps tracker" OR "steps tracking" OR "steps devices" OR "steps device" OR "steps monitors" OR "steps monitor" OR "steps monitoring" OR "step-based trackers" OR "step-based tracker" OR "step-based tracking" OR "step-based devices" OR "step-based device" OR "step-based monitors" OR "step-based monitor" OR "step-based monitoring" OR "step count trackers" OR "step count tracker" OR "step count tracking" OR "step count devices" OR "step count device" OR "step count monitors" OR "step count monitor" OR "step count monitoring" OR "step counts trackers" OR "step counts tracker" OR "step counts tracking" OR "step counts devices" OR "step counts device" OR "step counts monitors" OR "step counts monitor" OR "step counts monitoring" OR "step counter trackers" OR "step counter tracker" OR "step counter tracking" OR "step counter devices" OR "step counter device" OR "step counter monitors" OR "step counter monitor" OR "step counter monitoring" OR "step counting trackers" OR "step counting tracker" OR "step counting tracking" OR "step counting devices" OR "step counting device" OR "step counting monitors" OR "step counting monitor" OR "step counting monitoring" OR "steps count trackers" OR "steps count tracker" OR "steps count tracking" OR "steps count devices" OR "steps count device" OR "steps count monitors" OR "steps count monitor" OR "steps count monitoring" OR "steps counts trackers" OR "steps counts tracker" OR "steps counts tracking" OR "steps counts devices" OR "steps counts device" OR "steps counts monitors" OR "steps counts monitor" OR "steps counts monitoring" OR "steps counter trackers" OR "steps counter tracker" OR "steps counter tracking" OR "steps counter devices" OR "steps counter device" OR "steps counter monitors" OR "steps counter monitor" OR "steps counter monitoring" OR "steps counting trackers" OR "steps counting tracker" OR "steps counting tracking" OR "steps counting devices" OR "steps counting device" OR "steps counting monitors" OR "steps counting monitor" OR "steps counting monitoring" OR "fitness wristband" OR "activity wristband" OR "step wristband" OR "steps wristband" OR "step-based wristband" OR "step count wristband" OR "step counts wristband" OR "step counter wristband" OR "step counting wristband" OR "steps count wristband" OR "steps counts wristband" OR "steps counter wristband" OR "steps counting wristband" OR "fitness wristbands" OR "activity wristbands" OR "step wristbands" OR "steps wristbands" OR "step-based wristbands" OR "step count wristbands" OR "step counts wristbands" OR "step counter wristbands" OR "step counting wristbands" OR "steps count wristbands" OR "steps counts wristbands" OR "steps counter wristbands" OR "steps counting wristbands" OR "fitness armband" OR "activity armband" OR "step armband" OR "steps armband" OR "step-based armband" OR "step count armband" OR "step counts armband" OR "step counter armband" OR "step counting armband" OR "steps count armband" OR "steps counts armband" OR "steps counter armband" OR "steps counting armband" OR "fitness armbands" OR "activity armbands" OR "step armbands" OR "steps armbands" OR "step-based armbands" OR "step count armbands" OR "step counts armbands" OR "step counter armbands" OR "step counting armbands" OR "steps count armbands" OR "steps counts armbands" OR "steps counter armbands" OR "steps counting armbands" OR "fitness bracelet" OR "activity bracelet" OR "step bracelet" OR "steps bracelet" OR "step-based bracelet" OR "step count bracelet" OR "step counts bracelet" OR "step counter bracelet" OR "step counting bracelet" OR "steps count bracelet" OR "steps counts bracelet" OR "steps counter bracelet" OR "steps counting bracelet" OR "fitness bracelets" OR "activity bracelets" OR "step bracelets" OR "steps bracelets" OR "step-based bracelets" OR "step count bracelets" OR "step counts bracelets" OR "step counter bracelets" OR "step counting bracelets" OR "steps count bracelets" OR "steps counts bracelets" OR "steps counter bracelets" OR "steps counting bracelets" OR "fitness band" OR "activity band" OR "step band" OR "steps band" OR "step-based band" OR "step count band" OR "step counts band" OR "step counter band" OR "step counting band" OR "steps count band" OR "steps counts band" OR "steps counter band" OR "steps counting band" OR "fitness bands" OR "activity bands" OR "step bands" OR "steps bands" OR "step-based bands" OR "step count bands" OR "step counts bands" OR "step counter bands" OR "step counting bands" OR "steps count bands" OR "steps counts bands" OR "steps counter bands" OR "steps counting bands" OR "wrist-based trackers" OR "wrist-based tracker" OR "wrist-based tracking" OR "wrist-based wearables" OR "wrist-based wearable" OR "wrist-based devices" OR "wrist-based device" OR "wrist-based monitors" OR "wrist-based monitor" OR "wrist-based monitoring" OR "wrist-based fitness" OR "wrist-based activity" OR "wrist-worn trackers" OR "wrist-worn tracker" OR "wrist-worn tracking" OR "wrist-worn wearables" OR "wrist-worn wearable" OR "wrist-worn devices" OR "wrist-worn device" OR "wrist-worn monitors" OR "wrist-worn monitor" OR "wrist-worn monitoring" OR "wrist-worn fitness" OR "wrist-worn activity" OR "arm-based trackers" OR "arm-based tracker" OR "arm-based tracking" OR "arm-based wearables" OR "arm-based wearable" OR "arm-based devices" OR "arm-based device" OR "arm-based monitors" OR "arm-based monitor" OR "arm-based monitoring" OR "arm-based fitness" OR "arm-based activity" OR "arm-worn trackers" OR "arm-worn tracker" OR "arm-worn tracking" OR "arm-worn wearables" OR "arm-worn wearable" OR "arm-worn devices" OR "arm-worn device" OR "arm-worn monitors" OR "arm-worn monitor" OR "arm-worn monitoring" OR "arm-worn fitness" OR "arm-worn activity" OR "hip-based trackers" OR "hip-based tracker" OR "hip-based tracking" OR "hip-based wearables" OR "hip-based wearable" OR "hip-based devices" OR "hip-based device" OR "hip-based monitors" OR "hip-based monitor" OR "hip-based monitoring" OR "hip-based fitness" OR "hip-based activity" OR "hip-worn trackers" OR "hip-worn tracker" OR "hip-worn tracking" OR "hip-worn wearables" OR "hip-worn wearable" OR "hip-worn devices" OR "hip-worn device" OR "hip-worn monitors" OR "hip-worn monitor" OR "hip-worn monitoring" OR "hip-worn fitness" OR "hip-worn activity" OR smartwatches OR smartwatch OR Fitbit OR Jawbone OR Samsung OR Xiaomi OR Garmin OR "Polar Loop" OR "Polar A300" OR "Polar A360" OR "Polar A370" OR Sony OR Huawei OR Misfit OR "LG Lifeband" OR "LG watch" OR "LG Gwatch" OR "LG G watch" OR Nike OR "Apple watch" OR Suunto) AND (program OR programs OR programme OR programmes OR training OR trainings OR intervention OR interventions OR treatment OR treatments OR "teaching unit" OR "teaching units" OR school OR schools OR "school-based" OR "physical education" OR PE OR "P.E." OR "physical education-based") AND ("physical activity" OR "physical activities" OR exercise OR sedentary OR sedentarism OR steps OR step OR energy OR kcals OR kcal OR calories OR calorie OR kilocalories OR kilocalorie OR MET OR METS OR "metabolic equivalent" OR MVPA OR "vertical axis" OR "vector magnitude" OR counts)

1. **PROQUEST SOCIAL SCIENCES PREMIUM COLLECTION**

ALL(("self-tracker-based" OR "self-tracker-supported" OR "fitness self-trackers" OR "activity self-trackers" OR "wearable self-trackers" OR "fitness self-tracker" OR "activity self-tracker" OR "wearable self-tracker" OR "fitness self-tracking" OR "activity self-tracking" OR "wearable self-tracking" OR "step self-trackers" OR "step self-tracker" OR "step self-tracking" OR "steps self-trackers" OR "steps self-tracker" OR "steps self-tracking" OR "step-based self-trackers" OR "step-based self-tracker" OR "step-based self-tracking" OR "step count self-trackers" OR "step count self-tracker" OR "step count self-tracking" OR "step counts self-trackers" OR "step counts self-tracker" OR "step counts self-tracking" OR "step counter self-trackers" OR "step counter self-tracker" OR "step counter self-tracking" OR "step counting self-trackers" OR "step counting self-tracker" OR "step counting self-tracking" OR "steps count self-trackers" OR "steps count self-tracker" OR "steps count self-tracking" OR "steps counts self-trackers" OR "steps counts self-tracker" OR "steps counts self-tracking" OR "steps counter self-trackers" OR "steps counter self-tracker" OR "steps counter self-tracking" OR "steps counting self-trackers" OR "steps counting self-tracker" OR "steps counting self-tracking" OR "wrist-based self-trackers" OR "wrist-based self-tracker" OR "wrist-based self-tracking" OR "wrist-worn self-trackers" OR "wrist-worn self-tracker" OR "wrist-worn self-tracking" OR "arm-based self-trackers" OR "arm-based self-tracker" OR "arm-based self-tracking" OR "arm-worn self-trackers" OR "arm-worn self-tracker" OR "arm-worn self-tracking" OR "hip-based self-trackers" OR "hip-based self-tracker" OR "hip-based self-tracking" OR "hip-worn self-trackers" OR "hip-worn self-tracker" OR "hip-worn self-tracking" OR "wearable system" OR "wearable systems" OR "pedometer-based" OR "tracker-based" OR "wearable-based" OR "technology-based" OR "pedometer-supported" OR "tracker-supported" OR "wearable-supported" OR "technology-supported" OR "fitness sensors" OR "activity sensors" OR "wearable sensors" OR "fitness sensor" OR "activity sensor" OR "wearable sensor" OR "step sensors" OR "step sensor" OR "steps sensors" OR "steps sensor" OR "step-based sensors" OR "step-based sensor" OR "step count sensors" OR "step count sensor" OR "step counts sensors" OR "step counts sensor" OR "step counter sensors" OR "step counter sensor" OR "step counting sensors" OR "step counting sensor" OR "steps count sensors" OR "steps count sensor" OR "steps counts sensors" OR "steps counts sensor" OR "steps counter sensors" OR "steps counter sensor" OR "steps counting sensors" OR "steps counting sensor" OR "wrist-based sensors" OR "wrist-based sensor" OR "wrist-worn sensors" OR "wrist-worn sensor" OR "arm-based sensors" OR "arm-based sensor" OR "arm-worn sensors" OR "arm-worn sensor" OR "hip-based sensors" OR "hip-based sensor" OR "hip-worn sensors" OR "hip-worn sensor" OR "wearable technology" OR "fitness trackers" OR "activity trackers" OR "wearable trackers" OR "fitness tracker" OR "activity tracker" OR "wearable tracker" OR "fitness tracking" OR "activity tracking" OR "wearable tracking" OR "fitness wearables" OR "activity wearables" OR "fitness wearable" OR "activity wearable" OR "fitness devices" OR "activity devices" OR "wearable devices" OR "fitness device" OR "activity device" OR "wearable device" OR "fitness monitors" OR "activity monitors" OR "wearable monitors" OR "fitness monitor" OR "activity monitor" OR "wearable monitor" OR "fitness monitoring" OR "activity monitoring" OR "wearable monitoring" OR pedometers OR pedometer OR "step trackers" OR "step tracker" OR "step tracking" OR "step devices" OR "step device" OR "step monitors" OR "step monitor" OR "step monitoring" OR "steps trackers" OR "steps tracker" OR "steps tracking" OR "steps devices" OR "steps device" OR "steps monitors" OR "steps monitor" OR "steps monitoring" OR "step-based trackers" OR "step-based tracker" OR "step-based tracking" OR "step-based devices" OR "step-based device" OR "step-based monitors" OR "step-based monitor" OR "step-based monitoring" OR "step count trackers" OR "step count tracker" OR "step count tracking" OR "step count devices" OR "step count device" OR "step count monitors" OR "step count monitor" OR "step count monitoring" OR "step counts trackers" OR "step counts tracker" OR "step counts tracking" OR "step counts devices" OR "step counts device" OR "step counts monitors" OR "step counts monitor" OR "step counts monitoring" OR "step counter trackers" OR "step counter tracker" OR "step counter tracking" OR "step counter devices" OR "step counter device" OR "step counter monitors" OR "step counter monitor" OR "step counter monitoring" OR "step counting trackers" OR "step counting tracker" OR "step counting tracking" OR "step counting devices" OR "step counting device" OR "step counting monitors" OR "step counting monitor" OR "step counting monitoring" OR "steps count trackers" OR "steps count tracker" OR "steps count tracking" OR "steps count devices" OR "steps count device" OR "steps count monitors" OR "steps count monitor" OR "steps count monitoring" OR "steps counts trackers" OR "steps counts tracker" OR "steps counts tracking" OR "steps counts devices" OR "steps counts device" OR "steps counts monitors" OR "steps counts monitor" OR "steps counts monitoring" OR "steps counter trackers" OR "steps counter tracker" OR "steps counter tracking" OR "steps counter devices" OR "steps counter device" OR "steps counter monitors" OR "steps counter monitor" OR "steps counter monitoring" OR "steps counting trackers" OR "steps counting tracker" OR "steps counting tracking" OR "steps counting devices" OR "steps counting device" OR "steps counting monitors" OR "steps counting monitor" OR "steps counting monitoring" OR "fitness wristband" OR "activity wristband" OR "step wristband" OR "steps wristband" OR "step-based wristband" OR "step count wristband" OR "step counts wristband" OR "step counter wristband" OR "step counting wristband" OR "steps count wristband" OR "steps counts wristband" OR "steps counter wristband" OR "steps counting wristband" OR "fitness wristbands" OR "activity wristbands" OR "step wristbands" OR "steps wristbands" OR "step-based wristbands" OR "step count wristbands" OR "step counts wristbands" OR "step counter wristbands" OR "step counting wristbands" OR "steps count wristbands" OR "steps counts wristbands" OR "steps counter wristbands" OR "steps counting wristbands" OR "fitness armband" OR "activity armband" OR "step armband" OR "steps armband" OR "step-based armband" OR "step count armband" OR "step counts armband" OR "step counter armband" OR "step counting armband" OR "steps count armband" OR "steps counts armband" OR "steps counter armband" OR "steps counting armband" OR "fitness armbands" OR "activity armbands" OR "step armbands" OR "steps armbands" OR "step-based armbands" OR "step count armbands" OR "step counts armbands" OR "step counter armbands" OR "step counting armbands" OR "steps count armbands" OR "steps counts armbands" OR "steps counter armbands" OR "steps counting armbands" OR "fitness bracelet" OR "activity bracelet" OR "step bracelet" OR "steps bracelet" OR "step-based bracelet" OR "step count bracelet" OR "step counts bracelet" OR "step counter bracelet" OR "step counting bracelet" OR "steps count bracelet" OR "steps counts bracelet" OR "steps counter bracelet" OR "steps counting bracelet" OR "fitness bracelets" OR "activity bracelets" OR "step bracelets" OR "steps bracelets" OR "step-based bracelets" OR "step count bracelets" OR "step counts bracelets" OR "step counter bracelets" OR "step counting bracelets" OR "steps count bracelets" OR "steps counts bracelets" OR "steps counter bracelets" OR "steps counting bracelets" OR "fitness band" OR "activity band" OR "step band" OR "steps band" OR "step-based band" OR "step count band" OR "step counts band" OR "step counter band" OR "step counting band" OR "steps count band" OR "steps counts band" OR "steps counter band" OR "steps counting band" OR "fitness bands" OR "activity bands" OR "step bands" OR "steps bands" OR "step-based bands" OR "step count bands" OR "step counts bands" OR "step counter bands" OR "step counting bands" OR "steps count bands" OR "steps counts bands" OR "steps counter bands" OR "steps counting bands" OR "wrist-based trackers" OR "wrist-based tracker" OR "wrist-based tracking" OR "wrist-based wearables" OR "wrist-based wearable" OR "wrist-based devices" OR "wrist-based device" OR "wrist-based monitors" OR "wrist-based monitor" OR "wrist-based monitoring" OR "wrist-based fitness" OR "wrist-based activity" OR "wrist-worn trackers" OR "wrist-worn tracker" OR "wrist-worn tracking" OR "wrist-worn wearables" OR "wrist-worn wearable" OR "wrist-worn devices" OR "wrist-worn device" OR "wrist-worn monitors" OR "wrist-worn monitor" OR "wrist-worn monitoring" OR "wrist-worn fitness" OR "wrist-worn activity" OR "arm-based trackers" OR "arm-based tracker" OR "arm-based tracking" OR "arm-based wearables" OR "arm-based wearable" OR "arm-based devices" OR "arm-based device" OR "arm-based monitors" OR "arm-based monitor" OR "arm-based monitoring" OR "arm-based fitness" OR "arm-based activity" OR "arm-worn trackers" OR "arm-worn tracker" OR "arm-worn tracking" OR "arm-worn wearables" OR "arm-worn wearable" OR "arm-worn devices" OR "arm-worn device" OR "arm-worn monitors" OR "arm-worn monitor" OR "arm-worn monitoring" OR "arm-worn fitness" OR "arm-worn activity" OR "hip-based trackers" OR "hip-based tracker" OR "hip-based tracking" OR "hip-based wearables" OR "hip-based wearable" OR "hip-based devices" OR "hip-based device" OR "hip-based monitors" OR "hip-based monitor" OR "hip-based monitoring" OR "hip-based fitness" OR "hip-based activity" OR "hip-worn trackers" OR "hip-worn tracker" OR "hip-worn tracking" OR "hip-worn wearables" OR "hip-worn wearable" OR "hip-worn devices" OR "hip-worn device" OR "hip-worn monitors" OR "hip-worn monitor" OR "hip-worn monitoring" OR "hip-worn fitness" OR "hip-worn activity" OR smartwatches OR smartwatch OR Fitbit OR Jawbone OR Samsung OR Xiaomi OR Garmin OR "Polar Loop" OR "Polar A300" OR "Polar A360" OR "Polar A370" OR Sony OR Huawei OR Misfit OR "LG Lifeband" OR "LG watch" OR "LG Gwatch" OR "LG G watch" OR Nike OR "Apple watch" OR Suunto) AND (program OR programs OR programme OR programmes OR training OR trainings OR intervention OR interventions OR treatment OR treatments OR "teaching unit" OR "teaching units" OR school OR schools OR "school-based" OR "physical education" OR PE OR "P.E." OR "physical education-based") AND ("physical activity" OR "physical activities" OR exercise OR sedentary OR sedentarism OR steps OR step OR energy OR kcals OR kcal OR calories OR calorie OR kilocalories OR kilocalorie OR MET OR METS OR "metabolic equivalent" OR MVPA OR "vertical axis" OR "vector magnitude" OR counts))

1. **PROQUEST DISSERTARTIONS AND THESES GLOBAL**

ALL(("self-tracker-based" OR "self-tracker-supported" OR "fitness self-trackers" OR "activity self-trackers" OR "wearable self-trackers" OR "fitness self-tracker" OR "activity self-tracker" OR "wearable self-tracker" OR "fitness self-tracking" OR "activity self-tracking" OR "wearable self-tracking" OR "step self-trackers" OR "step self-tracker" OR "step self-tracking" OR "steps self-trackers" OR "steps self-tracker" OR "steps self-tracking" OR "step-based self-trackers" OR "step-based self-tracker" OR "step-based self-tracking" OR "step count self-trackers" OR "step count self-tracker" OR "step count self-tracking" OR "step counts self-trackers" OR "step counts self-tracker" OR "step counts self-tracking" OR "step counter self-trackers" OR "step counter self-tracker" OR "step counter self-tracking" OR "step counting self-trackers" OR "step counting self-tracker" OR "step counting self-tracking" OR "steps count self-trackers" OR "steps count self-tracker" OR "steps count self-tracking" OR "steps counts self-trackers" OR "steps counts self-tracker" OR "steps counts self-tracking" OR "steps counter self-trackers" OR "steps counter self-tracker" OR "steps counter self-tracking" OR "steps counting self-trackers" OR "steps counting self-tracker" OR "steps counting self-tracking" OR "wrist-based self-trackers" OR "wrist-based self-tracker" OR "wrist-based self-tracking" OR "wrist-worn self-trackers" OR "wrist-worn self-tracker" OR "wrist-worn self-tracking" OR "arm-based self-trackers" OR "arm-based self-tracker" OR "arm-based self-tracking" OR "arm-worn self-trackers" OR "arm-worn self-tracker" OR "arm-worn self-tracking" OR "hip-based self-trackers" OR "hip-based self-tracker" OR "hip-based self-tracking" OR "hip-worn self-trackers" OR "hip-worn self-tracker" OR "hip-worn self-tracking" OR "wearable system" OR "wearable systems" OR "pedometer-based" OR "tracker-based" OR "wearable-based" OR "technology-based" OR "pedometer-supported" OR "tracker-supported" OR "wearable-supported" OR "technology-supported" OR "fitness sensors" OR "activity sensors" OR "wearable sensors" OR "fitness sensor" OR "activity sensor" OR "wearable sensor" OR "step sensors" OR "step sensor" OR "steps sensors" OR "steps sensor" OR "step-based sensors" OR "step-based sensor" OR "step count sensors" OR "step count sensor" OR "step counts sensors" OR "step counts sensor" OR "step counter sensors" OR "step counter sensor" OR "step counting sensors" OR "step counting sensor" OR "steps count sensors" OR "steps count sensor" OR "steps counts sensors" OR "steps counts sensor" OR "steps counter sensors" OR "steps counter sensor" OR "steps counting sensors" OR "steps counting sensor" OR "wrist-based sensors" OR "wrist-based sensor" OR "wrist-worn sensors" OR "wrist-worn sensor" OR "arm-based sensors" OR "arm-based sensor" OR "arm-worn sensors" OR "arm-worn sensor" OR "hip-based sensors" OR "hip-based sensor" OR "hip-worn sensors" OR "hip-worn sensor" OR "wearable technology" OR "fitness trackers" OR "activity trackers" OR "wearable trackers" OR "fitness tracker" OR "activity tracker" OR "wearable tracker" OR "fitness tracking" OR "activity tracking" OR "wearable tracking" OR "fitness wearables" OR "activity wearables" OR "fitness wearable" OR "activity wearable" OR "fitness devices" OR "activity devices" OR "wearable devices" OR "fitness device" OR "activity device" OR "wearable device" OR "fitness monitors" OR "activity monitors" OR "wearable monitors" OR "fitness monitor" OR "activity monitor" OR "wearable monitor" OR "fitness monitoring" OR "activity monitoring" OR "wearable monitoring" OR pedometers OR pedometer OR "step trackers" OR "step tracker" OR "step tracking" OR "step devices" OR "step device" OR "step monitors" OR "step monitor" OR "step monitoring" OR "steps trackers" OR "steps tracker" OR "steps tracking" OR "steps devices" OR "steps device" OR "steps monitors" OR "steps monitor" OR "steps monitoring" OR "step-based trackers" OR "step-based tracker" OR "step-based tracking" OR "step-based devices" OR "step-based device" OR "step-based monitors" OR "step-based monitor" OR "step-based monitoring" OR "step count trackers" OR "step count tracker" OR "step count tracking" OR "step count devices" OR "step count device" OR "step count monitors" OR "step count monitor" OR "step count monitoring" OR "step counts trackers" OR "step counts tracker" OR "step counts tracking" OR "step counts devices" OR "step counts device" OR "step counts monitors" OR "step counts monitor" OR "step counts monitoring" OR "step counter trackers" OR "step counter tracker" OR "step counter tracking" OR "step counter devices" OR "step counter device" OR "step counter monitors" OR "step counter monitor" OR "step counter monitoring" OR "step counting trackers" OR "step counting tracker" OR "step counting tracking" OR "step counting devices" OR "step counting device" OR "step counting monitors" OR "step counting monitor" OR "step counting monitoring" OR "steps count trackers" OR "steps count tracker" OR "steps count tracking" OR "steps count devices" OR "steps count device" OR "steps count monitors" OR "steps count monitor" OR "steps count monitoring" OR "steps counts trackers" OR "steps counts tracker" OR "steps counts tracking" OR "steps counts devices" OR "steps counts device" OR "steps counts monitors" OR "steps counts monitor" OR "steps counts monitoring" OR "steps counter trackers" OR "steps counter tracker" OR "steps counter tracking" OR "steps counter devices" OR "steps counter device" OR "steps counter monitors" OR "steps counter monitor" OR "steps counter monitoring" OR "steps counting trackers" OR "steps counting tracker" OR "steps counting tracking" OR "steps counting devices" OR "steps counting device" OR "steps counting monitors" OR "steps counting monitor" OR "steps counting monitoring" OR "fitness wristband" OR "activity wristband" OR "step wristband" OR "steps wristband" OR "step-based wristband" OR "step count wristband" OR "step counts wristband" OR "step counter wristband" OR "step counting wristband" OR "steps count wristband" OR "steps counts wristband" OR "steps counter wristband" OR "steps counting wristband" OR "fitness wristbands" OR "activity wristbands" OR "step wristbands" OR "steps wristbands" OR "step-based wristbands" OR "step count wristbands" OR "step counts wristbands" OR "step counter wristbands" OR "step counting wristbands" OR "steps count wristbands" OR "steps counts wristbands" OR "steps counter wristbands" OR "steps counting wristbands" OR "fitness armband" OR "activity armband" OR "step armband" OR "steps armband" OR "step-based armband" OR "step count armband" OR "step counts armband" OR "step counter armband" OR "step counting armband" OR "steps count armband" OR "steps counts armband" OR "steps counter armband" OR "steps counting armband" OR "fitness armbands" OR "activity armbands" OR "step armbands" OR "steps armbands" OR "step-based armbands" OR "step count armbands" OR "step counts armbands" OR "step counter armbands" OR "step counting armbands" OR "steps count armbands" OR "steps counts armbands" OR "steps counter armbands" OR "steps counting armbands" OR "fitness bracelet" OR "activity bracelet" OR "step bracelet" OR "steps bracelet" OR "step-based bracelet" OR "step count bracelet" OR "step counts bracelet" OR "step counter bracelet" OR "step counting bracelet" OR "steps count bracelet" OR "steps counts bracelet" OR "steps counter bracelet" OR "steps counting bracelet" OR "fitness bracelets" OR "activity bracelets" OR "step bracelets" OR "steps bracelets" OR "step-based bracelets" OR "step count bracelets" OR "step counts bracelets" OR "step counter bracelets" OR "step counting bracelets" OR "steps count bracelets" OR "steps counts bracelets" OR "steps counter bracelets" OR "steps counting bracelets" OR "fitness band" OR "activity band" OR "step band" OR "steps band" OR "step-based band" OR "step count band" OR "step counts band" OR "step counter band" OR "step counting band" OR "steps count band" OR "steps counts band" OR "steps counter band" OR "steps counting band" OR "fitness bands" OR "activity bands" OR "step bands" OR "steps bands" OR "step-based bands" OR "step count bands" OR "step counts bands" OR "step counter bands" OR "step counting bands" OR "steps count bands" OR "steps counts bands" OR "steps counter bands" OR "steps counting bands" OR "wrist-based trackers" OR "wrist-based tracker" OR "wrist-based tracking" OR "wrist-based wearables" OR "wrist-based wearable" OR "wrist-based devices" OR "wrist-based device" OR "wrist-based monitors" OR "wrist-based monitor" OR "wrist-based monitoring" OR "wrist-based fitness" OR "wrist-based activity" OR "wrist-worn trackers" OR "wrist-worn tracker" OR "wrist-worn tracking" OR "wrist-worn wearables" OR "wrist-worn wearable" OR "wrist-worn devices" OR "wrist-worn device" OR "wrist-worn monitors" OR "wrist-worn monitor" OR "wrist-worn monitoring" OR "wrist-worn fitness" OR "wrist-worn activity" OR "arm-based trackers" OR "arm-based tracker" OR "arm-based tracking" OR "arm-based wearables" OR "arm-based wearable" OR "arm-based devices" OR "arm-based device" OR "arm-based monitors" OR "arm-based monitor" OR "arm-based monitoring" OR "arm-based fitness" OR "arm-based activity" OR "arm-worn trackers" OR "arm-worn tracker" OR "arm-worn tracking" OR "arm-worn wearables" OR "arm-worn wearable" OR "arm-worn devices" OR "arm-worn device" OR "arm-worn monitors" OR "arm-worn monitor" OR "arm-worn monitoring" OR "arm-worn fitness" OR "arm-worn activity" OR "hip-based trackers" OR "hip-based tracker" OR "hip-based tracking" OR "hip-based wearables" OR "hip-based wearable" OR "hip-based devices" OR "hip-based device" OR "hip-based monitors" OR "hip-based monitor" OR "hip-based monitoring" OR "hip-based fitness" OR "hip-based activity" OR "hip-worn trackers" OR "hip-worn tracker" OR "hip-worn tracking" OR "hip-worn wearables" OR "hip-worn wearable" OR "hip-worn devices" OR "hip-worn device" OR "hip-worn monitors" OR "hip-worn monitor" OR "hip-worn monitoring" OR "hip-worn fitness" OR "hip-worn activity" OR smartwatches OR smartwatch OR Fitbit OR Jawbone OR Samsung OR Xiaomi OR Garmin OR "Polar Loop" OR "Polar A300" OR "Polar A360" OR "Polar A370" OR Sony OR Huawei OR Misfit OR "LG Lifeband" OR "LG watch" OR "LG Gwatch" OR "LG G watch" OR Nike OR "Apple watch" OR Suunto) AND (program OR programs OR programme OR programmes OR training OR trainings OR intervention OR interventions OR treatment OR treatments OR "teaching unit" OR "teaching units" OR school OR schools OR "school-based" OR "physical education" OR PE OR "P.E." OR "physical education-based") AND ("physical activity" OR "physical activities" OR exercise OR sedentary OR sedentarism OR steps OR step OR energy OR kcals OR kcal OR calories OR calorie OR kilocalories OR kilocalorie OR MET OR METS OR "metabolic equivalent" OR MVPA OR "vertical axis" OR "vector magnitude" OR counts))
