# Supplementary material for: Effects of Consumer-Wearable Activity Tracker-Based Programs on Objectively Measured Daily Physical Activity and Sedentary Behavior Among School-Aged Children: A Systematic Review and Meta-analysis
Source: Sports Med Open. 2022 Jan 31;8:18. doi: 10.1186/s40798-021-00407-6 (PMC8804065; doi:10.1186/s40798-021-00407-6)
Supplement: Supplementary file 3 — Additional file 3. Coding form followed in the present systematic review. [file 40798_2021_407_MOESM3_ESM.doc]

| Supplementary File 3. Coding form followed in the present systematic review^a^ | |
| --- | --- |
| Variable | Definition |
| *Study characteristics* | |
| Reference number | Running numbers to identify studies |
| Study reference | First author et al. (year) |
| Publication date | Year of publication |
| Date of data collection | Year or range of years were the data collection were performed |
| Study design | Presence or absence of a control group |
| Sequence generation | Random; Cluster; or Non-random |
| Suspicion of selective outcomes | In the study protocol or the publication methods section, authors mentioned or not the measurement of some variables which outcomes were not reported |
| Total initial sample size (*n*) | Number of participants who met the inclusion criteria and begin the study |
| Total final sample size (*n*) | Number of participants finally included in the results |
| *Individuals’ characteristics* |  |
| Age | Mean and range in years |
| Sex | Males; Females; or Males and females |
| *Outcome measures pertaining to PA and or SB* | |
| Measurement moment | Pre-post-intervention measures; or only post-intervention measures |
| Kind of measurement instrument | Waist-worn consumer-wearable activity tracker; Wrist-worn consumer-wearable activity tracker; or Research-grade tracker |
| Measurement score and units | Total steps; Moderate-to-vigorous PA (in percentage per day); Moderate-to-vigorous PA (in minutes per day); Total PA (in percentage); Total PA (in minutes per day); Sedentary time (in percentage); Sedentary time (in minutes per day) |
| *Program characteristics* |  |
| Intervention length | Duration of the program in weeks |
| Consumer-wearable activity tracker | Waist-worn activity tracker; Wrist-worn activity tracker |
| Kind of goal-setting | Static goal; Adaptive goal; or No goal-setting |
| Diary | Presence or absence of a diary, logbook o similar where daily or weekly the child writes down daily PA score |
| Counseling | Presence or absence of educational counseling by talks or fliers about topics including, but not limited to, PA benefits, PA recommendations, strategies to achieve the established goal or strategies to solve PA barriers |
| Reminders | Presence or absence of daily or weekly reminders to encourage children to move and/or achieve the established goal by including, but not limited to, phone messages, activity tracker, or researchers, Physical Education teachers or parents |
| Motivational strategies | Presence or absence of any other strategy to encourage children to move or achieve the established goal including, but not limited to, incentives, social networks or competitions |
| Exercise routine | Presence or absence of any autonomous or monitor-guided training program schedule for children at least once a week during the intervention program |
| *Results of the program for each group* | |
| Initial and final group size | Number of participants included for each group |
| Pre- and post-intervention standard deviation | Standard deviation value of the pre- and post-intervention measure for each group |
| Pre-post intervention mean difference score | Mean differences between pre and post-intervention measures for each group. In the case of reporting mean of the pre- and post-intervention measure, they were registered and the mean difference was also calculated. |
| *Observations* | Observations were also registered when special issues were found |
| ^a^ The definition included in this table is a summary synthesis, but that coding form was designed in greater detail including definitions, examples, counterexamples or probably location in the manuscript. PA = Physical activity; SB = Sedentary behavior. | |
