# Supplementary material for: Effects of Consumer-Wearable Activity Tracker-Based Programs on Objectively Measured Daily Physical Activity and Sedentary Behavior Among School-Aged Children: A Systematic Review and Meta-analysis
Source: Sports Med Open. 2022 Jan 31;8:18. doi: 10.1186/s40798-021-00407-6 (PMC8804065; doi:10.1186/s40798-021-00407-6)
Supplement: Supplementary file 4 — Additional file 4. Algorithms followed for assessing methodological risk-of-bias in each domain. [file 40798_2021_407_MOESM4_ESM.docx]

Supplementary File 4. Algorithms followed for assessing methodological risk-of-bias in each domain.

1. **Bias arising from the randomization process**


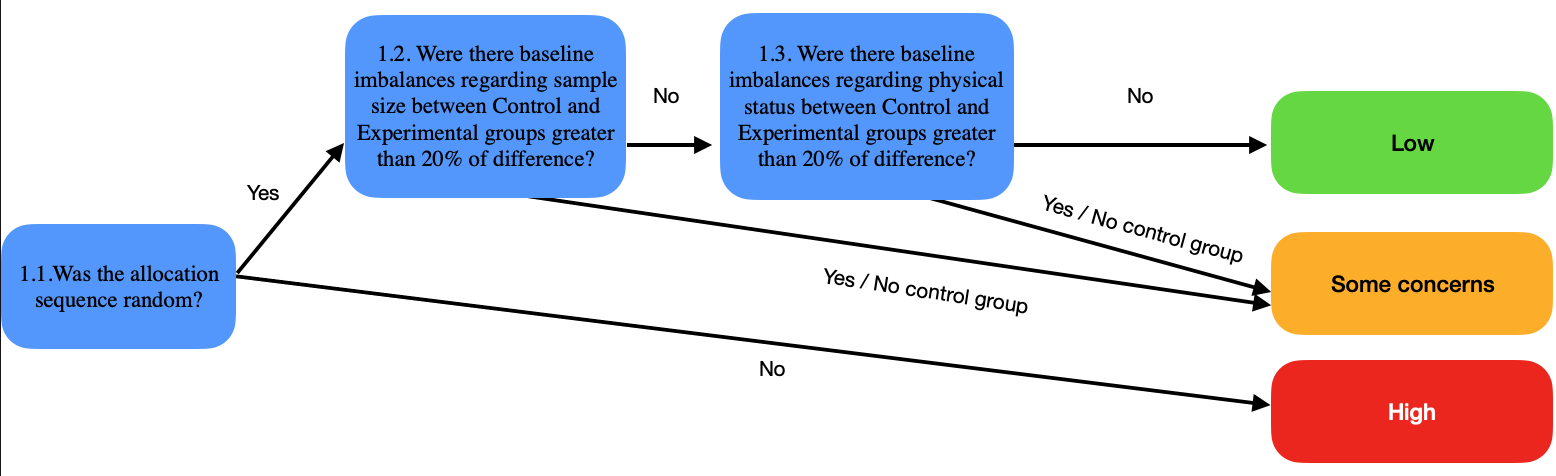


1. **Bias due to missing outcome data^a^**

**
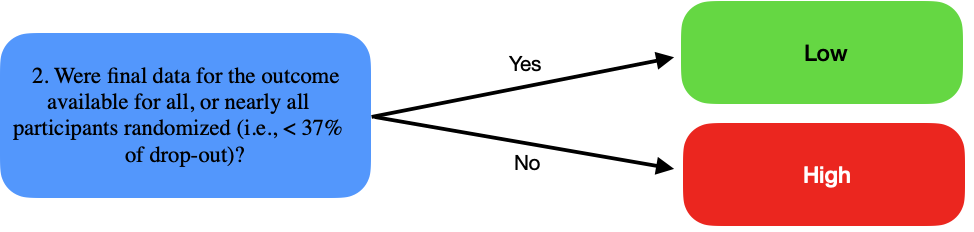
**

1. **Bias in measurement of the outcome**

**
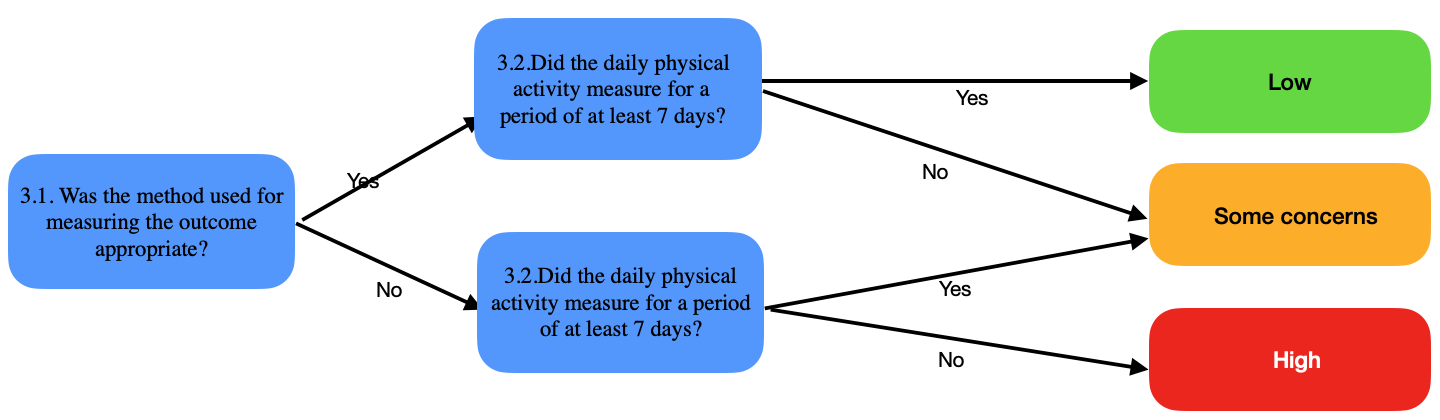
**

1. **Bias in selection of the reported results**

**
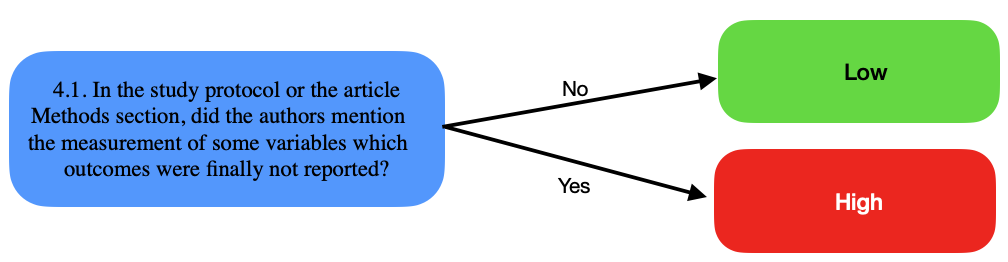
**

^a^ The 37% is the mean value for missingness during physical activity interventions among schoolchildren which used accelerometers to measure physical activity [1]

- 1. Howie E, Straker L. Rates of attrition, non-compliance and missingness in randomized controlled trials of child physical activity interventions using accelerometers: A brief methodological review. *J Sci Med Sport.* 2016;19(10):830–36.
