# Supplementary material for: Effects of Consumer-Wearable Activity Tracker-Based Programs on Objectively Measured Daily Physical Activity and Sedentary Behavior Among School-Aged Children: A Systematic Review and Meta-analysis
Source: Sports Med Open. 2022 Jan 31;8:18. doi: 10.1186/s40798-021-00407-6 (PMC8804065; doi:10.1186/s40798-021-00407-6)
Supplement: Supplementary file 5 — Additional file 5. Risk of bias assessment: (a) Daily steps units of analysis; (b) Moderate-to-vigorous physical activity units of analysis; (c) Total physical activity unit of analysis; and (d) Sedentary behavior unit of analysis. Each row corresponds to a unit of analysis. Green symbols represent a “Low risk of bias”, yellow symbols represent “Some concerns”, and red symbols represent a “High risk of bias”. [file 40798_2021_407_MOESM5_ESM.docx]

Supplementary Figure 5. Risk of bias assessment: (a) Daily steps units of analysis; (b) Moderate-to-vigorous physical activity units of analysis; (c) Total physical activity unit of analysis; and (d) Sedentary behavior unit of analysis. Each row corresponds to a unit of analysis. Green symbols represent a “Low risk of bias”, yellow symbols represent “Some concerns”, and red symbols represent a “High risk of bias”.


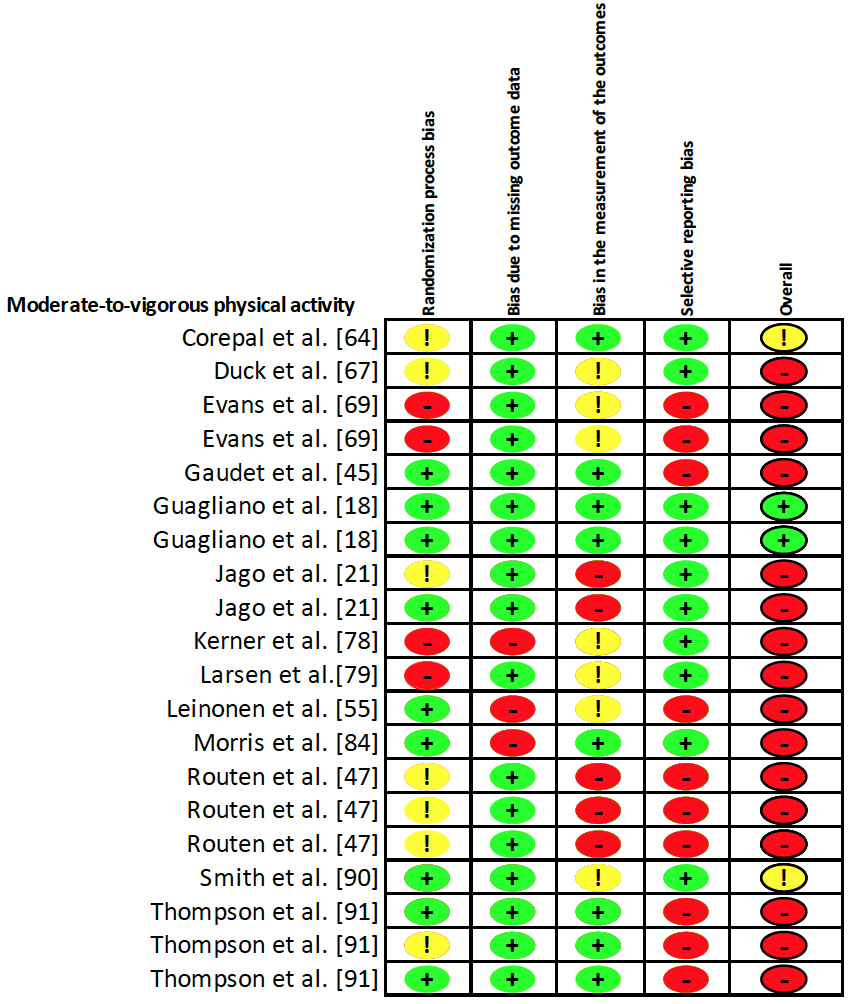

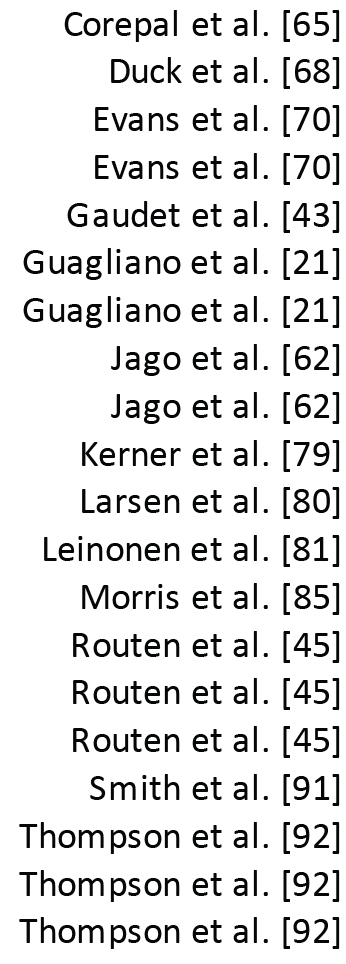

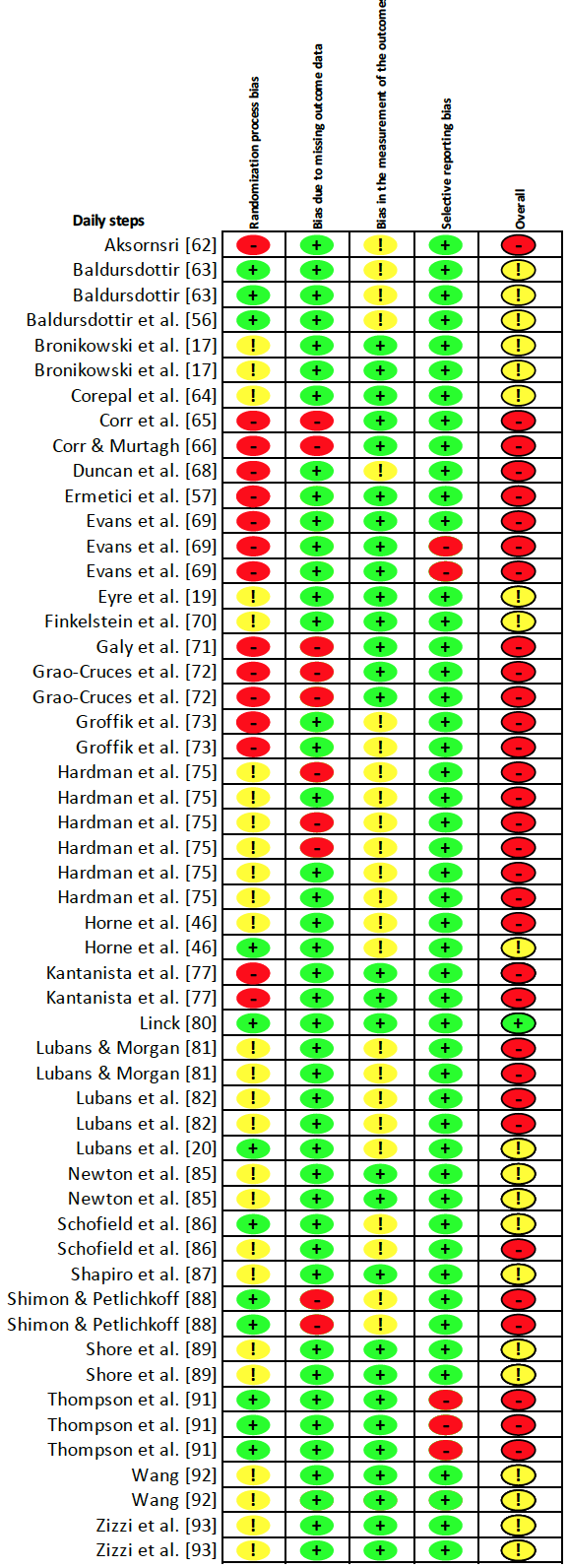

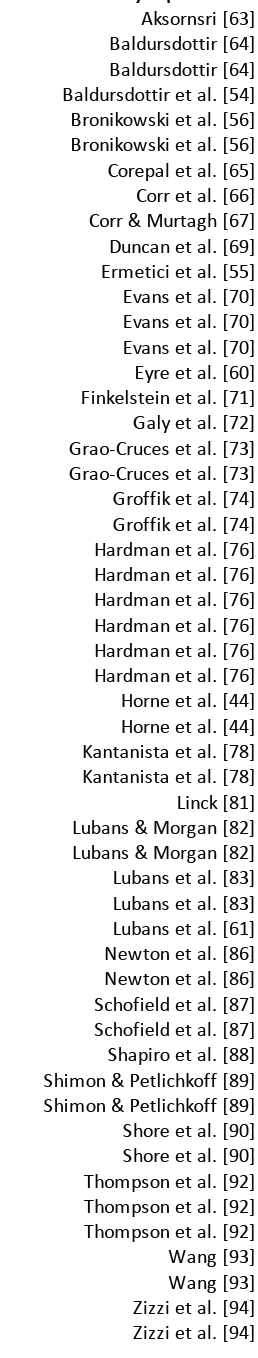


**(a) (b)**


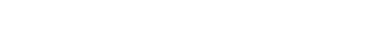


**Total physical activity**

**(c)**


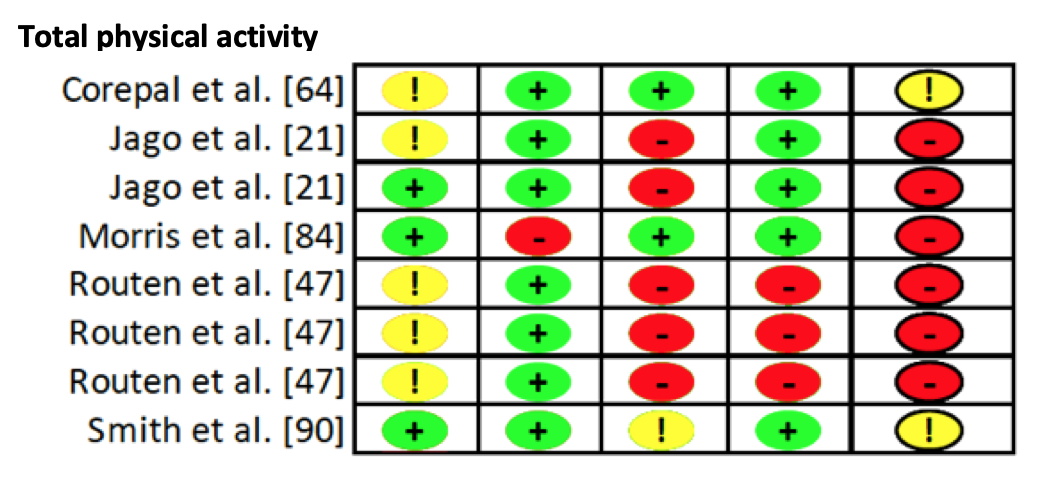

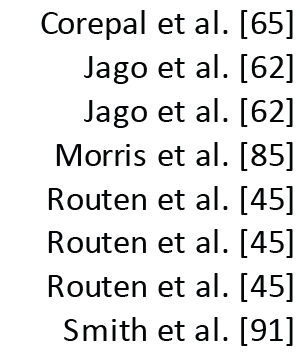


**
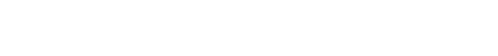
(d)**


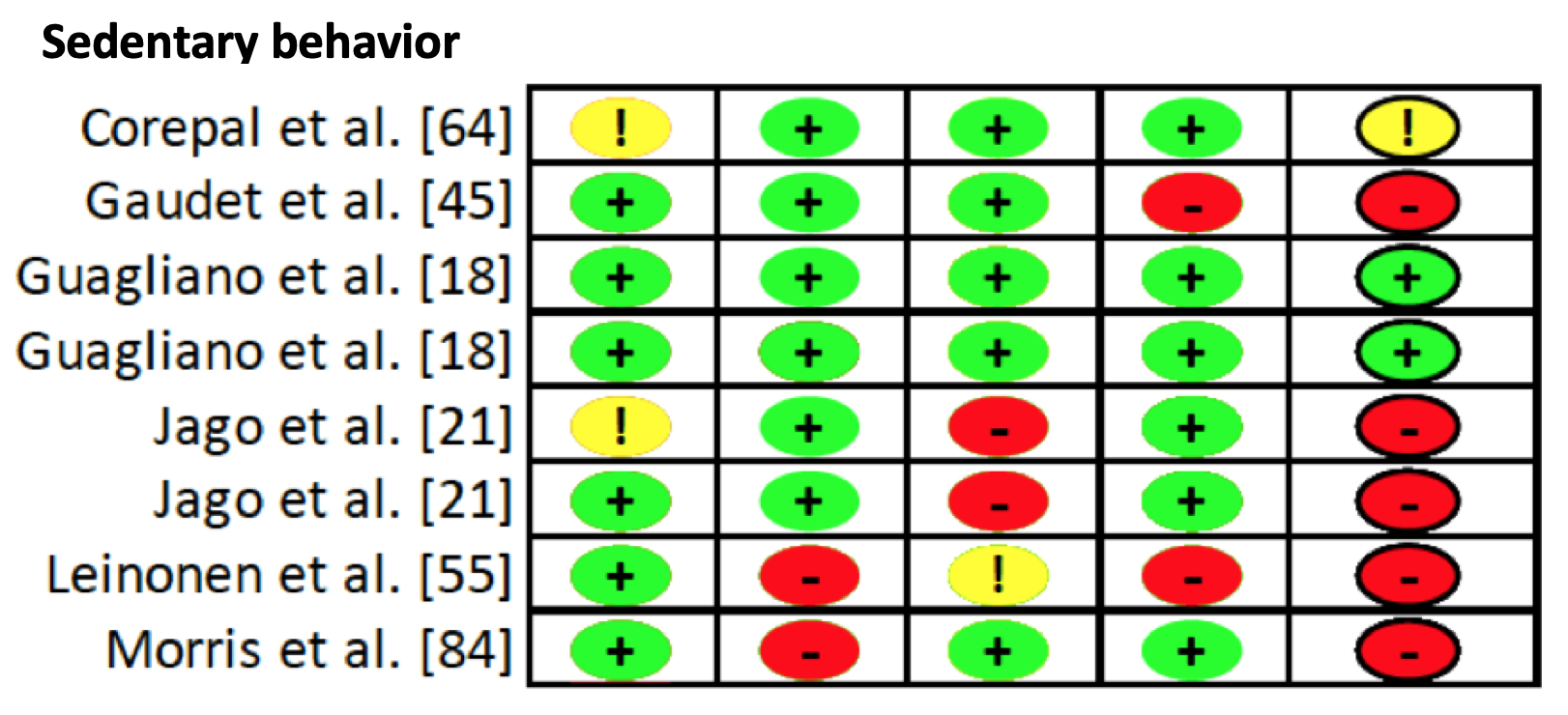

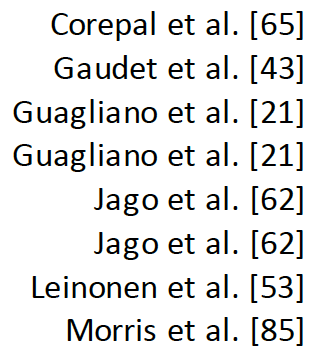


**
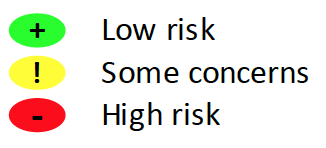
**
