# Supplementary material for: Effects of Consumer-Wearable Activity Tracker-Based Programs on Objectively Measured Daily Physical Activity and Sedentary Behavior Among School-Aged Children: A Systematic Review and Meta-analysis
Source: Sports Med Open. 2022 Jan 31;8:18. doi: 10.1186/s40798-021-00407-6 (PMC8804065; doi:10.1186/s40798-021-00407-6)
Supplement: Supplementary file 6 — Additional file 6. Certainty of the evidence assessment. [file 40798_2021_407_MOESM6_ESM.docx]

| Supplementary File 6. Certainty of the evidence assessment | | | | | | | | | | |
| --- | --- | --- | --- | --- | --- | --- | --- | --- | --- | --- |
|  | Unit of analysis | Number of participants | | Effect  *d* (95% CI) | Risk of bias | Inconsistency | Imprecision | Indirectness | Publication bias | Overall certainty |
|  |  | Intervention | Control |  |  |  |  |  |  |  |
| Total steps | 53 | 2,210 | 590 | 0.612 (0.477-0.746) | Serious^a,b^ | Serious^c^ | No limitation | No limitation | No limitation | Low |
| MVPA | 20 | 866 | 611 | 0.220 (0.134-0.307) | Serious^a^ | No limitation | No limitation | No limitation | No limitation | Moderate |
| Total PA | 8 | 534 | 434 | 0.151 (0.038-0.264) | Serious^a^ | No limitation | No limitation | No limitation | --^d^ | Moderate |
| Sedentary behavior | 8 | 481 | 399 | 0.172 (0.039-0.305) | Serious^a^ | No limitation | No limitation | No limitation | --^d^ | Moderate |
| *Note*. *d* = standardized mean difference*;* 95% CI = 95% confidence interval; MVPA = Moderate-to-vigorous physical activity; PA = Physical activity.  ^a^ Serious because > 50% of studies were assessed as High risk of bias  ^b^ Serious because > 50% of studies were pre-experimental trials  ^c^ Serious because of high statistical heterogeneity (*I*^2^ > 75%)  ^d^ Assessment of potential publication bias could not be carried out due to the limited number of studies (*k* < 10) | | | | | | | | | | |
