# Supplementary material for: Effects of Consumer-Wearable Activity Tracker-Based Programs on Objectively Measured Daily Physical Activity and Sedentary Behavior Among School-Aged Children: A Systematic Review and Meta-analysis
Source: Sports Med Open. 2022 Jan 31;8:18. doi: 10.1186/s40798-021-00407-6 (PMC8804065; doi:10.1186/s40798-021-00407-6)

Supplementary File 7. Results of the cumulative meta-analyses by study size for: (a) Daily total steps; and (b) Moderate-to-vigorous physical activity.

1.
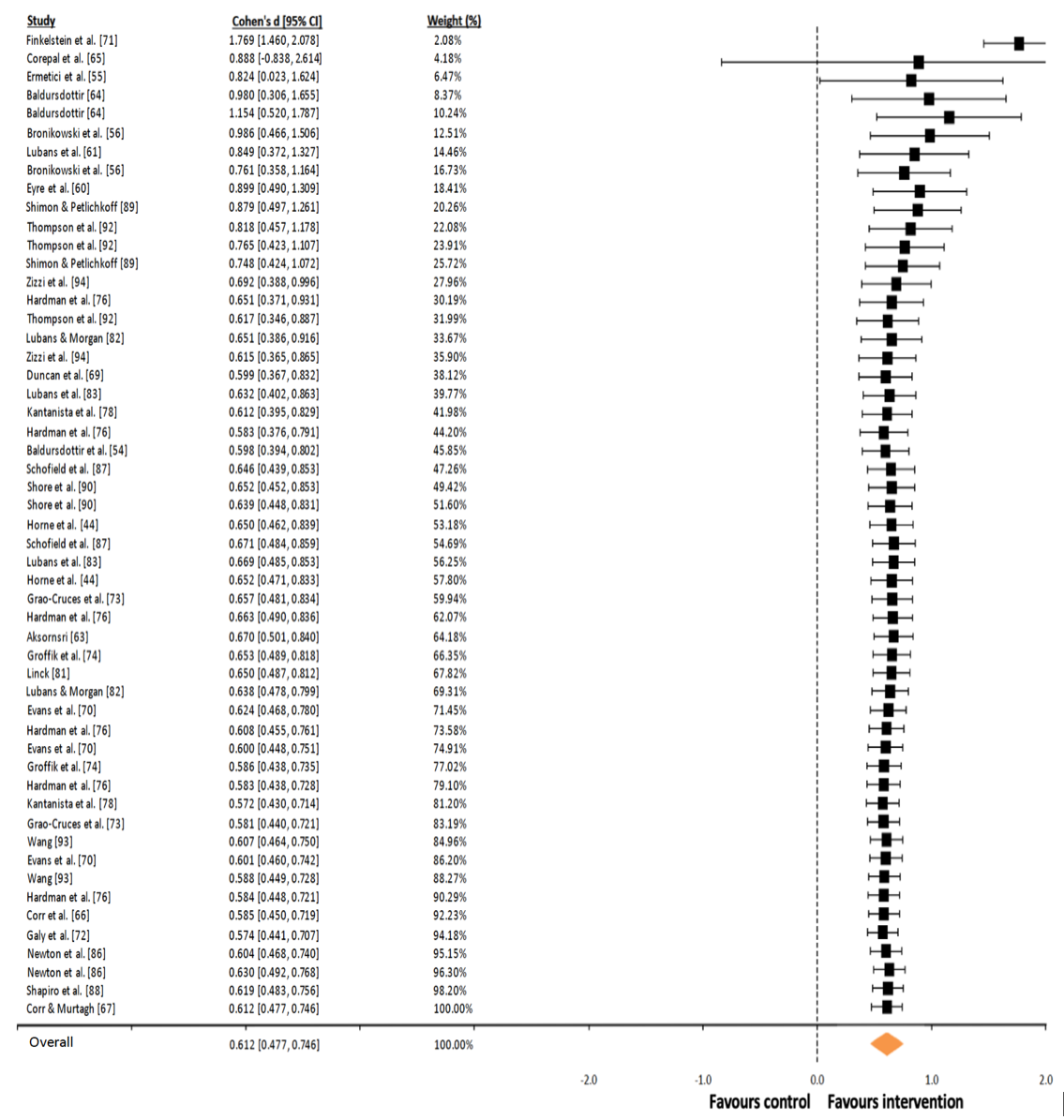

2.
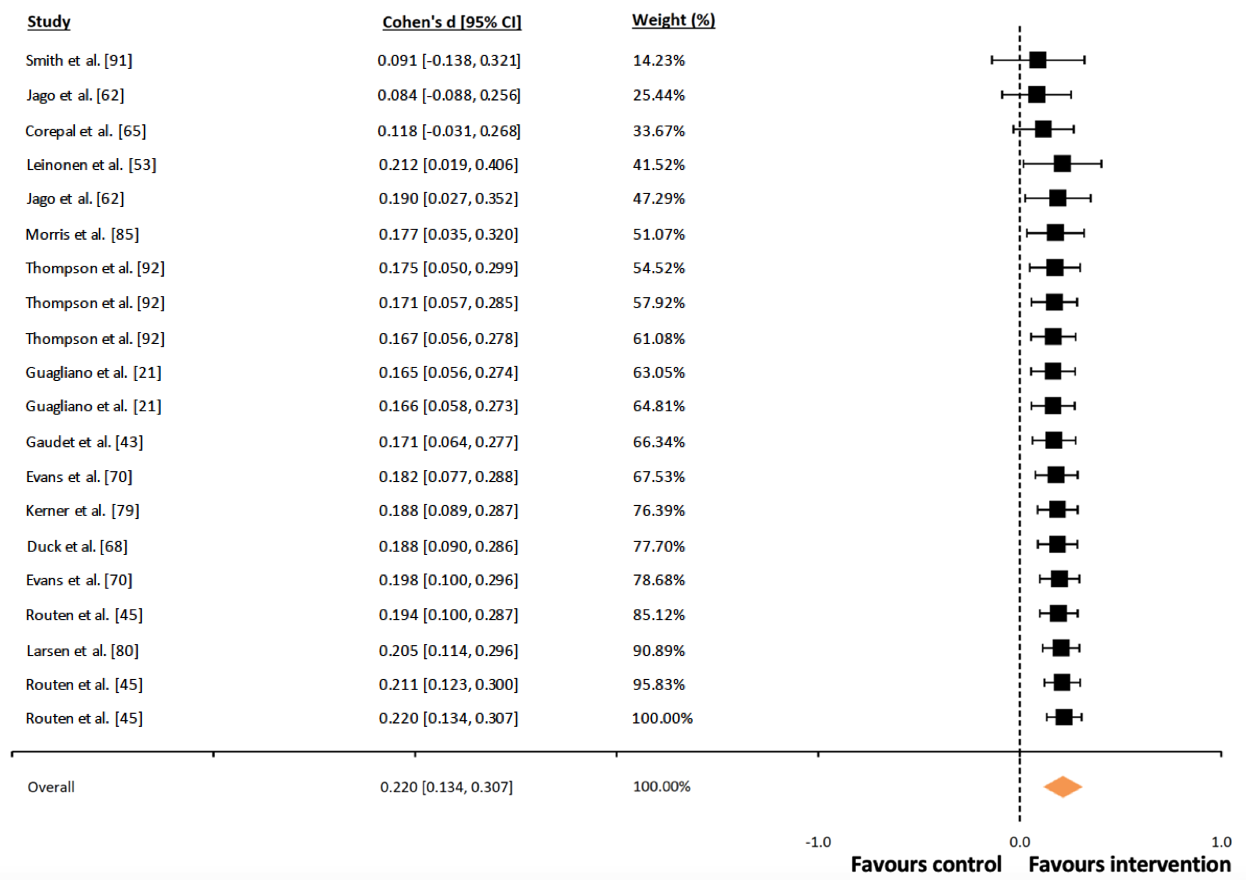

Supplement: Supplementary file 7 — Additional file 7. Results of the cumulative meta-analyses by study size for: (a) Daily total steps; and (b) Moderate-to-vigorous physical activity. [file 40798_2021_407_MOESM7_ESM.docx]
