# Supplementary material for: Effects of Consumer-Wearable Activity Tracker-Based Programs on Objectively Measured Daily Physical Activity and Sedentary Behavior Among School-Aged Children: A Systematic Review and Meta-analysis
Source: Sports Med Open. 2022 Jan 31;8:18. doi: 10.1186/s40798-021-00407-6 (PMC8804065; doi:10.1186/s40798-021-00407-6)
Supplement: Supplementary file 8 — Additional file 8. Results of the within-study subgroups analyses for the effect of the consumer-wearable activity tracker-based programs on the daily total steps among school-aged children. [file 40798_2021_407_MOESM8_ESM.docx]

| Supplementary File 8. Results of the within-study subgroups analyses for the effect of the consumer-wearable activity tracker-based programs on the daily total steps among school-aged children | | | | | | | | |
| --- | --- | --- | --- | --- | --- | --- | --- | --- |
| Moderator | Effects | *k* | *d* | 95% CI | *Z* | *p* | *I*^2^ | *p*-comparison |
| Sex | Males | 7 | 0.298 | 0.024, 0.572 | 2.134 | 0.033 | 69.32 | 0.187 |
|  | Females | 7 | 0.557 | 0.287, 0.828 | 4.040 | < 0.001 | 85.97 |  |
| Physical activity recommendations | Meeting | 2 | 0.107 | -0.170, 0.384 | 0.756 | 0.449 | 0.00 | < 0.001 |
|  | Not meeting | 2 | 1.206 | 0.912, 1.501 | 8.030 | < 0.001 | 0.00 |  |
| Goal-setting | Yes | 11 | 0.451 | 0.268, 0.633 | 4.831 | < 0.001 | 88.13 | 0.121 |
|  | No | 9 | 0.233 | 0.025, 0.439 | 2.212 | 0.027 | 15.03 |  |
| Diary | Yes | 6 | 0.527 | 0.068, 0.986 | 2.252 | 0.024 | 90.30 | 0.717 |
|  | No | 4 | 0.664 | 0.083, 1.245 | 2.239 | 0.025 | 93.46 |  |
| Counseling | Yes | 10 | 0.664 | 0.357, 0.971 | 4.244 | < 0.001 | 91.58 | 0.383 |
|  | No | 8 | 0.457 | 0.106, 0.807 | 2.551 | 0.011 | 84.04 |  |
| Reminders | Yes | 8 | 0.543 | 0.201, 0.886 | 3.110 | 0.002 | 88.85 | 0.840 |
|  | No | 5 | 0.601 | 0.154, 1.049 | 2.635 | 0.008 | 89.00 |  |
| Motivational strategies | Yes | 6 | 0.430 | 0.164, 0.696 | 3.172 | 0.002 | 86.07 | 0.530 |
|  | No | 4 | 0.291 | -0.053, 0.635 | 1.657 | 0.097 | 16.58 |  |
| *Note*. *k*, number of studies; *d* = standardized mean difference; 95% CI = 95% confidence interval; *I*^2^ = Higgins I-squared. Variables with less than two units of analysis to compare could not be analyzed. | | | | | | | | |
