# Supplementary material for: Effects of Consumer-Wearable Activity Tracker-Based Programs on Objectively Measured Daily Physical Activity and Sedentary Behavior Among School-Aged Children: A Systematic Review and Meta-analysis
Source: Sports Med Open. 2022 Jan 31;8:18. doi: 10.1186/s40798-021-00407-6 (PMC8804065; doi:10.1186/s40798-021-00407-6)
Supplement: Supplementary file 9 — Additional file 9. Results of the between-study subgroups analyses for the effect of the consumer-wearable activity tracker-based programs on the daily total steps among school-aged children. [file 40798_2021_407_MOESM9_ESM.docx]

| Supplementary File 9. Results of the between-study subgroups analyses for the effect of the consumer-wearable activity tracker-based programs on the daily total steps among school-aged children | | | | | | | | |
| --- | --- | --- | --- | --- | --- | --- | --- | --- |
| Moderator | Effects | *k* | *d* | 95% CI | *Z* | *p* | *I*^2^ | *p*-comparison |
| Sex | Males | 8 | 0.266 | -0.027, 0.558 | 1.779 | 0.075 | 66.08 | 0.044 |
|  | Females | 16 | 0.636 | 0.425, 0.848 | 5.891 | < 0.001 | 86.53 |  |
| Age | Children | 24 | 0.685 | 0.488, 0.881 | 6.818 | < 0.001 | 90.56 | 0.315 |
|  | Adolescents | 29 | 0.548 | 0.368, 0.728 | 5.970 | < 0.001 | 86.92 |  |
| Physical activity recommendations | Meeting | 24 | 0.397 | 0.254, 0.371 | 10.444 | < 0.001 | 72.40 | 0.003 |
|  | Not meeting | 29 | 0.795 | 0.613, 0.976 | 8.581 | < 0.001 | 92.54 |  |
| Duration | ≥ 8 weeks | 27 | 0.627 | 0.432, 0.823 | 6.289 | < 0.001 | 90.24 | 0.845 |
|  | < 8 weeks | 26 | 0.600 | 0.407, 0.792 | 6.111 | < 0.001 | 88.85 |  |
| Activity tracker type | Waist-worn | 47 | 0.652 | 0.507, 0.797 | 8.819 | < 0.001 | 89.97 | 0.115 |
|  | Wrist-worn | 6 | 0.303 | -0.105, 0.712 | 1.456 | 0.145 | 74.93 |  |
| Goal setting | Yes | 38 | 0.770 | 0.609, 0.932 | 9.349 | < 0.001 | 91.19 | < 0.001 |
|  | No | 15 | 0.243 | -0.003, 0.490 | 1.934 | 0.053 | 73.57 |  |
| Kind of goal setting | Static | 11 | 0.755 | 0.403, 1.107 | 4.201 | < 0.001 | 92.97 | 0.585 |
|  | Adaptive | 25 | 0.875 | 0.629, 1.120 | 6.994 | < 0.001 | 90.12 |  |
| Diary | Yes | 36 | 0.591 | 0.424, 0.757 | 6.965 | < 0.001 | 86.67 | 0.639 |
|  | No | 17 | 0.661 | 0.419, 0.903 | 5.357 | < 0.001 | 92.87 |  |
| Counseling | Yes | 36 | 0.711 | 0.550, 0.873 | 8.625 | < 0.001 | 90.69 | 0.003 |
|  | No | 17 | 0.407 | 0.179, 0.635 | 3.496 | < 0.001 | 78.65 |  |
| Reminders | Yes | 19 | 0.713 | 0.484, 0.941 | 6.108 | < 0.001 | 85.86 | 0.305 |
|  | No | 34 | 0.558 | 0.391, 0.725 | 6.552 | < 0.001 | 90.40 |  |
| Motivational strategies | Yes | 25 | 0.744 | 0.544, 0.944 | 7.305 | < 0.001 | 90.41 | 0.062 |
|  | No | 28 | 0.501 | 0.318, 0.683 | 5.373 | < 0.001 | 88.00 |  |
| Exercise routine | Yes | 9 | 0.727 | 0.390, 1.064 | 4.230 | < 0.001 | 87.77 | 0.461 |
|  | No | 44 | 0.589 | 0.443, 0.736 | 7.891 | < 0.001 | 89.64 |  |
| Sum of behavior change strategies | ≥ 4 strategies | 26 | 0.652 | 0.453, 0.851 | 6.424 | < 0.001 | 86.65 | 0.598 |
|  | < 4 strategies | 27 | 0.578 | 0.390, 0.766 | 6.024 | < 0.001 | 91.41 |  |
| *Note*. *k*, number of studies; *d* = standardized mean difference; 95% CI = 95% confidence interval; *I*^2^ = Higgins I-squared. | | | | | | | | |
