# Supplementary material for: Effects of Consumer-Wearable Activity Tracker-Based Programs on Objectively Measured Daily Physical Activity and Sedentary Behavior Among School-Aged Children: A Systematic Review and Meta-analysis
Source: Sports Med Open. 2022 Jan 31;8:18. doi: 10.1186/s40798-021-00407-6 (PMC8804065; doi:10.1186/s40798-021-00407-6)
Supplement: Supplementary file 10 — Additional file 10. Results of the within-study subgroups analyses for the effect of the consumer-wearable activity tracker-based programs on the daily moderate-to-vigorous physical activity among school-aged children. [file 40798_2021_407_MOESM10_ESM.docx]

| Supplementary File 10. Results of the within-study subgroups analyses for the effect of the consumer-wearable activity tracker-based programs on the daily moderate-to-vigorous physical activity among school-aged children | | | | | | | | |
| --- | --- | --- | --- | --- | --- | --- | --- | --- |
| Moderator | Effects | *k* | *d* | 95% CI | *Z* | *p* | *I*^2^ | *p*-comparison |
| Sex | Males | 2 | 0.177 | -0.059, 0.413 | 1.473 | 0.141 | 0.00 | 0.395 |
|  | Females | 2 | 0.343 | 0.043, 0.642 | 2.242 | 0.025 | 0.00 |  |
| Goal-setting | Yes | 4 | 0.269 | 0.031, 0.507 | 2.214 | 0.027 | 0.00 | 0.459 |
|  | No | 3 | 0.423 | 0.090, 0.756 | 2.492 | 0.013 | 22.01 |  |
| Diary | Yes | 4 | 0.269 | 0.031, 0.507 | 2.214 | 0.027 | 0.00 | 0.459 |
|  | No | 3 | 0.423 | 0.090, 0.756 | 2.492 | 0.013 | 22.01 |  |
| Reminders | Yes | 3 | 0.138 | -0.161, 0.437 | 0.905 | 0.366 | 0.00 | 0.992 |
|  | No | 2 | 0.136 | -0.238, 0.509 | 0.711 | 0.477 | 0.00 |  |
| *Note*. *k*, number of studies; *d* = standardized mean difference; 95% CI = 95% confidence interval; *I*^2^ = Higgins I-squared. Variables with less than two units of analysis to compare could not be analyzed. | | | | | | | | |
