# Supplementary material for: Effects of Consumer-Wearable Activity Tracker-Based Programs on Objectively Measured Daily Physical Activity and Sedentary Behavior Among School-Aged Children: A Systematic Review and Meta-analysis
Source: Sports Med Open. 2022 Jan 31;8:18. doi: 10.1186/s40798-021-00407-6 (PMC8804065; doi:10.1186/s40798-021-00407-6)
Supplement: Supplementary file 11 — Additional file 11. Results of the between-study subgroups analyses for the effect of the consumer-wearable activity tracker-based programs on the daily moderate-to-vigorous physical activity among school-aged children. [file 40798_2021_407_MOESM11_ESM.docx]

| Supplementary File 11. Results of the between-study subgroups analyses for the effect of the consumer-wearable activity tracker-based programs on the daily moderate-to-vigorous physical activity among school-aged children | | | | | | | | |
| --- | --- | --- | --- | --- | --- | --- | --- | --- |
| Moderator | Effects | *k* | *d* | 95% CI | *Z* | *p* | *I*^2^ | *p*-comparison |
| Sex | Males | 6 | 0.173 | 0.054, 0.293 | 2.847 | 0.004 | 22.45 | 0.167 |
|  | Females | 3 | 0.356 | 0.126, 0.587 | 3.032 | 0.002 | 0.00 |  |
| Age | Children | 10 | 0.211 | 0.076, 0.347 | 3.056 | 0.002 | 0.00 | 0.866 |
|  | Adolescents | 10 | 0.227 | 0.114, 0.339 | 3.947 | < 0.001 | 0.00 |  |
| Physical activity recommendations | Meeting | 5 | 0.404 | 0.214, 0.593 | 4.172 | < 0.001 | 29.06 | 0.046 |
|  | Not meeting | 10 | 0.170 | 0.042, 0.298 | 2.601 | 0.009 | 0.00 |  |
| Duration | ≥ 8 weeks | 12 | 0.189 | 0.084, 0.294 | 3.527 | < 0.001 | 0.00 | 0.296 |
|  | < 8 weeks | 8 | 0.288 | 0.135, 0.441 | 3.680 | < 0.001 | 0.00 |  |
| Activity tracker type | Waist-worn | 14 | 0.167 | 0.069, 0.265 | 3.340 | 0.001 | 0.00 | 0.021 |
|  | Wrist-worn | 6 | 0.413 | 0.228, 0.599 | 4.363 | < 0.001 | 3.43 |  |
| Goal-setting | Yes | 12 | 0.212 | 0.110, 0.313 | 4.089 | < 0.001 | 0.00 | 0.752 |
|  | No | 8 | 0.243 | 0.077, 0.409 | 2.876 | 0.004 | 0.00 |  |
| Kind of goal-setting | Static | 4 | 0.254 | 0.068, 0.440 | 2.676 | 0.007 | 61.62 | 0.450 |
|  | Adaptive | 6 | 0.162 | 0.010, 0.313 | 2.089 | 0.037 | 0.00 |  |
| Diary | Yes | 7 | 0.214 | 0.081, 0.347 | 3.162 | 0.002 | 0.00 | 0.903 |
|  | No | 13 | 0.225 | 0.111, 0.339 | 3.861 | < 0.001 | 0.00 |  |
| Counseling | Yes | 12 | 0.200 | 0.095, 0.305 | 3.739 | < 0.001 | 0.00 | 0.508 |
|  | No | 8 | 0.263 | 0.110, 0.416 | 3.369 | 0.001 | 0.00 |  |
| Reminders | Yes | 5 | 0.163 | 0.000, 0.325 | 1.965 | 0.049 | 0.00 | 0.414 |
|  | No | 15 | 0.243 | 0.141, 0.345 | 4.658 | < 0.001 | 0.00 |  |
| Motivational strategies | Yes | 8 | 0.265 | 0.132, 0.398 | 3.907 | < 0.001 | 9.30 | 0.383 |
|  | No | 12 | 0.187 | 0.073, 0.301 | 3.222 | 0.001 | 0.00 |  |
| Exercise routine | Yes | 3 | 0.083 | -0.072, 0.238 | 1.049 | 0.294 | 0.00 | 0.036 |
|  | No | 17 | 0.283 | 0.178, 0.387 | 5.309 | < 0.001 | 0.00 |  |
| Sum of behavior change strategies | ≥ 4 strategies | 5 | 0.212 | 0.057, 0.367 | 2.683 | 0.007 | 6.03 | 0.901 |
|  | < 4 strategies | 15 | 0.224 | 0.120, 0.328 | 4.208 | < 0.001 | 0.00 |  |
| *Note*. *k*, number of studies; *d* = standardized mean difference; 95% CI = 95% confidence interval; *I*^2^ = Higgins I-squared. | | | | | | | | |
